# Prebiotically Plausible Synthesis of *N*‑Cyanoimidazole for Phosphate Activation and Templated RNA Ligation

**DOI:** 10.1021/jacs.6c16138

**Published:** 2026-08-25

**Authors:** Bruno Marinic, Dougal J. Ritson, Robin Wordsworth, John D. Sutherland

**Affiliations:** † MRC Laboratory of Molecular Biology, 47694 Cambridge Biomedical Campus, Francis Crick Avenue, Cambridge CB2 0QH, U.K.; ‡ Department of Earth and Planetary Sciences, 1812 Harvard University, Cambridge, Massachusetts 02138, United States

## Abstract

Phosphate activation is a key step in the transition from mononucleotides and short oligonucleotides to polynucleotides by primer-extension and/or ligation. Prebiotically plausible phosphate activating reagents have remained elusive, with potential candidates limited by either poor reactivity or uncertain prebiotic provenance. Here we report a prebiotically plausible synthesis of *N*-cyanoimidazole, a known and efficient phosphate activating reagent, by reaction of thiocyanogen with imidazole. We further demonstrate that *N*-cyanoimidazole can effect mononucleotide activation and highly efficient RNA ligation in nicked duplexes with a range of oligomers including trimers and dimers.

## Graphical abstract

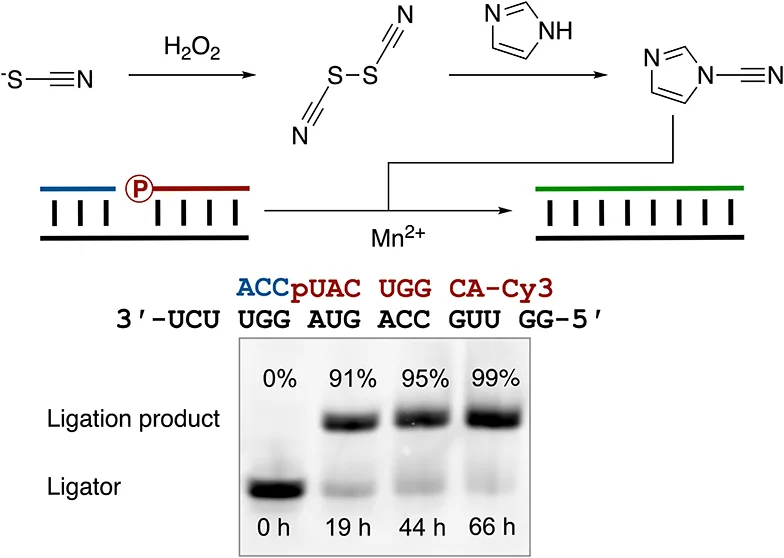

The formation of oligonucleotides from nucleotide building blocks through phosphate activation and O–P bond formation is thought to represent a key step in the synthesis of the first functional biopolymers.^1–5^ Cyanamide has emerged as a popular candidate as a prebiotic activating agent, as its diimide tautomer resembles *N*,*N′*-dialkylcarbodiimides – commonly used condensing reagents in modern-day synthetic chemistry. While the prebiotic plausibility of cyanamide is high, its reactivity is poor, thereby requiring high concentrations, elevated temperatures and long reaction times to provide substantial phosphate activation.^6–9^ We found the use of glyoxylate or pyruvate as catalyst greatly improves the reactivity of cyanamide toward phosphate activation, obtaining 95% adenosine-2′,3′-cyclic monophosphate (2′,3′-cAMP) from adenosine 3′-monophosphate (3′-AMP).^10^ However, we were unable to trap the activated 5′-phosphate with a nucleophile other than water, meaning the system was unlikely to enable ligation chemistry or primer-extension chemistry with 5′-phosphates. We have also shown how methyl isonitrile could have been produced in an environment that is able to deliver methylamine to a mixture of ferrocyanide and nitroprusside, itself formed from ferrocyanide and nitrite.^11^ The demonstrated proficiency of methyl isonitrile for direct mononucleotide activation, RNA monomer extension, and RNA ligation made it a promising candidate for prebiotic phosphate activation.^12–15^ However, it now seems unlikely that substantial quantities of nitrite would have been available on early Earth.^16,17^ Efficient ligation in a nicked duplex RNA system requires the favorable arrangement of a 3′-hydroxyl group and an activated 5′-phosphate group on abutting strands of RNA. The bridged dinucleotide approach pioneered by Szostak et al. and Richert et al. for primer-extension chemistry benefits from double binding enforcing this conformation.^18–20^ Issues in alignment are most likely responsible for low yields of RNA ligation reactions, even when using large excesses of coupling reagents.^21^ However, when *N*-cyanoimidazole (NCI) is employed as the activating agent, good yields of ligation products are observed in a range of nucleic acid systems.^16,22–26^ Furthermore, its imidazole adduct *N*,*N′*-iminediimidazole (IDI) has been used to prepare nucleotide imidazolides in good yields, albeit at extremely high concentrations of all species involved.^27^ Until now, a convincing prebiotic synthesis of NCI has remained elusive. Most syntheses involve cyanogen bromide, which would not have been present on primitive Earth, or the generation of cyanogen chloride, which, while slightly more plausible, requires precise control of reagent stoichiometry (NC^–^ and ClO^–^) and their codelivery to prevent unwanted side reactions.^28–30^ Given that NCI efficiently enables ligation and phosphorimidazolide formation, we determined that a comprehensive investigation of possible sources of NCI be carried out within the constraints of the cyanosulfidic scenario we have described as a guide to available reagents.

Our attention initially focused on cyanogen (NC)_2_, which we had observed as a product of the photooxidation of NC^–^ by catalytic copper salts. We wondered if displacement of NC^–^ from (NC)_2_ ^31,32^ by imidazole might lead to NCI.^33^ Unfortunately, the reaction of imidazole with (NC)_2_ did not result in formation of NCI nor IDI but instead gave a compound that we tentatively assign as cyanoformimidoylimidazole (CFI, Figure 1A and Figures S2–S9). (Further experimental details and discussion can be found in the Supporting Information.)

**Figure 1:**
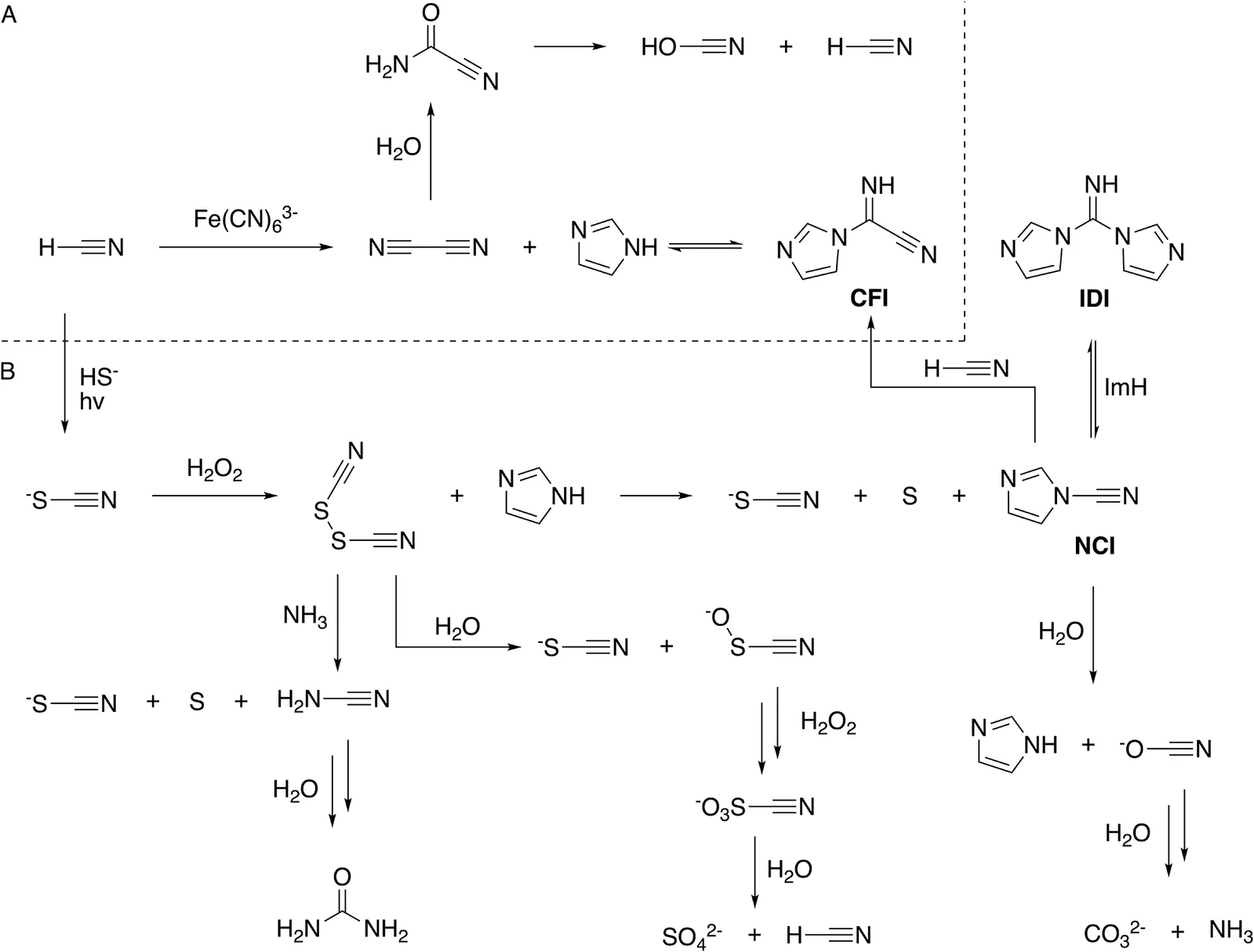
Chemistry of (A) cyanogen-imidazole systems and (B) thiocyanogen-imidazole systems.

Keefe and Miller reported an underexplored example of phosphate activation where aqueous inorganic phosphate, hydrogen peroxide and thiocyanate (NCS^–^) gave yields of pyrophosphate up to 26% at room temperature.^34^ The cyanosulfidic scenario can account for the formation of NCS^–^, which is readily produced by simple UV irradiation of HCN and hydrosulfide.^14^ While there is still debate on the prebiotic plausibility of H_2_O_2_, we thought it worth exploring this observation from the literature further.^35^ Keefe and Miller postulated that thiocyanogen ((NCS)_2_) or hypothiocyanous acid (HOSCN) might be responsible for the conversion of phosphate to pyrophosphate. It has been reported that imidazole emerges unscathed after exposure to thiocyanogen when attempting its C-thiocyanation.^36^ However, if instead NCI had been formed, it would have been hydrolyzed back to imidazole by the workup procedure employed. Using ^13^C NMR spectroscopy in conjunction with ^13^C-labeled reagents we set out to elucidate the steps involved in the activation reaction reported by Keefe and Miller and to establish if such chemistry could be used to generate NCI.

We started our investigation by treating ^13^C-labeled KSCN (100 mM) with H_2_O_2_ (200 mM) and following the reaction by ^13^C NMR spectroscopy (Figure 2A). After 4 h 70% conversion and three major products were observed, and their chemical shifts matched reports from the literature for: (NCS)_2_ (48%),^37^ NCSO^–^ (11%),^38^ and HCN (13%). Cyanide is produced alongside sulfate as the decomposition product of sulfocyanide (NCSO_3_^–^), formed upon further oxidation of NCSO^–^ by H_2_O_2_. The production of sulfate also accounts for the significant drop in pH from 7 to 2.5. Small amounts of cyanamide and urea can be observed if the reaction is monitored for extended periods of time (Figure S10C. See Supporting Information for further experimental details). Next, we subjected ^13^C-labeled (NCS)_2_ to addition of imidazole, which raised the solution pH to 7. NMR spectroscopy showed a peak at δ 143 ppm indicating the formation of IDI via NCI (Figure 2B). If the reaction pH was adjusted to pH 6, both NCI and IDI peaks could be observed in the ^1^H NMR spectrum (see Supporting Information for further experimental details). We next investigated RNA ligation in a nicked duplex using NCI as the initial NCI-5′-phosphate adduct has been shown to be effective for ligation.^16^

**Figure 2:**
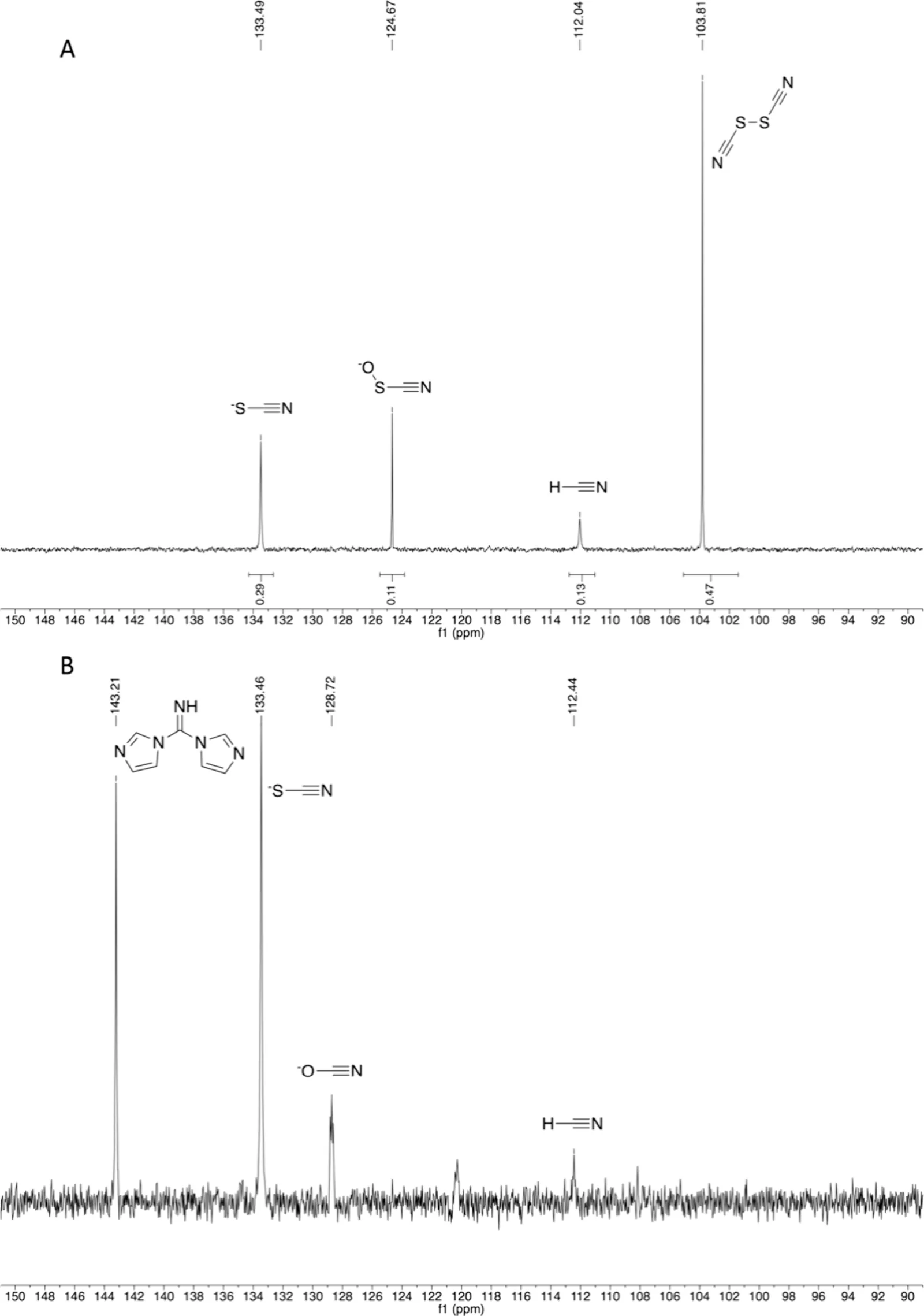
(A) ^13^C NMR spectrum of 100 mM KS^13^CN with 200 mM H_2_O_2_, after 8 h at r.t.; δ 104 ppm = (NCS)_2_, δ 112 ppm = HCN, δ 124 ppm = NCSO^–^, and δ 133 ppm = NCS^–^; (B) ^13^C NMR spectrum of the reaction of 50 mM (N^13^CS)_2_ with 100 mM imidazole at pH 7. δ 112 ppm = HCN, δ 128 ppm = NCO^**‑**^, δ 133 ppm = NCS^–^, and δ 143 ppm = IDI (iminyl carbon).

Initially employing commercial NCI in the absence of divalent metal ions we observed no ligation. Previously, we had found 10 mM Mn^2+^ gave a good ligation yield (78%) when using NCI in the same nicked duplex system.^16^ Based on literature evidence suggesting metal chelate involvement in the hydrolysis of RNA backbones,^39^ we propose the coordination of the NCI activated 5′-phosphate and the neighboring nucleotide phosphodiester with Mn^2+^ to hold the nucleotide in a reactive conformation (Figure 3).

**Figure 3:**
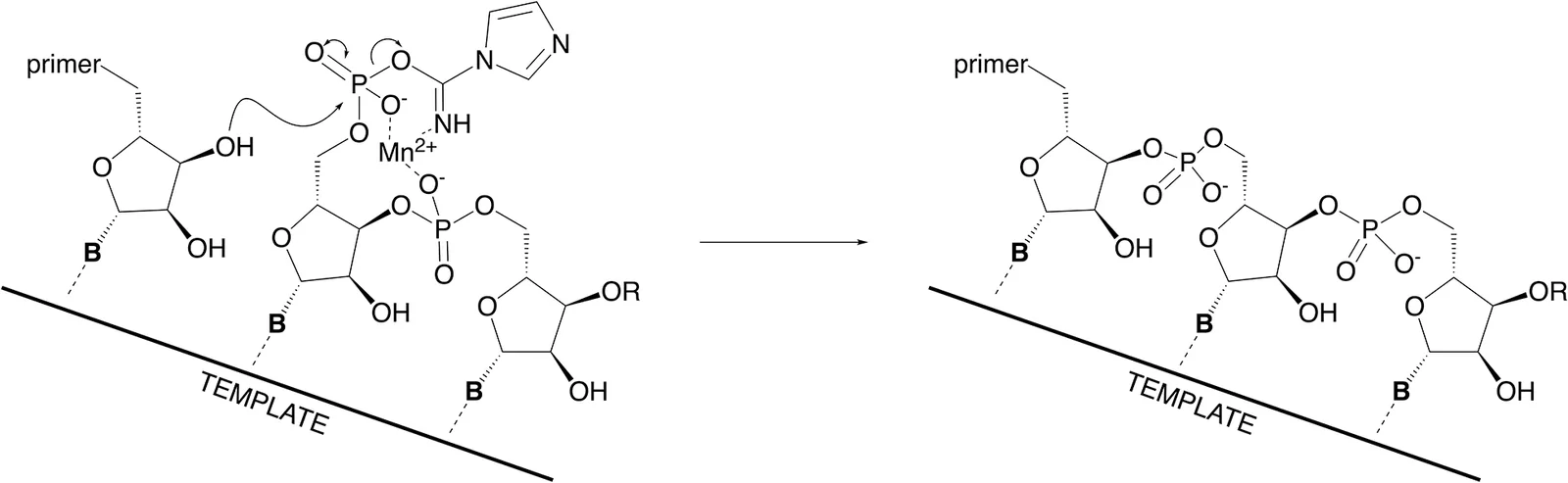
Proposed 5′-phosphate-NCI adduct chelating with Mn^2+^ and the 3′,5′-phosphodiester linkage.

Following extensive optimization, details of which can be found in the Supporting Information, 42% ligation could be observed after 2 h going up to 89% after 8 h and to 99% after 48 h (Figure 4A and Figure S35) (all ligation reactions were performed at room temperature unless otherwise stated). Performing the same reaction using a FAM-labeled ligator gave a comparable result, showing that the fluorescent label does not affect the outcome of this chemical ligation (Figure S38). We then attempted a ligation reaction under these conditions, but using our prebiotic route to form NCI/IDI, and at a range of Mn^2+^ concentrations (as we assumed excess imidazole and NCS^–^ present in the NCI/IDI mixture would coordinate and sequester Mn^2+^ if at low concentrations). At 50 mM Mn^2+^ we observed 44% ligation product after 43 h and 50% after 65 h. Further increases in manganese concentration did not improve the yield or rate of the ligation (Figures S39–S40). Wanting to quantify any off-template reactivity, we performed the same reaction in the absence of RNA template. No products were observed within 48 h (for details on the use of other activating reagents under these optimized conditions see the Supporting Information)

**Figure 4:**
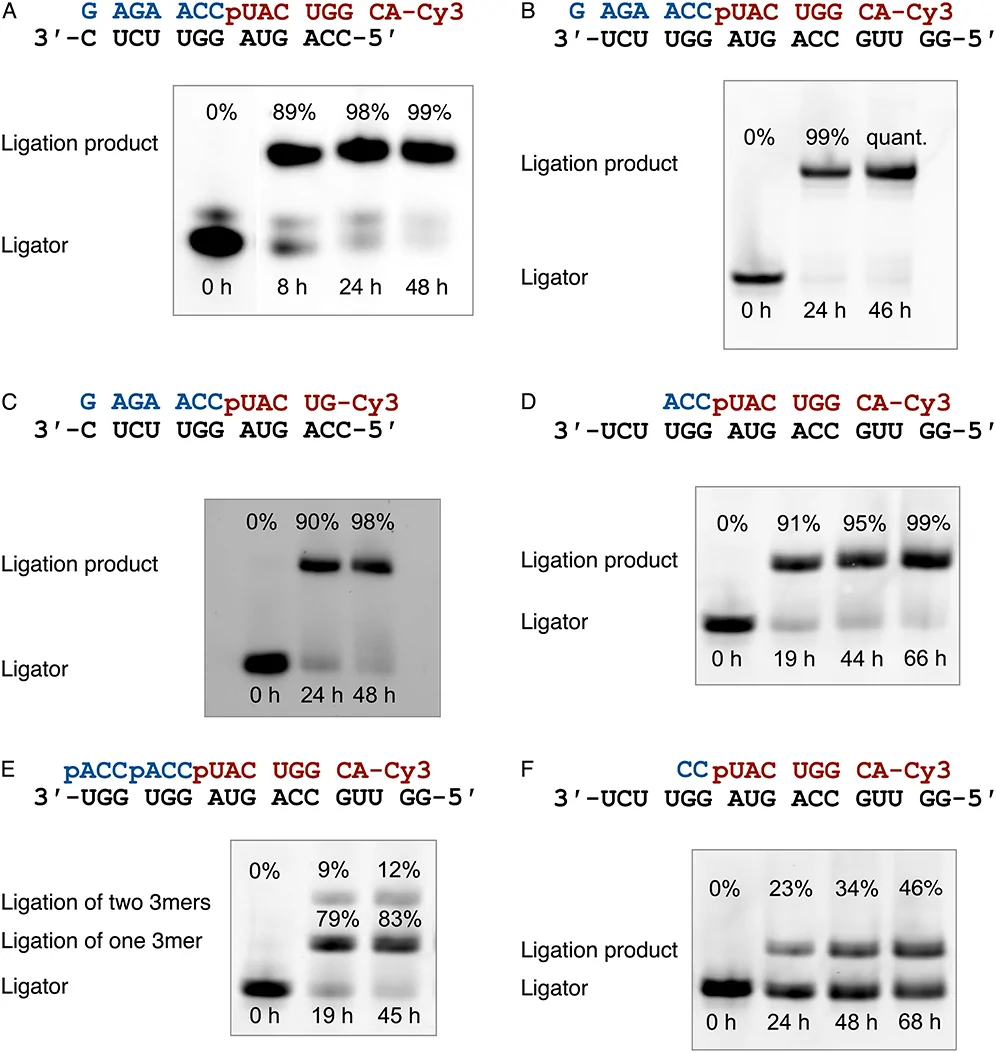
PAGE analysis of *in situ* nicked-duplex RNA ligations performed at room temperature (unless otherwise stated) with NaCl (100 mM), MnCl_2_ (20 mM), imidazole buffer pH 6.2 (20 mM), and NCI (20 mM). (A) 5′-pUACUGGCA-Cy3 (10 μM), 5′-GAGAACC (20 μM), 5′-CCAGUAGGUUCUC (10 μM). Selected time points only full gel in the ESI Figure S35; (B) 5′-pUACUGGCA-Cy3 (10 μM), 5′-GAGAACC (20 μM), 5′-GUUCCAGUAGGUUCU (10 μM); (C) 5′-pUACUG-Cy3 (10 μM), 5′-GAGAACC (20 μM), 5′-CCAGUAGGUUCUC (10 μM); (D) 5′-ACC (100 μM), 5′-pUACUGGCA-Cy3 (10 μM), 5′-GUUCCAGUAGGUUCU (20 μM); (E) 5′-pACC (100 μM), 5′-pUACUGGCA-Cy3 (10 μM), 5′-GUUCCAGUAGGUGGU (20 μM); (F) 5′-CC (1 mM), 5′-pUACUGGCA-Cy3 (10 μM), 5′-GUUCCAGUAGGUUCU (10 μM), at 4 °C.

In theory the new phosphodiester bond can form to give either the canonical 3′-5′ or the undesired 2′-5′ linkage. To determine the ratio of 2′-5′ to 3′-5′ phosphodiester bonds formed in our ligation system we adopted an RNase T2 digestion method reported by Ferris et al.^40^ We were pleased to observe exclusive formation of the canonical 3′-5′ linkage (Figure S43). A detailed discussion of the mechanism of the ligation reaction is available in the Supporting Information (Figure S44). Having established optimized conditions and confirmed the regioselectivity of the ligation we next explored the scope and limitations of our method. A control reaction in the absence of template did not give any ligation product (Figure S47). To interrogate the system further we began to shorten the ligator, and were pleased to find a minimal drop in efficiency of a 5mer ligation which gave the product in 98% yield after 48 h (Figure 4C). Ligation of the trinucleotide ACC also proceeded in almost quantitative yield (99%) after 66 h (Figure 4D). When using the 5′-phosphorylated trimer pACC, 86% yield of ligated product was observed when the trinucleotide was used as the primer i.e. ligation proceeding in the 3′ to 5′ direction (Figure S50). However, when pACC was used as the ligator i.e. ligation running in the 5′ to 3′  direction, no ligation was observed (Figure S51). We attributed this loss of reaction to the lower residence time of the pACC trinucleotide resulting in hydrolysis of the nontemplate-bound 5′-phosphate NCI adduct, which is key for ligation to occur. The sequential ligation of two pACC trinucleotides resulted in incorporation of the first trinucleotide in an excellent yield of 95% although the second addition only proceeded in 12% yield after 45 h (Figure 4E). Finally, we observed a major drop in yield when attempting to ligate the dinucleotide CC, with only 6% of the product observed after 46 h (Figure S53). However, increasing the concentration of CC to 1 mM and performing the reaction at 4 °C gave the ligation product in 46% after 68 h (Figure 4F). These results clearly show the efficiency of our chemical system for the ligation of various lengths of oligonucleotides.

Overall, we have presented a plausible scheme for phosphate activation that relies on (NCS)_2_, which is produced by oxidation of NCS^–^, and this in turn is consistent with cyanosulfidic system we have previously reported.^6,14^ While H_2_O_2_ was employed as the oxidizing agent, we recognize that there are potentially other ways of generating (NCS)_2_ by oxidation. An attractive possibility that we are currently exploring is UV-photoredox, as (NCS)_2_ exhibits remarkable stability when irradiated at 310 nm or higher. We have shown that (NCS)_2_ reacts with imidazole to form NCI, a potent phosphate activating agent, capable of monophosphate activation, phosphoimidazolide formation and RNA ligation. We have previously explored different termini in the RNA ligation chemistry: we published work on a 5′-OH and 3′-phosphate RNA ligation, using NCI and Mn^2+^, enabled by prior selective acetylation of the 2′-OH group.^25,26^ We have also previously reported an initial unoptimized NCI/Mn^2+^ ligation of a 5′-phosphate and 2′,3′-diol, however, this was not explored in depth.^16^ The directionality of RNA replication also has implications for the single versus sequential addition of an activating agent to the system. In the 3′ to 5′ replication demonstrated in this paper, the growing chain ends in a 5′-phosphate which must be activated to add an unactivated oligonucleotide, generating a new terminal unactivated 5′-phosphate. For continued chain growth, sequential addition of the activating agent appears essential in this scheme. In contrast, in the canonical replication direction, the growing chain ends in a 3′-OH, which is sequentially elongated by reaction with a pool of 5′-activated substrates, ostensibly suggesting that this scheme could operate with a single addition of activating agent. However, hydrolysis of the pool of 5′-activated substrates would severely limit replication as time progresses. Thus, realistically, both replication schemes would function (best) with sequential addition of activating agents.

We note that a thorough investigation of the NCI/M^2+^ system for the chemical ligation of DNA and L-aTNA was carried out by Asanuma and Murayama.^23^ While we operated under prebiotic constraints in our studies of RNA ligation, Murayama et al., who were not subject to the same constraints, explored a wider range of M^2+^ ions and highlight a greater promiscuity of the DNA and L-aTNA ligation systems with regard to the nature of the metal ion as compared to the RNA systems we have explored herein. We have demonstrated high yielding RNA ligation with a wide range of oligonucleotides including trimers and dimers. NCI/IDI activation is known to lead to extensive 2′-OH modification at pH 8.^41^ Mitigation of this 2′-modification requires that RNA replication chemistry that utilizes NCI take place at pH 6. One added benefit of a low pH ligation regime is that background M^2+^-mediated backbone hydrolysis that besets higher pH primer extension chemistry is limited. Beyond its perceived relevance to prebiotic chemistry, the ligation chemistry described herein potentially has applicability in broad scope RNA chemistry, such as replicative amplification chemistry. Further work on sequential trinucleotide ligations and exploration of amino acid reactivity with NCI are also currently ongoing.

## Supplementary Material



## Notes

Experimental procedures, NMR spectra, full PAGE analysis, and detailed discussion of cyanogen, thiocyanogen, and NCI reactivity, ligation mechanism, and ligation optimization (PDF)
