# Supplementary material for: Prebiotically Plausible Synthesis of N‑Cyanoimidazole for Phosphate Activation and Templated RNA Ligation
Source: J Am Chem Soc. 2026 Aug 25;148(35):37623–7. doi: 10.1021/jacs.6c16138 (PMC13564439; doi:10.1021/jacs.6c16138)
Supplement: Supplementary file 1 [file ja6c16138_si_001.pdf]

Supporting Information

Prebiotically plausible synthesis of *N*-cyanoimidazole for phosphate activation and templated RNA ligation

Bruno Marinic[a], Dougal J. Ritson[a], Robin Wordsworth[b] and John D. Sutherland[a,*]

[a]MRC Laboratory of Molecular Biology, Francis Crick Avenue, Cambridge Biomedical Campus, Cambridge CB2 0QH, UK

[b]Department of Earth and Planetary Sciences, Harvard University, Cambridge, MA 02138, US.

*Corresponding Author: johns@mrc-lmb@cam.ac.uk

Experimental Procedures and NMR Spectra

Materials and Methods

Reagents and solvents were bought from Sigma-Aldrich, Alfa Aesar and Santa Cruz Biotechnology and were used without further purification unless otherwise stated. ^{13}C-Labelled potassium cyanide ($K^{13}CN$) and ^{13}C-labelled potassium thiocyanate ($KS^{13}CN$) had an isotopic incorporation of 99%. Phosphoramidites for RNA synthesis were purchased from Sigma-Aldrich or Link Technologies. The Cyanine3 (Cy3) labelled oligonucleotides were purchased in HPLC-purified Na^+ form from Integrated DNA Technologies. The non-labelled oligonucleotide was synthesized using an ÄKTA oligopilot plus 10 instrument (GE Healthcare). A Mettler Toledo SevenEasy pH Meter S20 was used to monitor pH, and deoxygenation of solutions was achieved by sparging with argon for 20 min. ^{1}H- ^{31}P- and ^{13}C-NMR spectra were acquired using a Bruker Ultrashield 400 Plus machine (at 400.1 MHz, 162.0 and 100.6 MHz respectively). Samples consisting of H_2O/D_2O mixtures were analysed using HOD/H_2O suppression to collect ^{1}H-NMR data. Coupling constants (*J*) are given in Hertz and the notations s, t, and br s represent the multiplicities singlet, triplet, and broad signal. Chemical shifts (δ) are given in ppm. Gel electrophoresis experiments using 20% polyacrylamide and 8 M urea gels (8 cm x 8 cm x 1 mm) were typically run at 6 W in Tris/Borate/EDTA buffer. Fluorescence imaging was performed using an Amersham Typhoon imager (GE Healthcare) and quantified using Image Quant TL software (version 7.0). Oligonucleotide concentrations were determined by ultraviolet absorbance at 260 nm using a NanoDrop ND-1000 spectrophotometer.

Chemical synthesis of RNA oligomers

After automated synthesis, RNAs were first cleaved from the solid support by treatment with a 1:1 mixture (3 mL) of aqueous NH_3 solution (28% wt.) and an ethanol solution of CH_3NH_2 (33% wt.) at 55 °C for 90 min in a tube with a sealed cap. The solid was removed by filtration and washed with 50% $EtOH/H_2O$. The solutions were combined and evaporated to dryness under reduced pressure. Silyl protecting groups were removed by treating the residues with a 1:1 mixture of triethylamine trihydrofluoride and DMSO (2 mL) at 65 °C for 3 h in a tube with a sealed cap. After brief cooling at -32 °C, cold $NaClO_4$ in acetone (40 mL, 50 mM) was added to the solution to precipitate the oligoribonucleotides. The resulting mixture was centrifuged and oligoribonucleotides were dried by freezing and lyophilisation. The RNA was

redissolved in H_2O (5 mL) and passed through a Waters Sep–Pak C18 Cartridge with 10 g sorbent. The cartridge was prewashed with MeCN (50 mL) then H_2O (50 mL) before the sample was loaded, and then the product was eluted by washing with H_2O (150 mL) then 10 % aqueous MeCN (50 mL). Eluates were checked for RNA content using a NanoDrop spectrophotometer. After lyophilising, the resulting white powder was stored at -32 °C for future use.

General procedure for desalting of RNA by ethanol precipitation

Oligonucleotides were desalted by addition of imidazole nitrate solution (2 M, pH 6.2, 1/10 the volume of the aliquot that was taken from the reaction), followed by a sodium acetate solution (3 M, pH 5.2, 1/10 the volume of the aliquot that was taken from the reaction) and absolute ethanol (to a final concentration of 75 % (v/v)). The resulting mixture was kept at -20 °C for 3 h and then centrifuged for 30 min at 16,000g. The supernatant was removed, and the pellets were washed with aqueous ethanol (75 % (v/v)) before additional centrifugation (10 min at 16,000g). The resulting pellets were air dried before being re-dissolved in water.

General procedure for oligonucleotide ligation reactions

To an aqueous solution of RNAs, NaCl (100 mM), organocatalysts, and divalent metal ion was added nuclease-free water to reach total volume of 10 µL after the addition of the phosphate activating agent. Aliquots (1.0 µL) were taken at the indicated time points and mixed with loading dye (9 µL, 10 M urea in formamide, Orange G). The resulting mixture was analysed using polyacrylamide gel electrophoresis.

Reactions involving cyanogen and *N,N′*-iminediimidazole (IDI)

Discussion of the reactions involving cyanogen:

Our attention was initially focused on cyanogen $(NC)_2$, which we had observed as a product generated by the photooxidation of NC^- by catalytic copper salts. We wondered if displacement of NC^- from $(NC)_2$[1,2] by imidazole might lead to NCI.[3] Whilst stoichiometric copper(II) salts are unlikely to have been widely available on primitive Earth, we used $CuCl_2$ to generate $(NC)_2$ preparatively in order to examine the reaction of imidazole with $(NC)_2$ by NMR spectroscopy (Figs. S1-S3). Unfortunately, the reaction of imidazole with $(NC)_2$ did not result in the formation of NCI nor its imidazole adduct IDI but instead gave a compound that we tentatively assign as cyanoformimidoylimidazole (CFI, Figure 1A and Fig. S2-S9). Copper(I) is thought to coordinate to $(NC)_2$ and to eliminate the possibility of copper-$(NC)_2$ coordination interfering with the reaction described above we generated $(NC)_2$ using potassium ferricyanide as a cyanide oxidant, but observed no change in its (uncompleted) reactivity with imidazole.[4] This could be explained by examining the initial imidazole $(NC)_2$ adduct, CFI, and the relative pK_{aH}'s of the two possible leaving groups, which indicate imidazole is a better leaving group (pK_{aH} 6.95) than NC^- (pK_{aH} 9.21) at circumneutral pH. NCI in the presence of imidazole will readily form IDI and is thereby more hydrolytically stable in the presence of imidazole than alone (Figs. S4-S7).[5–7] Interestingly, addition of NCI to imidazole and cyanide at pH 7 results in rapid conversion to IDI and $(NC)_2$, the latter of which undergoes hydrolysis to cyanoformamide thence cyanate (Fig. S9). Whilst NCI is in equilibrium with IDI, it does not appear to be in equilibrium with CFI under these conditions (Fig. 1 & Fig. S9). Attempts at using 2-aminoimidazole (2AI), with its higher pK_{aH} of ~ 9.7, in conjunction with $(NC)_2$ were also unsuccessful, which we ascribe to rapid competitive hydrolysis of $(NC)_2$ at elevated pH. Thus, we considered what oxidised form of NC^- would liberate a better leaving group than NC^- upon reaction with imidazole.

Synthesis and isolation of cyanogen

To a 3-necked round-bottomed flask containing a solution of $CuCl_2.2H_2O$ (50.0 mmol, 8.53 g) in H_2O (10 mL) and a stirrer bar was connected an N_2 inlet (used as a carrier gas), a gas outlet and a rubber septum in the final neck. The gases flowing through the outlet were sparged through an aqueous solution (25 mL) of $Ag(NO_3)$ (25.0 mmol, 4.25 g) with 0.1 M nitric acid (5 drops) added, then passed through a drying column containing $CaCl_2$ and finally through a

-78 °C trap. The effluent gases were scrubbed through sodium hydroxide/bleach solution. NaCN (1.04 eq. 51.8 mmol, 2.54 g) was dissolved in a minimum amount of H_2O (*ca.* 5 mL). This solution was added slowly *via* syringe through the rubber septum. There was an exothermic reaction, and gas could be seen evolving (the rate of cyanide addition was slowed if the reaction became too aggressive/warm). At the end of the cyanide addition, sufficient cyanogen (colourless solid in the final trap) was deemed to have been collected and so any dissolved cyanogen remaining in the copper solution and in the $Ag(NO_3)$ solution was sparged directly through the bleach solution (although this could have been collected if desired). The trap was stoppered with a new septum and an empty balloon attached via a needle. The flask was removed from the acetone/CO_2(s) bath and (1.18 g, 1.50 mL) of HPLC grade MeCN added to dissolve the cyanogen crystals. The flask was placed in an ice/water bath. A small flask was stoppered with a new septum and pre-weighed, then an empty balloon was attached via needle. The cyanogen solution was syringed into the small flask, balloon removed and weighed. It was determined that cyanogen (2.31 mmol, 120 mg, 10%) had been recovered, which gave a solution of *ca.* 1.5 M cyanogen in MeCN.

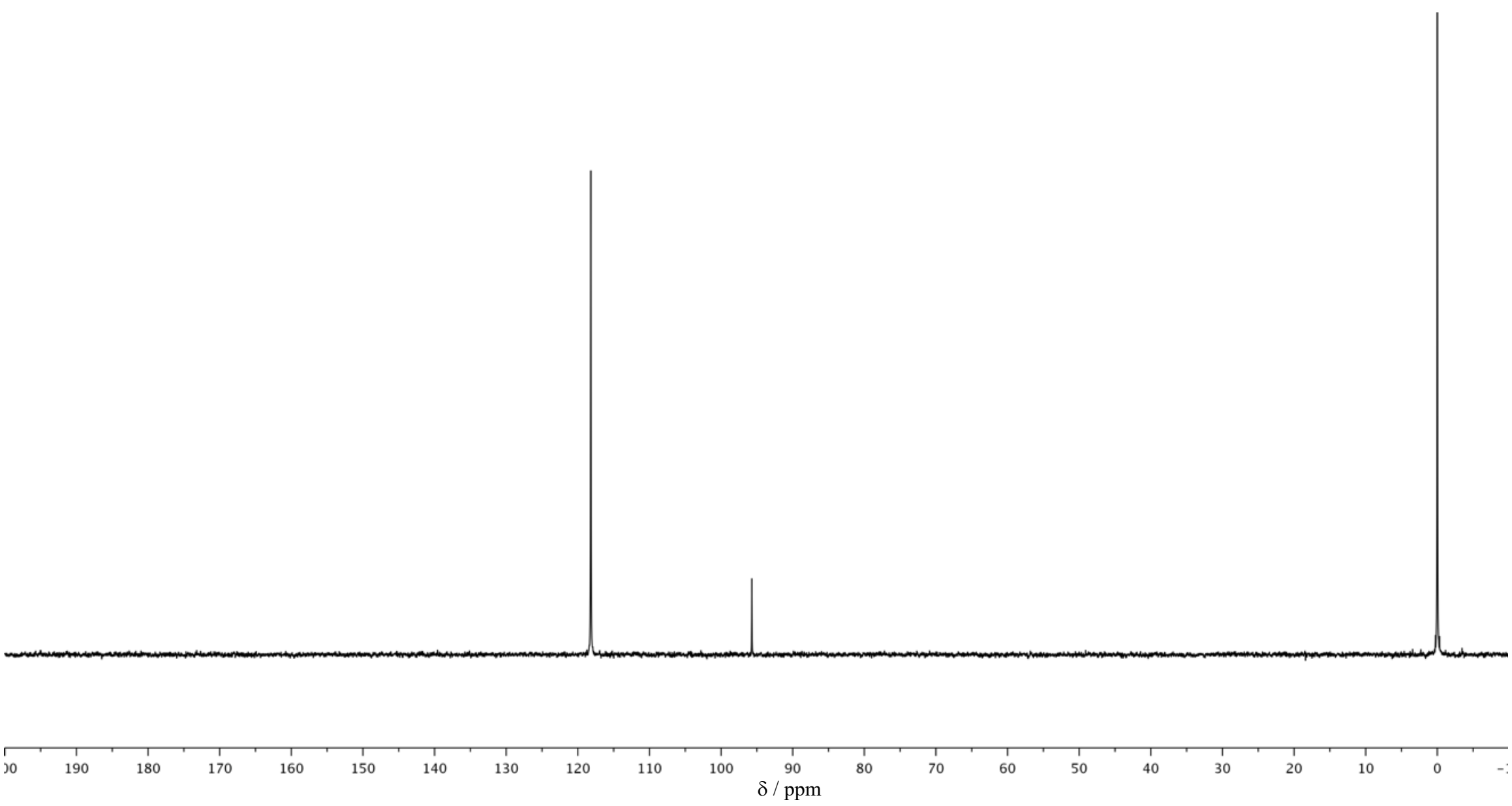

Fig. S1 ^{13}C-NMR Spectrum of the MeCN solution of cyanogen in D_2O: δ 95.7 ppm (peaks at 118.2 and 0.0 ppm are from MeCN).

Attempt to form N-cyanoimidazole (NCI) or N,N-iminodiimidazole (IDI) from cyanogen and imidazole

Imidazole (0.400 mmol, 27 mg) was dissolved in H_2O/D_2O (300 μL/100 μL) and the pH was adjusted to 7.8. Cyanogen solution (1.5 M in MeCN, 200 μL) added and the volume made up to 1 mL with H_2O, sealed and mixed. The reaction was monitored by NMR spectroscopy.

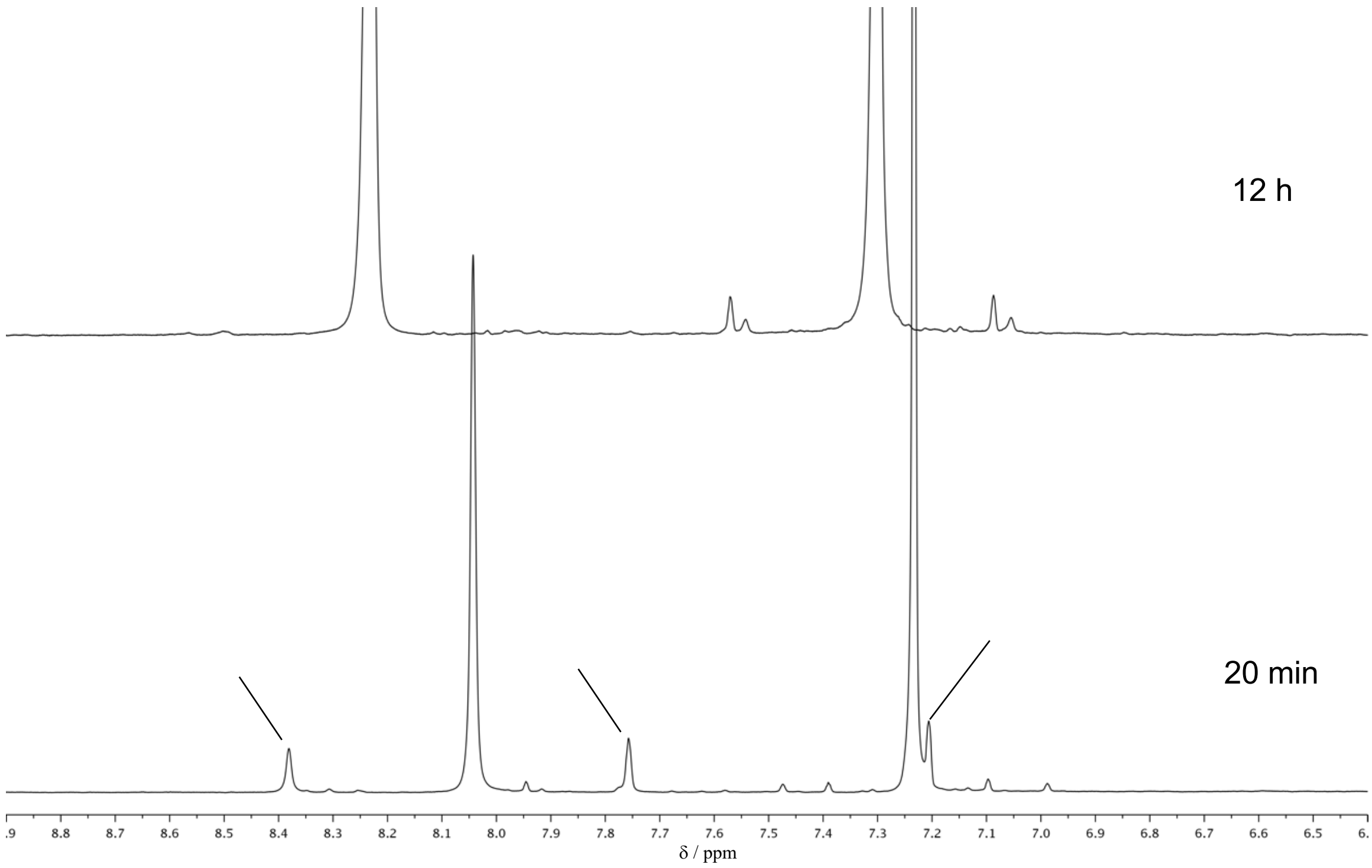

Fig. S2 ^{1}H-NMR Spectra of imidazole in the presence of cyanogen. A new unsymmetrical imidazole is formed at the beginning of the reaction which hydrolyses with time. Initially it was assumed to be NCI or IDI, but further studies showed that this new imidazole species was most likely *N*-cyanoformimidoylimidazole (CFI, *vide infra*).

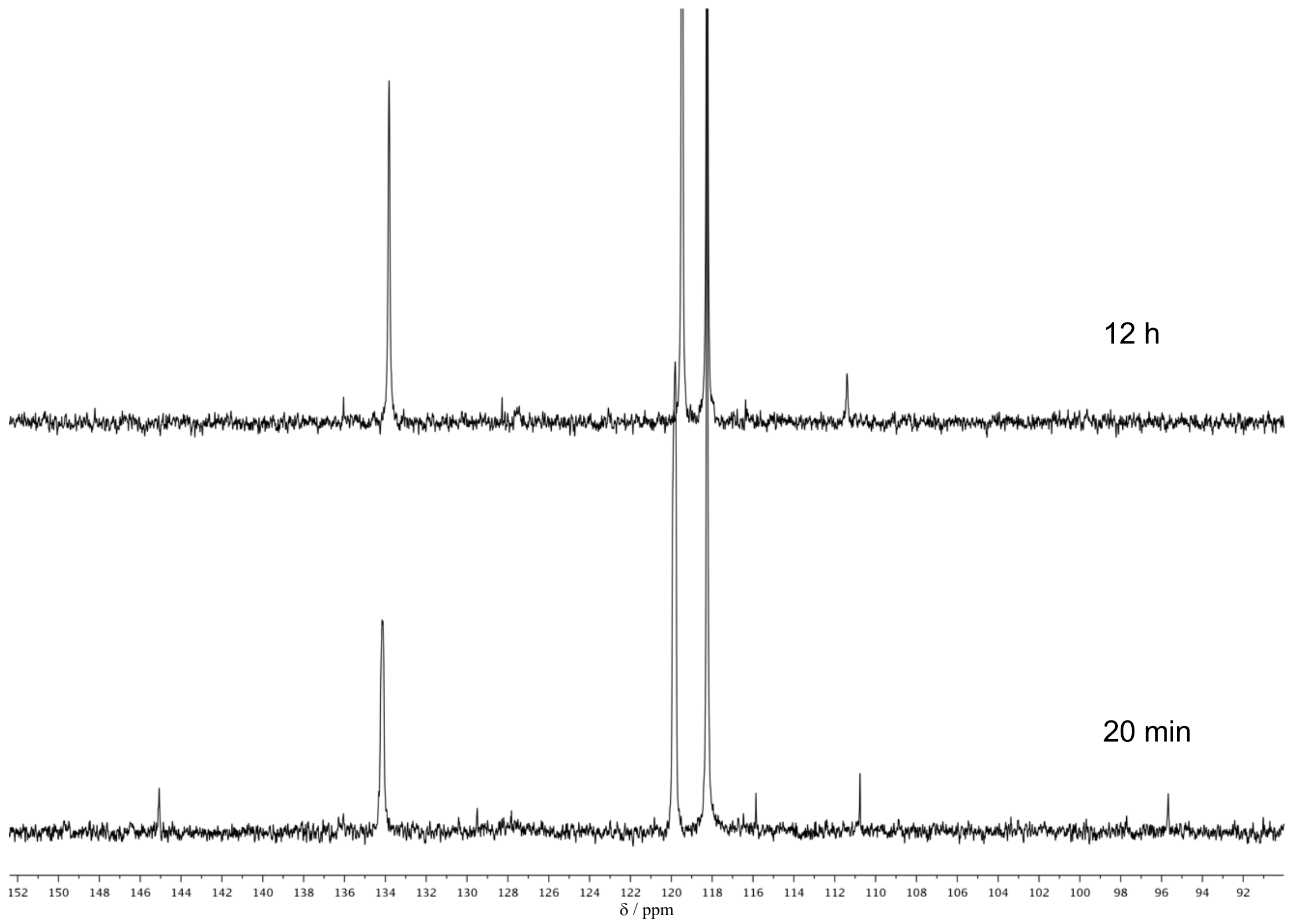

Fig. S3 ^{13}C-NMR Spectra of imidazole in the presence of cyanogen. After 20 min the new imidazole adduct was not present in sufficient amounts to give definitive signals. The peaks at 145.1 and 110.8 ppm correspond to cyanoformamide, which results from hydrolysis of cyanogen, the additional signal at 118 ppm is due MeCN from the cyanogen solution.

Imidazole (0.280 mmol, 19 mg) was dissolved in D_2O/H_2O (0.1/0.35 mL) and the pH was adjusted to 7.7 with HCl. NCI (0.075 mmol, 7 mg) was added, the volume made up to 0.55 mL and the reaction monitored by NMR spectroscopy.

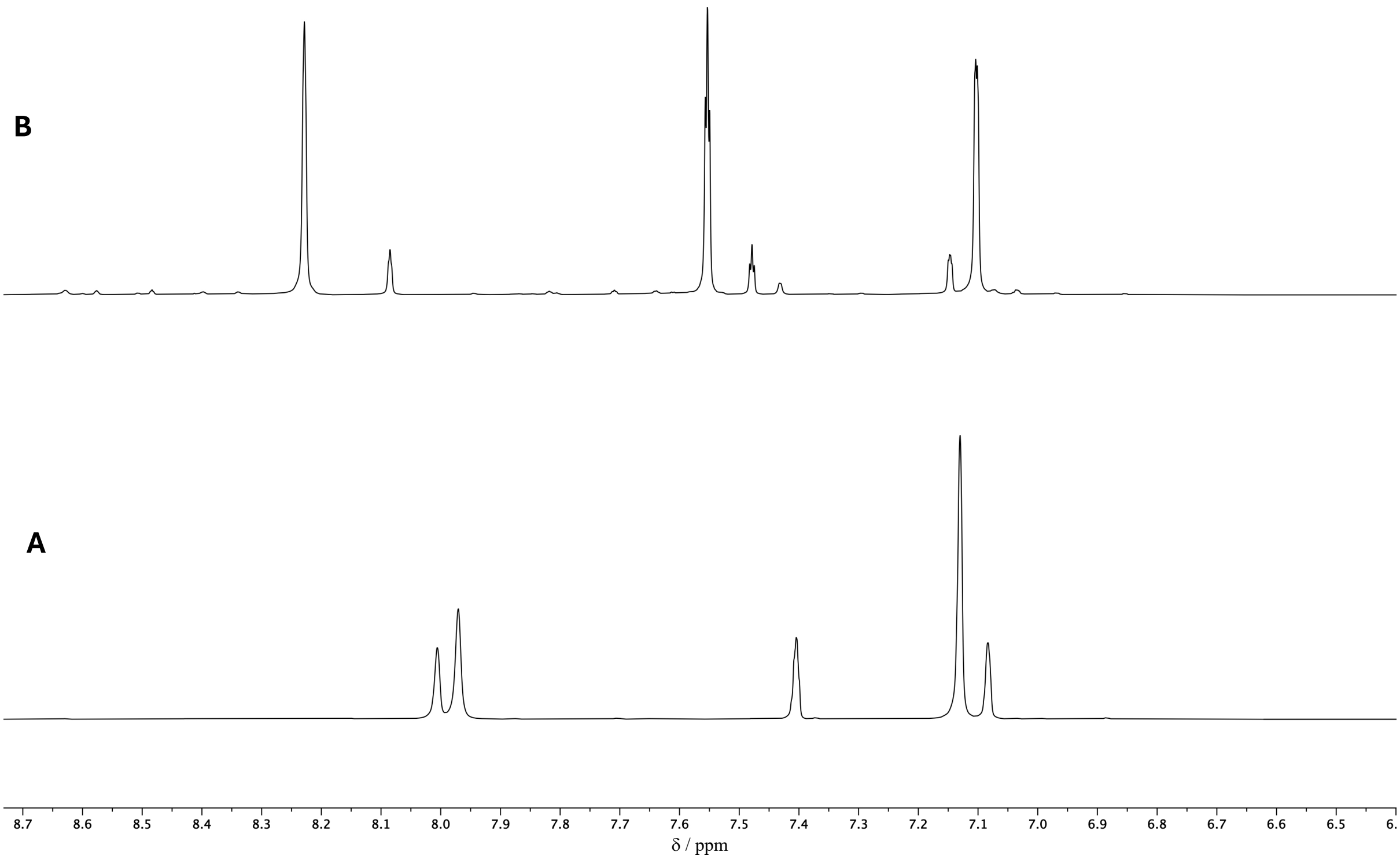

Fig. S4 ^{1}H-NMR Spectra showing the formation of IDI. Spectrum A – ^{1}H-NMR spectrum of the crude reaction mixture after 30 min. The peaks corresponding to imidazole are seen at 7.96 (s, 1H) and 7.13 (s, 2H) ppm. The remaining peaks integrate in a ratio of 1:1:1 indicating the presence of an unsymmetrical imidazole; Spectrum B – ^{1}H-NMR spectrum of a solution of NCI (48 mg, 0.516 mmol) dissolved in D_2O (0.55 mL) after 10 mins (centrifuged before spectrum was collected). A small amount of IDI can be seen (8.09, 7.48 and 7.15 ppm) which results from partial hydrolysis of NCI liberating imidazole, which can then add to further NCI. The slightly altered chemical shifts are due to different pH of the solutions. That the major compound in Spectrum B was NCI was confirmed by comparing ^{13}C-NMR spectra (Fig. S5).

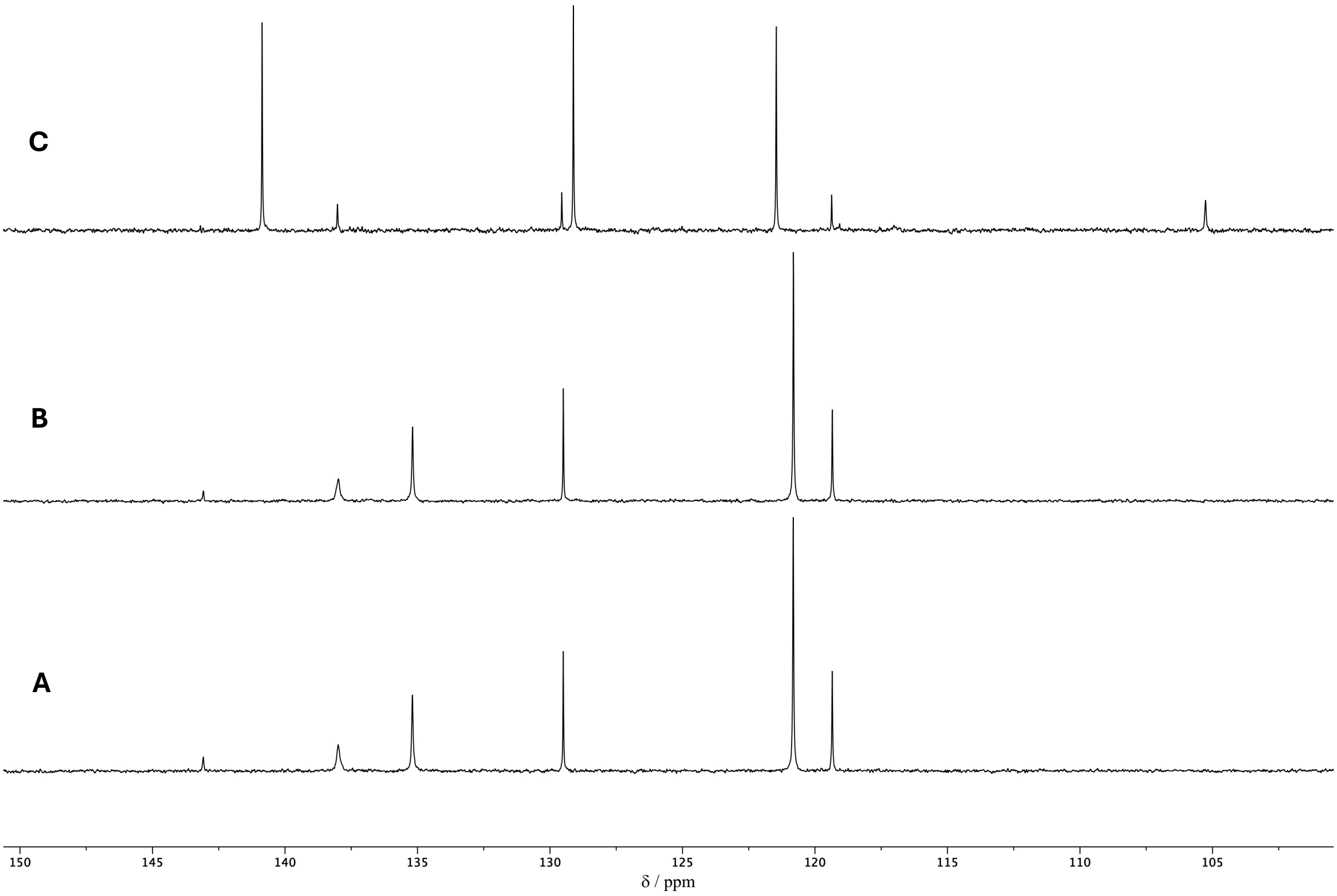

Fig. S5 ^{13}C-NMR Spectrum of the reaction between NCI and imidazole. Spectrum A – ^{13}C-NMR spectrum of the reaction described in Fig. S4, Spectrum A, after 20 min. The imidazole, present as buffer, can be seen at 135.2 and 120.8 ppm. Unidentified signals at 142.8, 137.8, 129.4 and 119.2 ppm were due to the new, unsymmetrical imidazole, and although the signal at 119.2 ppm would appear to be a nitrile carbon, the nitrile carbon of NCI has an upfield resonance of 105.3 ppm (see Spectrum C). The downfield signal at 142.8 ppm was determined by HSQC NMR spectroscopy to be a quaternary centre (see Fig. S6); Spectrum B – As Spectrum A after 1.25 h. No change is observed at this timepoint, indicating the stability of IDI in imidazole buffer; Spectrum C – ^{13}C-NMR spectrum of NCI in D_2O after 20 min (as described in Fig. S4, Spectrum B); NCI d 140.8, 129.1, 121.5 and 105.3 ppm. The signals corresponding to low levels of IDI can also be seen in Spectrum B.

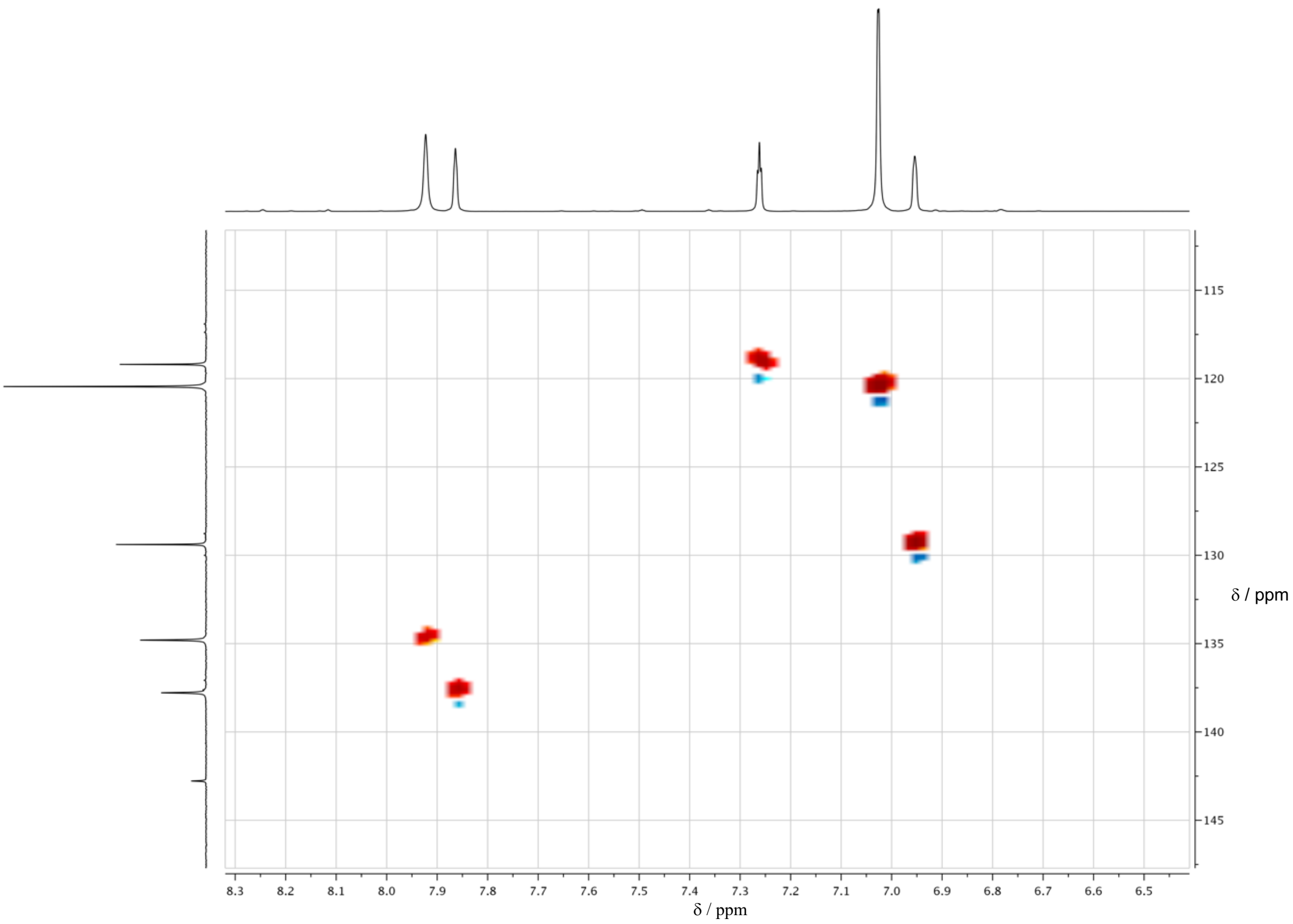

Fig. S6 HSQC NMR Spectrum of IDI in imidazole buffer. A sample of IDI in imidazole buffer was prepared as described above and subjected to HSQC NMR spectroscopy. Kanaya and Yanagawa assigned the most upfield carbon signal (~ 119 ppm) as the imino carbon of IDI, but the 2-D spectrum above clearly demonstrates that the downfield signal (142.8 ppm) is a quaternary centre that can only be attributed to the imino carbon of IDI.[8]

Investigating the pH dependence of the equilibrium between NCI and IDI

Imidazole (2.0 mg, 0.025 mmol) and NCI (2.0 mg, 0.025 mmol) were dissolved in in D_2O/H_2O (50 μL/450 μL), the pH was adjusted to 6.4 with HCl and a ^{1}H NMR spectrum of the mixture was recorded. The pH of the reaction mixture was then adjusted to 3.2 using HCl and a ^{1}H NMR spectrum was recorded again.

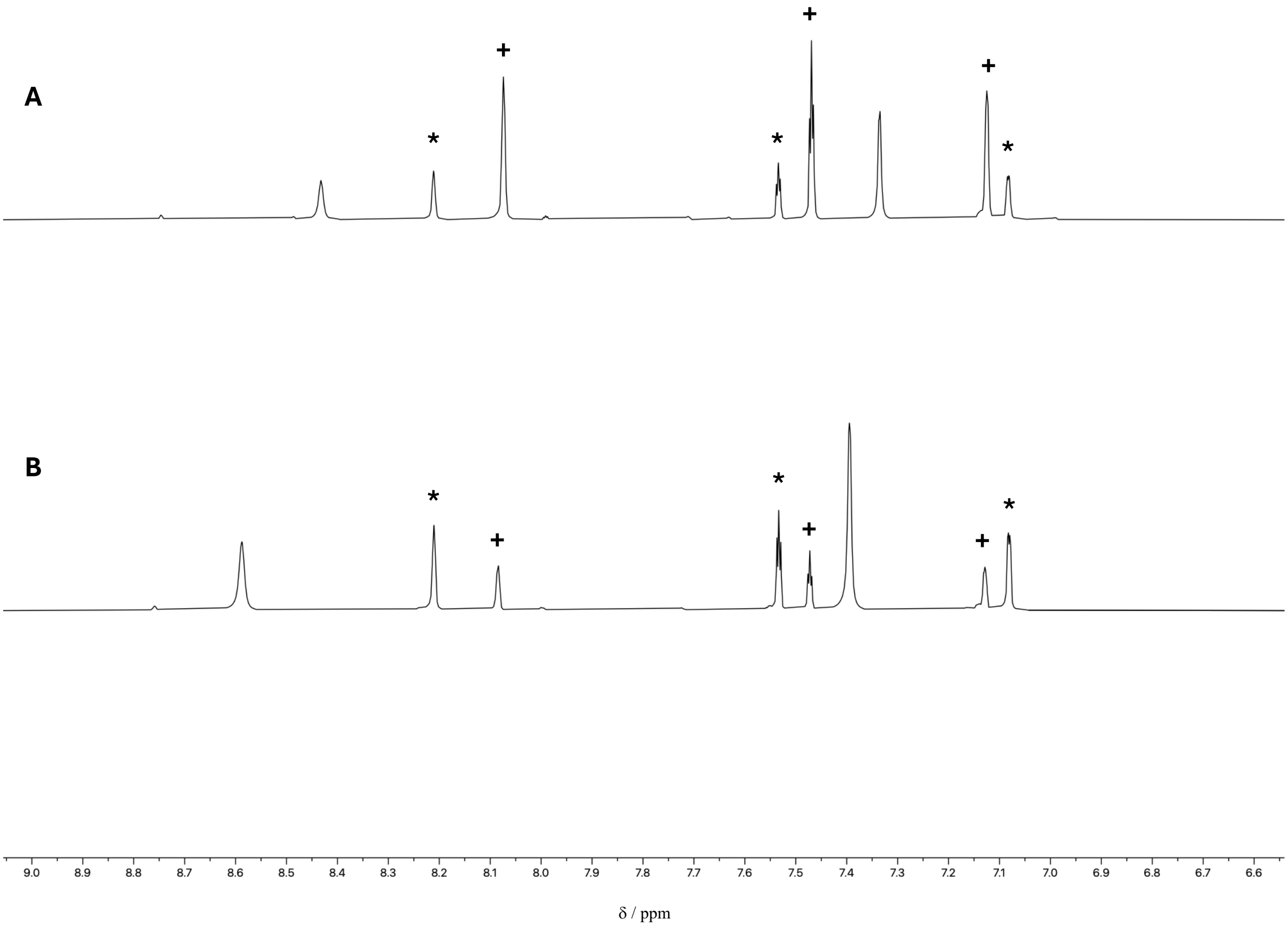

Fig. S7 Spectrum A – ^{1}H-NMR spectrum of the reaction mixture of imidazole (50 mM) and NCI (50 mM) at pH 6.4. Spectrum B – ^{1}H-NMR spectrum of the same reaction mixture of imidazole and NCI after adjusting the pH to 3.2. The imidazole peaks shift slightly due to the pH change. There is also a clear shift in the equilibrium position from IDI to NCI as the pH is lowered. Peaks belonging to IDI are labelled with **+** and peaks belonging to NCI are labelled with *****.

IDI spiking experiment

Satisfied with the synthesis and characterisation of IDI, we then repeated the reaction of imidazole and cyanogen (as described in Fig S2) and then spiked in a sample of IDI solution, as prepared above.

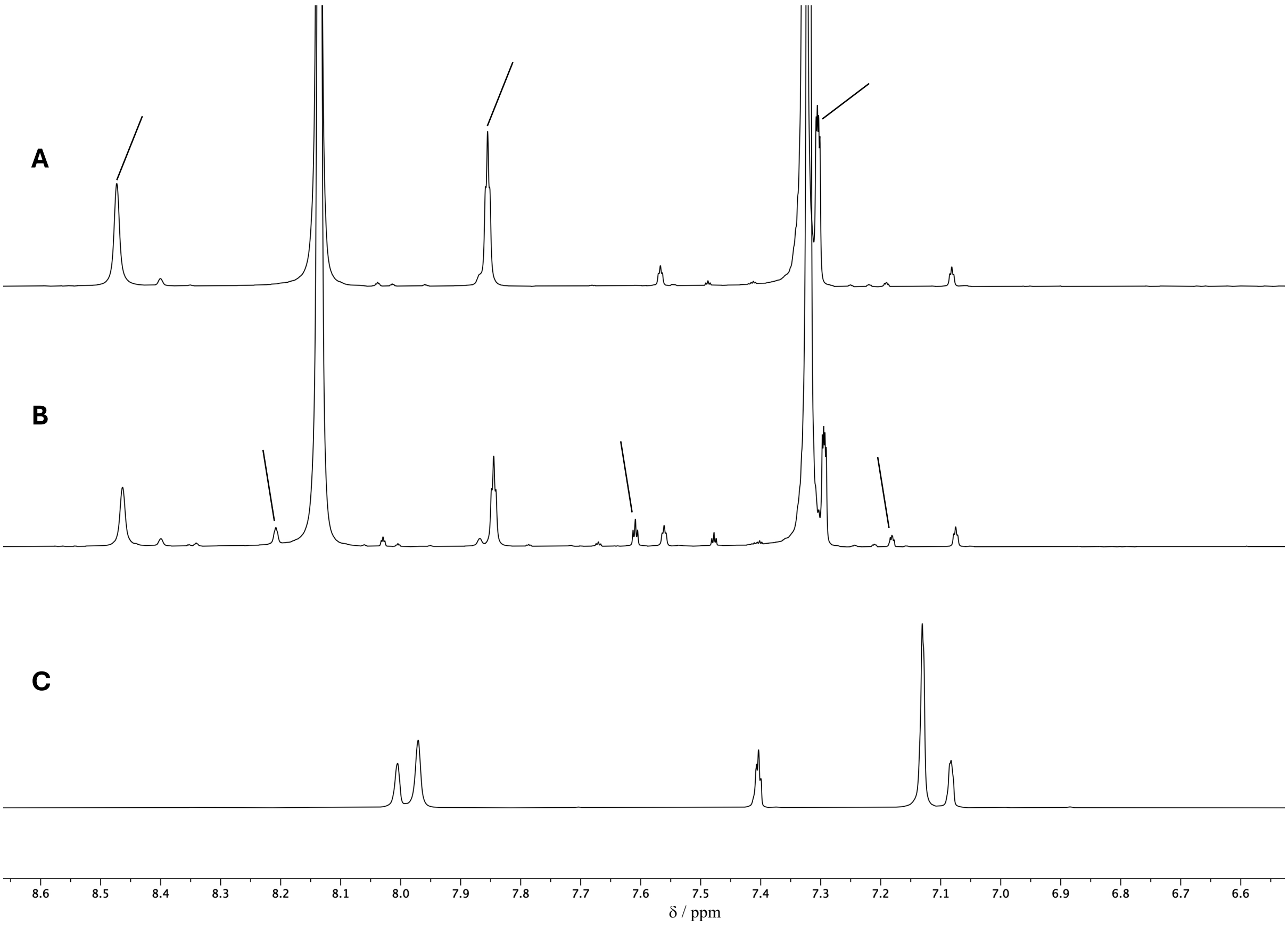

Fig. S8 Spiking experiment with IDI. Spectrum A – ^{1}H-NMR spectrum of the reaction of imidazole with cyanogen (as described for Fig. S2), the new unsymmetrical imidazole is indicated; Spectrum B – As Spectrum A, with the addition of a small amount of IDI solution (as prepared for Fig. S4). It can clearly be seen that the new species in Spectrum A is not IDI, and we therefore ascribe it to CFI; Spectrum C – ^{1}H-NMR spectrum of the IDI solution used for spiking in Spectrum B (chemical shifts vary due to differences in pH. Signals at 7.97 and 7.12 ppm are due to imidazole).

Investigation of the equilibrium between cyanogen, imidazole, NCI, IDI and cyanide

$K^{13}CN$ (0.100 mmol, 7 mg) and imidazole (0.100 mmol, 7 mg) were dissolved in D_2O/H_2O (0.1/0.6 mL) with and the pH adjusted to 7 with HCl. The volume was increased to 1 mL with H_2O, then NCI (0.100 mmol, 9 mg) was added. NMR Spectra were then recorded.

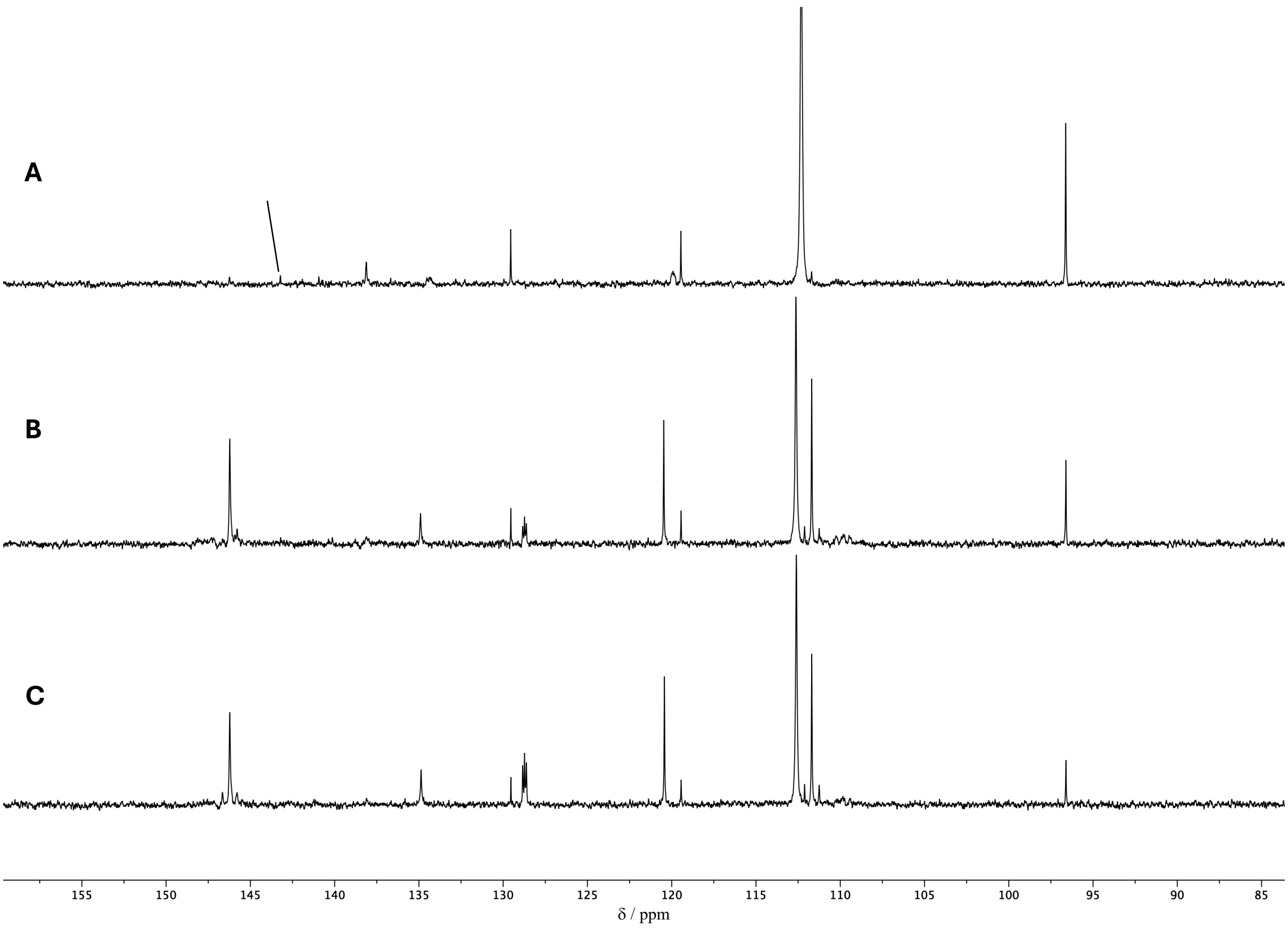

Fig. S9 ^{13}C-NMR Spectra of NCI in the presence of imidazole and $H^{13}CN$. Spectrum A – ^{13}C-NMR Spectrum of the reaction after 15 min. Intense signal at 112.3 ppm is due to $H^{13}CN$, broad signals at 134.4 and 120.0 ppm are due to imidazole and signals at 119.5, 129.5, 138.1 and 143.3 ppm (indicated) are due to IDI. The singlet at 96.6 ppm is due to cyanogen which has clearly incorporated some ^{13}C-labelled cyanide; Spectrum B – ^{13}C-NMR Spectrum of the reaction after 1.5 h. The intensity of the cyanogen peak has decreased slightly, and its hydrolysis products cyanate (triplet, 128.8 ppm) and cyanoformamide (singlets, 146.2 and 111.7ppm) can now be seen. Interestingly, the cyanoformamide signals are also present at a lower intensity as doublets *(J* = 87.5, more easily seen in Spectrum C). This shows that singly ^{13}C-labelled cyanogen ($NC\text{-}^{13}CN$) is produced by reaction of ^{13}C-labelled cyanide with NCI, and the cyano groups of cyanogen can then undergo reaction and exchange with cyanide in solution, leading to some doubly ^{13}C-labelled cyanogen ($N^{13}C\text{-}^{13}CN$). The peak corresponding to the imino carbon of IDI (143 ppm) does not increase in intensity during the course of the reaction, which indicates there is no equilibrium between cyanogen, imidazole and NCI (and thence IDI). This correlates with the spiking experiment in Fig. S7, where IDI could not be detected by routine ^{1}H-NMR spectroscopy after reaction of cyanogen with imidazole. As the reaction proceeds cyanate and cyanoformamide can be seen to accumulate, and their integration is much larger than that which would be derived from the amount of cyanogen produced in Spectrum A. Additionally, cyanogen continues to persist throughout the time course whilst the hydrolysis products accumulate, and consequently cyanogen must be replenished as the reaction progresses. Thus, IDI must be in equilibrium with NCI which can then react unidirectionally to give cyanogen.

Reactions involving thiocyanogen $(NCS)_2$

Discussion of reactions involving thiocyanogen

Keefe and Miller reported an underexplored example of phosphate activation where an aqueous solution of inorganic phosphate (5 mM), hydrogen peroxide (H_2O_2) (5-30 mM) and thiocyanate (NCS^-) (5 mM) gave yields of pyrophosphate of up to 26% at room temperature.[9] This piqued our interest as the cyanosulfidic scenario can account for the formation of NCS^-, which is readily produced alongside HCN reduction products by simple UV irradiation of NC^- and hydrosulfide.[10] While there is still debate on the prebiotic plausibility of H_2O_2, we thought it worth further exploring this observation from the literature.[11] Keefe and Miller postulated that thiocyanogen ($(NCS)_2$) or hypothiocyanous acid (HOSCN) might be the activating agent responsible for the conversion of phosphate to pyrophosphate. It has been reported that imidazole emerges unscathed after exposure to thiocyanogen in an attempt at effecting its c-thiocyanation.[12] It struck us that these observations did not preclude the transient formation of NCI which, had it indeed been formed, would have been hydrolised back to imidazole by the work-up procedure employed. Intriguingly, N-cyanation of imidazole by thiocyanogen would involve loss of an excellent leaving group ($NCSS^-$). Using ^{13}C-NMR spectroscopy in conjunction with ^{13}C-labelled reagents we set out to elucidate the steps involved in the activation reaction reported by Keefe and Miller and to establish if such chemistry could be used to generate NCI.

We started our investigation by treating ^{13}C-labelled KSCN (100 mM) with H_2O_2 (200 mM) and following the reaction by ^{13}C NMR spectroscopy (Fig. 2A). After 4 hours a 70% conversion and three major products were observed, and their chemical shifts matched reports from the literature for: $(NCS)_2$ (48%)[13], $NCSO^-$ (11%)[14], and HCN (13%). Cyanide is produced alongside sulfate as the decomposition product of sulfocyanide ($NCSO_3^-$), formed upon further oxidation of $NCSO^-$ by H_2O_2. The production of sulfate also accounts for the significant drop in pH from 7 to 2.5. Small amounts of cyanamide and urea can be observed if the reaction is monitored for extended periods of time (Fig. S10C). Upon reacting this mixture with 3'-AMP formation of 2',3'-cAMP was observed (Fig. S16). In order to determine the reactive species, a sample containing $NCSO^-$ as the only product was prepared by raising the pH of the $(NCS)_2$ reaction mixture above 5, thus resulting in the complete hydrolysis of any $(NCS)_2$ to NCS^- and $NCSO^-$. After addition of 3'-AMP, no reaction was observed. This suggested that $(NCS)_2$ is the activating agent, so a sample of pure $(NCS)_2$ was prepared by adding a trace amount of Fe^{2+} to

the NCS^-/H_2O_2 reaction.[15] Subsequent addition of 3'-AMP resulted in the rapid conversion to 2',3'-cAMP, confirming $(NCS)_2$ as the phosphate activating agent. Reaction with 5'-AMP was less successful, only ever giving a low steady state concentration of what we presumed was the hypothiocyanite phosphate ester (< 5%) that quickly underwent hydrolysis (Fig. S17). While not encouraging, we still attempted a nicked duplex ligation with $(NCS)_2$ as the sole activating agent. Disappointingly, but perhaps not surprisingly, only starting material was observed.

Next, we subjected ^{13}C-labelled $(NCS)_2$, prepared as an unbuffered solution, to addition of imidazole, which raised the solution pH to 7. ^{13}C NMR showed a peak at δ 143 ppm indicating the formation of IDI via NCI (Fig. 2B). If the reaction pH was adjusted to pH 6, both NCI and IDI peaks could be observed in the ^{1}H NMR spectrum. When 3'-AMP (25 mM) was added to a solution of NCI/IDI (30 mM) prepared from $(NCS)_2$, steady formation of 2',3'-cAMP could be observed with a maximum yield of 18% reached after 32 h (Fig. S19). A similar reaction with commercial NCI and no other additives gave the cyclic phosphate in 58% yield after 24 h (Fig. S20). We then turned to the formation of the phosphorimidazolide from 5'-AMP using NCI, with both commercial NCI and our prebiotically plausible mixture containing IDI/NCI. Unfortunately, the reaction of 5'-AMP (25 mM) at NCI/IDI concentrations of around 50 mM and pH 6 gave only low yields of ImpA (<10%). We then wondered if IDI may be a better reagent for phosphorimidazolide formation and so increased the pH of the reaction to 7 whilst utilising imidazole as a buffer. At a starting concentration of 100 mM NCI, a 31% yield of ImpA was achieved (Figs. S22-S24). Whilst this yield was not particularly high, phosphorimidazolides derived from imidazole are not true indicators for RNA ligation potential,[16] and so we next investigated RNA ligation in a nicked duplex using NCI as the initial NCI-5'-phosphate adduct has been shown to be effective for RNA ligation.[17]

Reaction of KS^{13}CN with hydrogen peroxide (H_2O_2)

KS^{13}CN (10 mg, 0.10 mmol) was dissolved in 10% D_2O in H_2O (0.5 mL), then 30% H_2O_2 solution (21 µL, 0.20 mmol) was added, and the reaction was monitored by ^{13}C NMR.

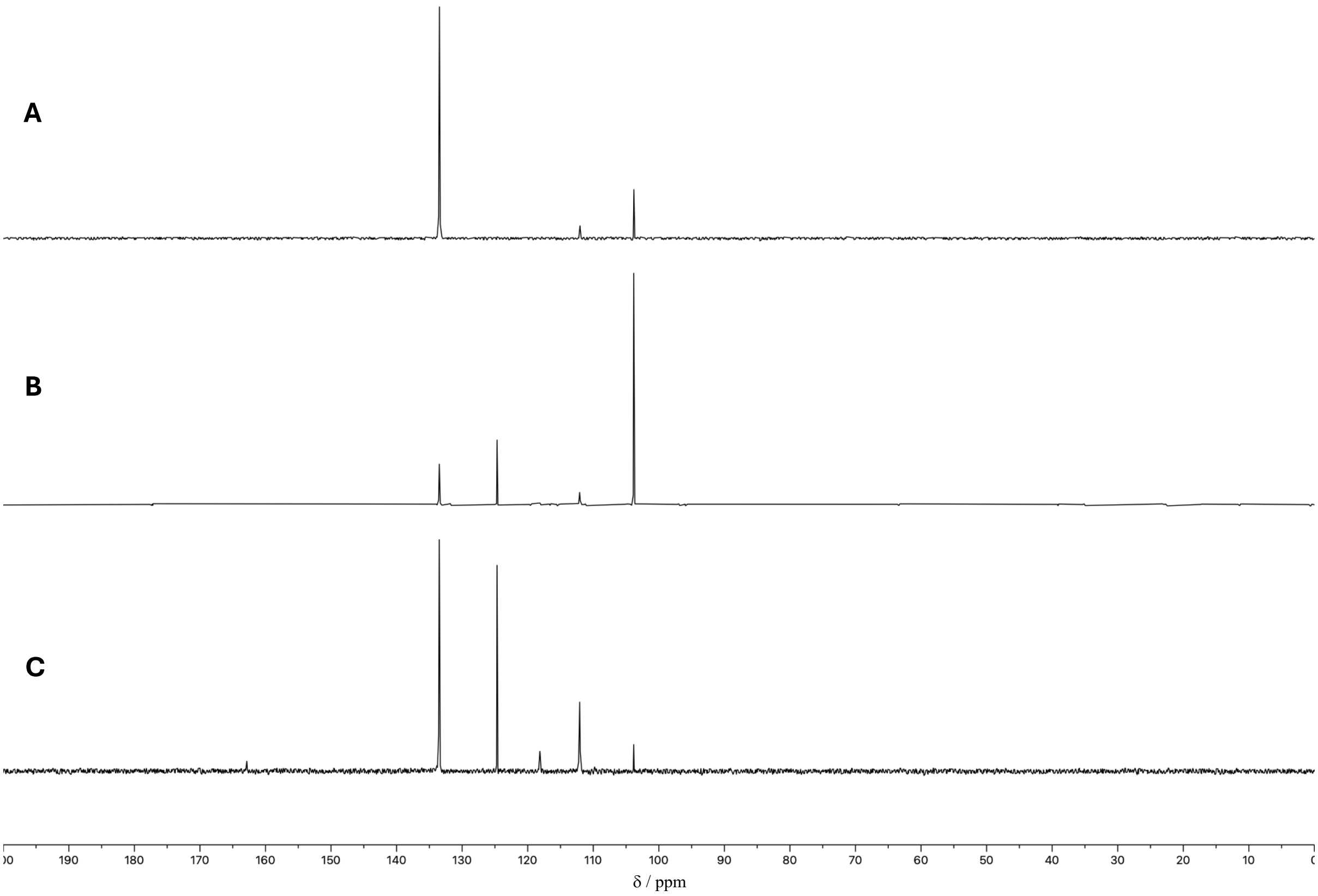

Fig. S10 ^{13}C-NMR spectra of the reaction between KS^{13}CN (200 mM) and H_2O_2 (400 mM) in 10% D_2O in H_2O. Spectrum A – Initial ^{13}C-NMR spectrum recorded after 0 h; δ 104 ppm = $(NCS)_2$,[3] δ 112 ppm = HCN, and δ 133 ppm = NCS^-; Spectrum B – ^{13}C-NMR Spectrum recorded after 8 h; δ 104 ppm = $(NCS)_2$, δ 112 ppm = HCN, δ 124 ppm = $NCSO^-$,[14] and δ 133 ppm = NCS^-; Spectrum C – ^{13}C-NMR spectrum recorded after 5 d; δ 104 ppm = $(NCS)_2$, δ 112 ppm = HCN, δ 118 ppm = NH_2CN δ 124 ppm = $NCSO^-$, δ 133 ppm = NCS^-, and δ 163 ppm = $CO(NH_2)_2$.

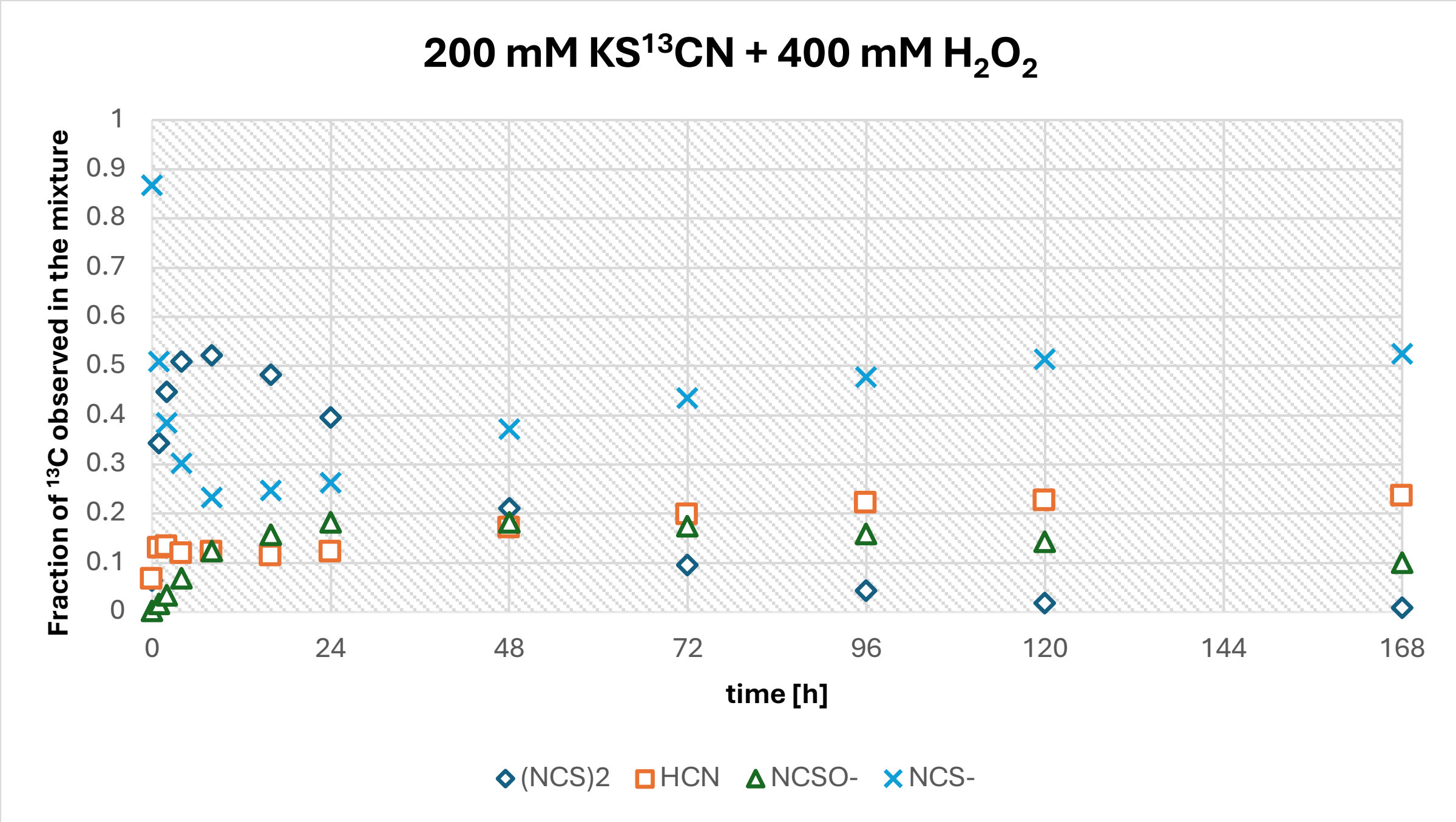

Fig. S11. Fraction of ^{13}C observed in the ^{13}C-NMR spectrum of the reaction mixture of $KS^{13}CN$ (200 mM) and H_2O_2 (400 mM) at various time points. For clarity only the four major species are included in the plot.

Optimisation of conditions for the synthesis of $(NCS)_2$

We decided to briefly investigate the ratio of NCS^- and H_2O_2 as well as their concentrations on the yield of the $(NCS)_2$ formation.

$KS^{13}CN$	H_2O_2	Max yield $(NCS)_2$	Rxn time at max yield
100 mM	200 mM	47%	8 h
100 mM	300 mM	53%	4 h
100 mM	400 mM	53%	4 h
200 mM	400 mM	54%	2 h
400 mM	800 mM	66%	2 h

Table S1 Optimisation of $(NCS)_2$ formation.

Testing the stability of $(NCS)_2$ with respect to UV irradiation

$KS^{13}CN$ (10 mg, 0.10 mmol) was dissolved in 10% D_2O in H_2O (1.0 mL), then 30% H_2O_2 solution (21 µL, 0.20 mmol) was added, and the reaction was monitored by ^{13}C NMR after 24 h. The reaction mixture was then irradiated in a quartz NMR tube at 450 nm for 1 h and the ^{13}C NMR spectrum was recorded again. No drop in $(NCS)_2$ concentration was observed.

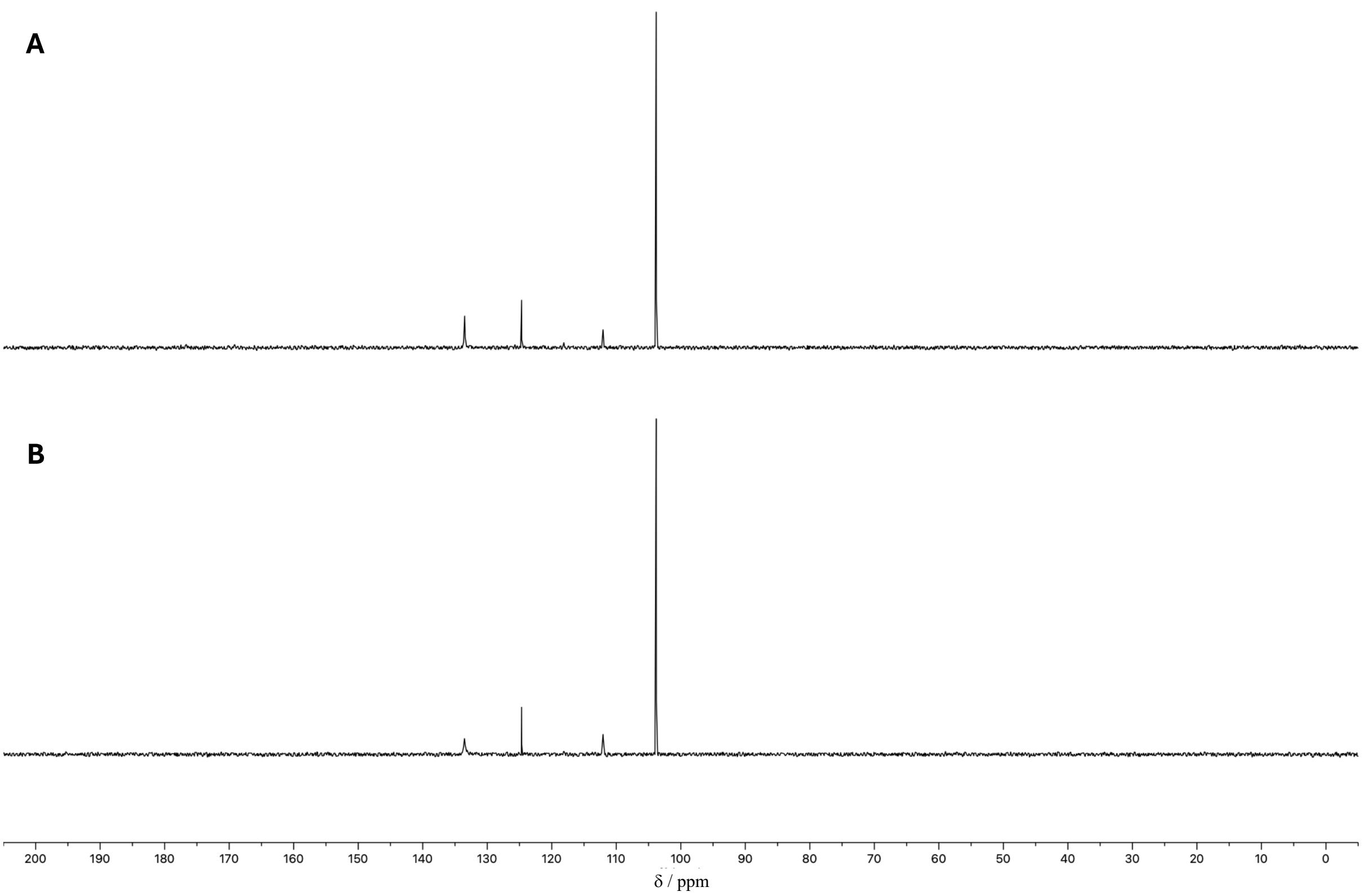

Fig. S12 Spectrum A – ^{13}C NMR spectrum of the reaction between $KS^{13}CN$ (100 mM) and H_2O_2 (200 mM) in 10% D_2O in H_2O. NMR spectrum recorded after 24 h but before any irradiation. Spectrum B – ^{13}C NMR spectrum recorded after 1 h irradiation with 450 nm; δ 104 ppm = $(NCS)_2$, δ 112 ppm = HCN, δ 124 ppm = $NCSO^-$, and δ 133 ppm = NCS^-.

$KS^{13}CN$ (10 mg, 0.10 mmol) was dissolved in 10% D_2O in H_2O (1.0 mL), then 30% H_2O_2 solution (21 µL, 0.20 mmol) was added, and the reaction was monitored by ^{13}C NMR after 16 h. The reaction mixture was then split in half and added to quartz NMR spectroscopy tubes. One sample was irradiated at 310 nm and the other half at 254 nm, for 1 h each, and the ^{13}C-NMR spectra was recorded again. All of the $(NCS)_2$ was destroyed in the sample irradiated at 254 nm. On the other hand, no drop in $(NCS)_2$ concentration was observed for irradiation at 310 nm after 1 h, and even after 4 h, no drop in concentration was detected.

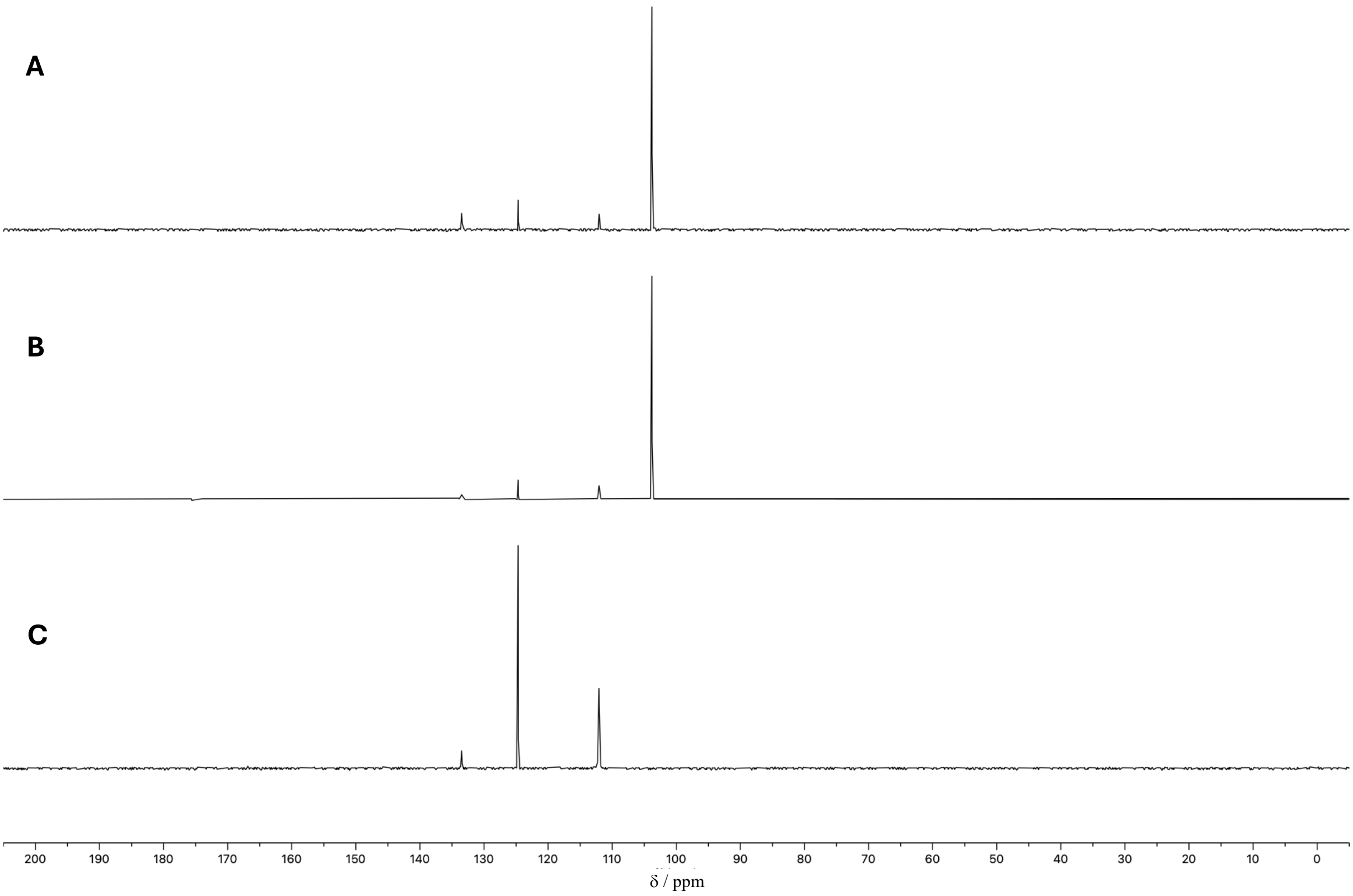

Fig. S13 Spectrum A – ^{13}C NMR spectrum of the reaction between $KS^{13}CN$ (100 mM) and H_2O_2 (200 mM) in 10% D_2O in H_2O. NMR spectrum recorded after 16 h but before any irradiation. Spectrum B – ^{13}C NMR spectrum recorded after 1 h irradiation with 310 nm; Spectrum C – ^{13}C NMR spectrum recorded after 1 h irradiation with 254 nm; δ 104 ppm = $(NCS)_2$, δ 112 ppm = HCN, δ 124 ppm = $NCSO^-$, and δ 133 ppm = NCS^-.

Reaction of $KS^{13}CN$ with H_2O_2 in the presence of Fe (II)

$KS^{13}CN$ (20 mg, 0.10 mmol) was dissolved in 10% D_2O in H_2O (0.5 mL), along with a trace amount of $FeSO_4 \cdot 7H_2O$ (~ 1 mM). Upon addition of 30% H_2O_2 solution (42 µL, 0.20 mmol) the mixture turned a blood red colour indicating the formation of $[Fe(SCN)(H_2O)_5]^{2+}$. The reaction was examined by ^{13}C NMR spectroscopy, and quantitative conversion to $(NCS)_2$ was observed.

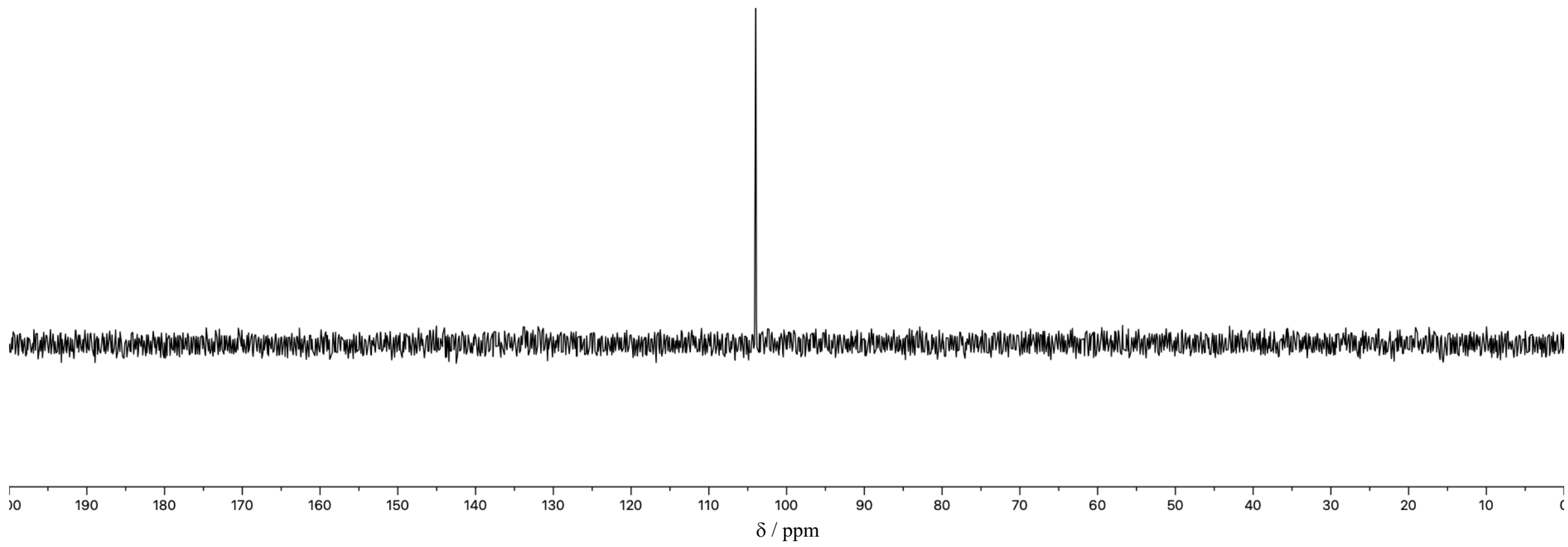

Fig. S14 ^{13}C NMR spectrum of the reaction between $KS^{13}CN$ (400 mM) and H_2O_2 (800 mM) in 10% D_2O in H_2O with 1 mM Fe(II). NMR spectrum recorded after 30 min; δ 104 ppm = $(NCS)_2$.

Treatment of $KS^{13}CN$ and H_2O_2 reaction mixture with MnO_2

$KS^{13}CN$ (12 mg, 0.125 mmol) was dissolved in 10% D_2O in H_2O (0.5 mL), then 30% H_2O_2 solution (26 µL, 0.250 mmol) was added, and the reaction was monitored by ^{13}C NMR spectroscopy. After 24 h a small amount of MnO_2 was added to the reaction mixture and after the gas evolution subsided the sample was centrifuged and the supernatant examined by ^{13}C NMR spectroscopy. We observed a substantial conversion of NCS^- and $NCSO^-$ into $(NCS)_2$.

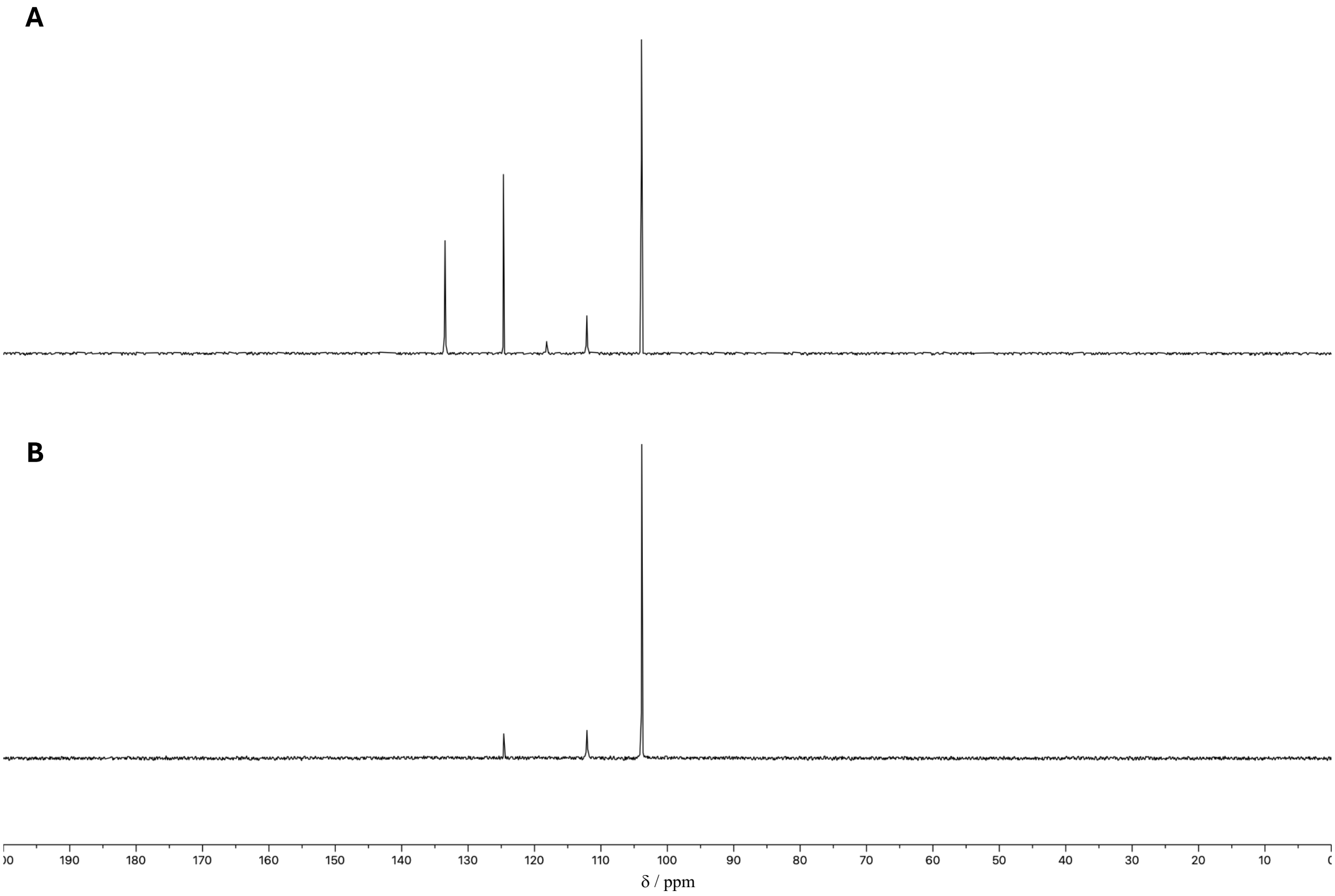

Fig. S15 Spectrum A – ^{13}C NMR spectrum of the reaction between $KS^{13}CN$ (250 mM) and H_2O_2 (500 mM) in 10% D_2O in H_2O after 24 h. Spectrum B – ^{3}C NMR spectrum of the reaction after treatment with MnO_2. δ 104 ppm = $(NCS)_2$, δ 112 ppm = HCN, δ 118 ppm = NH_2CN δ 124 ppm = $NCSO^-$, δ 133 ppm = NCS^-

Reaction of 3'-AMP with $(NCS)_2$

$KS^{13}CN$ (5.0 mg, 0.05 mmol) was dissolved in 10% D_2O in H_2O (0.5 mL), then 30% H_2O_2 solution (11 µL, 0.1 mmol) was added to the reaction. After 24 h we used ^{13}C NMR analysis to determine the concentration of $(NCS)_2$ (~ 20 mM), at which point we added 3'-AMP sodium salt (4.9 mg, 0.025 mmol) and the reaction was followed by ^{31}P NMR spectroscopy. After 24 h the yield of the cAMP was 25% as observed by ^{31}P NMR spectroscopy.

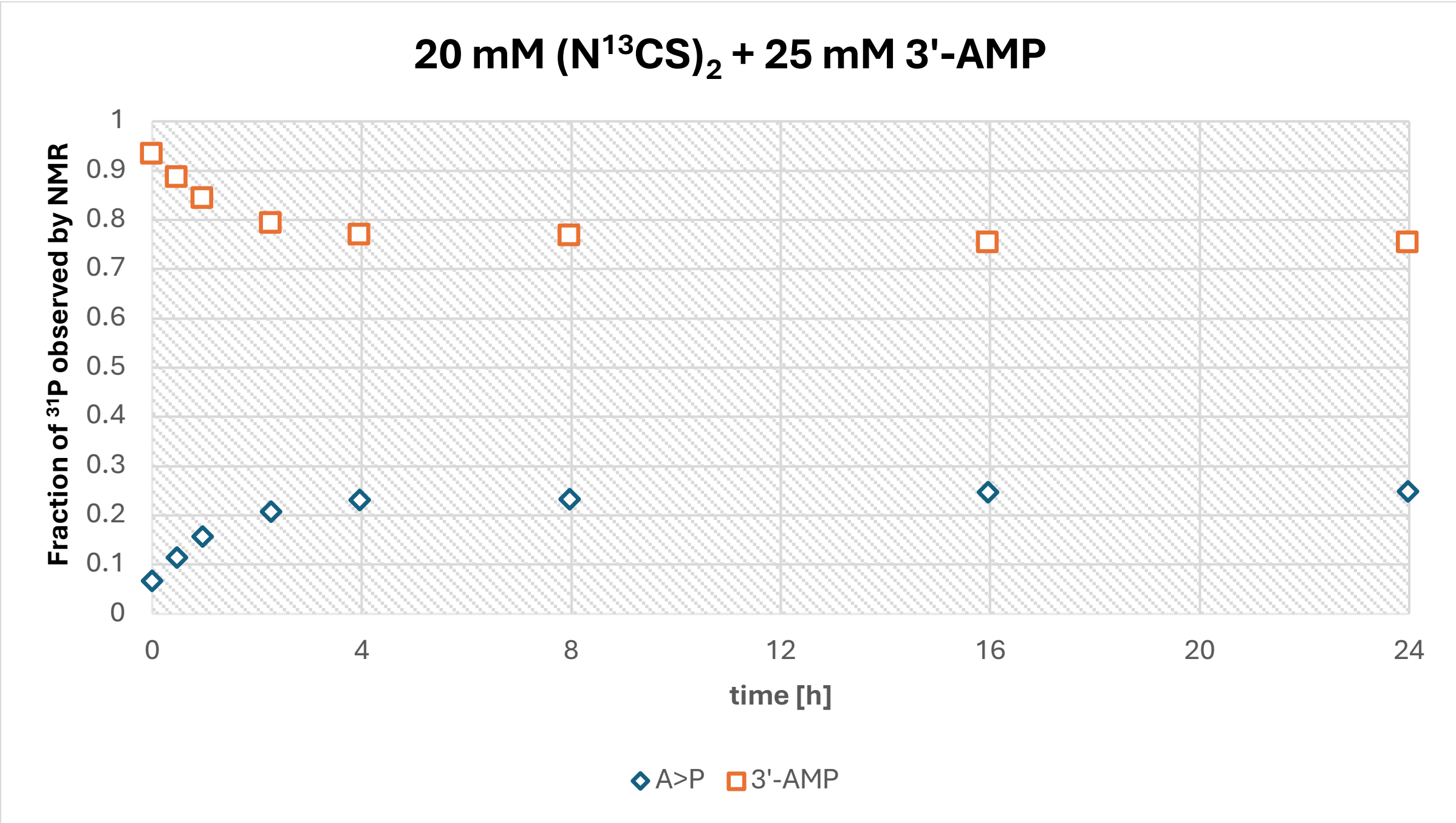

Fig. S16 Fraction of ^{31}P observed by ^{31}P NMR spectroscopy of the reaction mixture of $(S^{13}CN)_2$ (20 mM) and 3'-AMP (25 mM) at various time points.

Reaction of 5’-AMP with (NCS)₂

KS^{13}CN (20 mg, 0.10 mmol) was dissolved in 10% D_2O in H_2O (0.5 mL), and 30% H_2O_2 solution (42 µL) was added to the mixture The reaction was examined by ^{13}C NMR spectroscopy and after 4 h the concentration of (S^{13}CN)$_2$ was estimated to be 50 mM. 5’-AMP disodium salt (5.0 mg, 0.0125 mmol) was then added, and the reaction mixture was monitored by ^{31}P NMR spectroscopy. A new doublet signal at δ -7.62 ppm with J = 4.2 Hz was observed indicating the formation of a new phosphate species. Based on the ^{13}C-^{31}P coupling constant value we proposed the attack of the phosphate on the sulphur of the (NCS)$_2$. The yield of the species never exceeded 3% as observed by routine NMR spectroscopy.

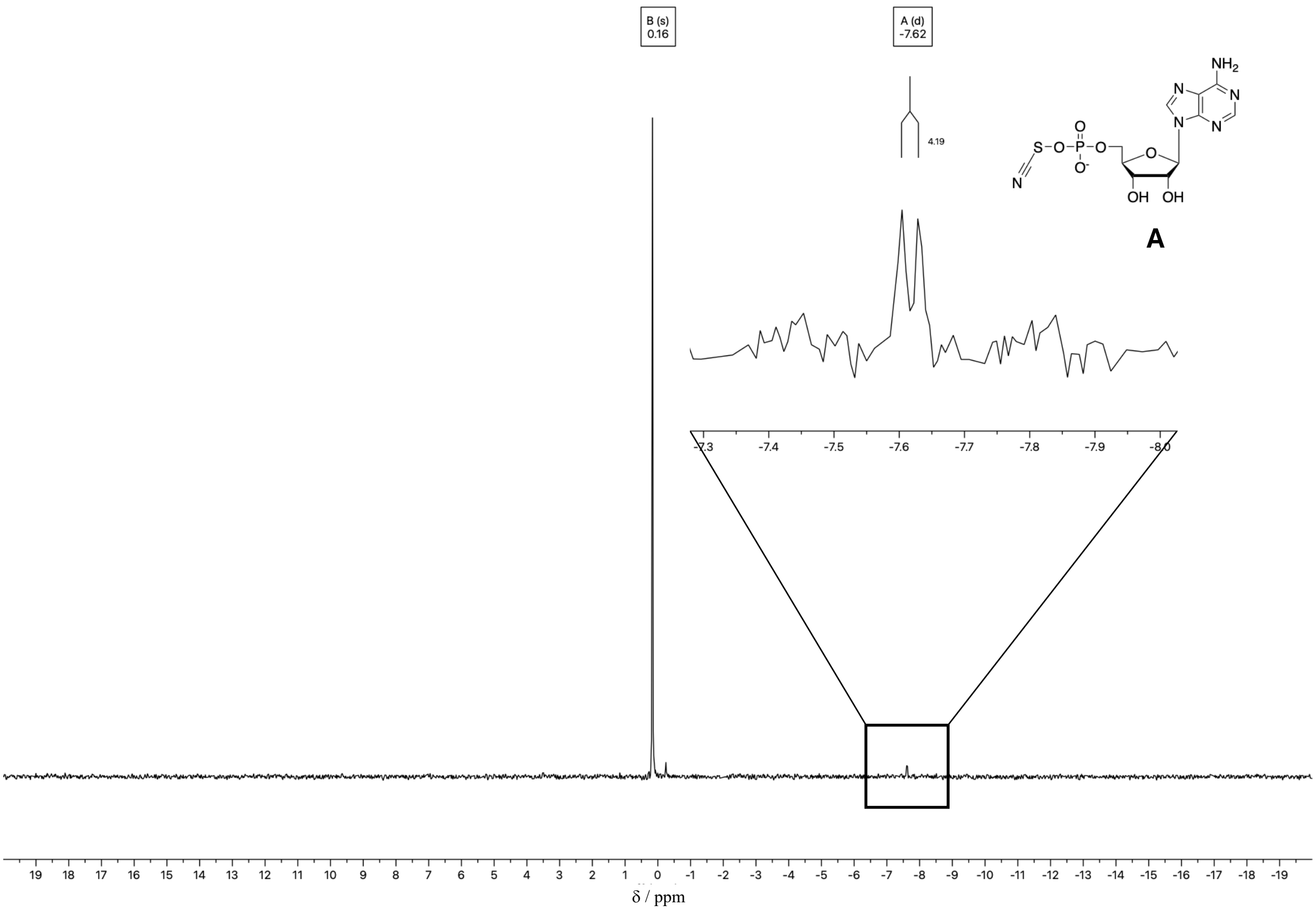

Fig. S17 ^{31}P NMR spectrum of the reaction of (N^{13}CS)$_2$ (50 mM) with 5′-AMP (25 mM) after 4 h. δ 0.16 ppm = 5′-AMP; δ -7.62 ppm = **A**.

Synthesis of NCI and IDI from $(NCS)_2$.

KS^{13}CN (10 mg, 0.10 mmol) was dissolved in 10% D_2O in H_2O (1.0 mL) along with a trace amount of $FeSO_4 \cdot 7H_2O$ (1 mM). Upon addition of 30% H_2O_2 solution (42 µL, 0.20 mmol) the mixture turned a blood red colour indicating the formation of $Fe(SCN)_3$. The reaction was examined by ^{13}C NMR spectroscopy and complete conversion to $(NCS)_2$ was observed. Then imidazole was added (7.0 mg, 0.1 mmol) and the red colour disappeared, the pH of the reaction mixture was adjusted to 7 and the reaction monitored by ^{13}C NMR spectroscopy. The approximate combined concentration of IDI and NCI observed in the mixture was 30 mM.

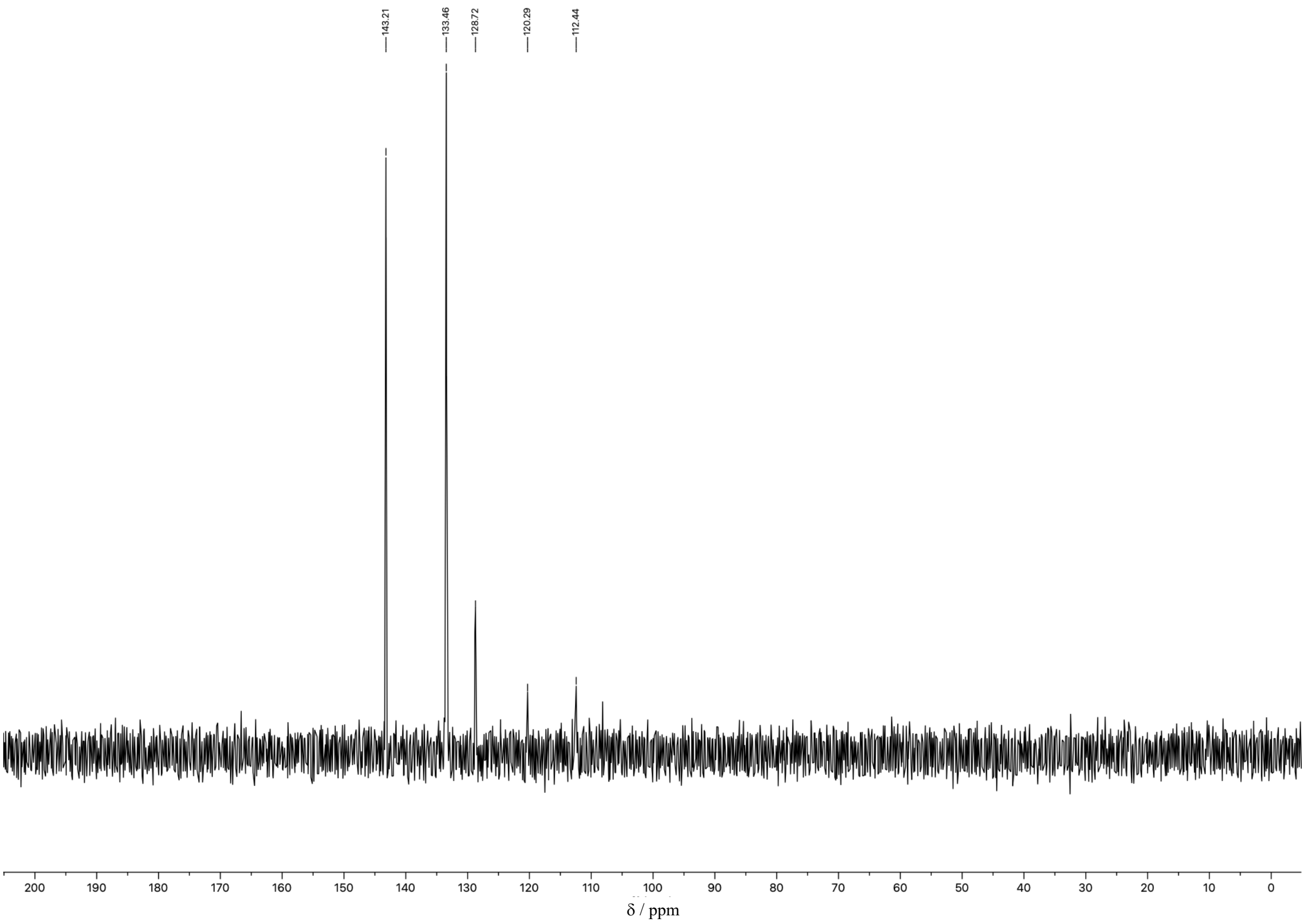

Fig. S18 ^{13}C NMR spectrum of the reaction of $(N^{13}CS)_2$ (50 mM) with Imidazole (100 mM) at pH 7. δ 112 ppm = HCN, δ 120 ppm = , δ 128 ppm = NCO^-, δ 133 ppm = NCS^-, and δ 143 ppm = IDI (imine C).

Attempting the reaction of 2-aminoimidazole with $(NCS)_2$.

KS^{13}CN (10 mg, 0.10 mmol) was dissolved in 10% D_2O in H_2O (1.0 mL) along with a trace amount of $FeSO_4 \cdot 7H_2O$ (1 mM). Upon addition of 30% H_2O_2 solution (42 µL, 0.20 mmol) the mixture turned a blood red colour indicating the formation of $[Fe(SCN)(H_2O)_5]^{2+}$. The reaction was examined by ^{13}C NMR spectroscopy and complete conversion to $(NCS)_2$ was observed. Then 2-aminoimidazole sulphate was added (13 mg, 0.1 mmol) and the pH of the reaction mixture was adjusted slowly raised and the reaction monitored by ^{13}C NMR spectroscopy. As the pH of the mixture rose, rapid decomposition of the $(NCS)_2$ was observed at pH 6.5, well below the pK_a of 2AI.

Phosphate activation reactions involving NCI or IDI

Purification procedure for commercial NCI

We have noticed that in our work with NCI, both in mononucleotide reaction and in the RNA ligation experiments, purification of commercial NCI was needed to observe good levels of reactivity.

Commercial NCI (100 mg) was weighted in a vial and chloroform was added (1-2 mL), the solution was sonicated for a few minutes. At this point most of the solid will be dissolved. The solution was then filtered over a sintered funnel with porosity grade of at least 3. The clear solution was then blown down under nitrogen and the white or pale-yellow solid was stored under nitrogen at -20 ºC.

If the solid after purification still has a strong yellow colour, the process is best repeated, until a pale yellow or ideally white solid is obtained. The purified solid is best used immediately but can be stored for prolonged periods of time if handled carefully.

Reaction of 3'-AMP with NCI derived from $(N^{13}CS)_2$

A sample of 0.5 mL 30 mM NCI/IDI mixture was prepared according to the procedure described above and 3′-AMP disodium salt (5.0 mg, 0.0125 mmol) was added, and the reaction was monitored by ^{31}P NMR spectroscopy. A maximum yield of 18% cyclic phosphate was observed after 32 h when all the NCI/IDI was consumed.

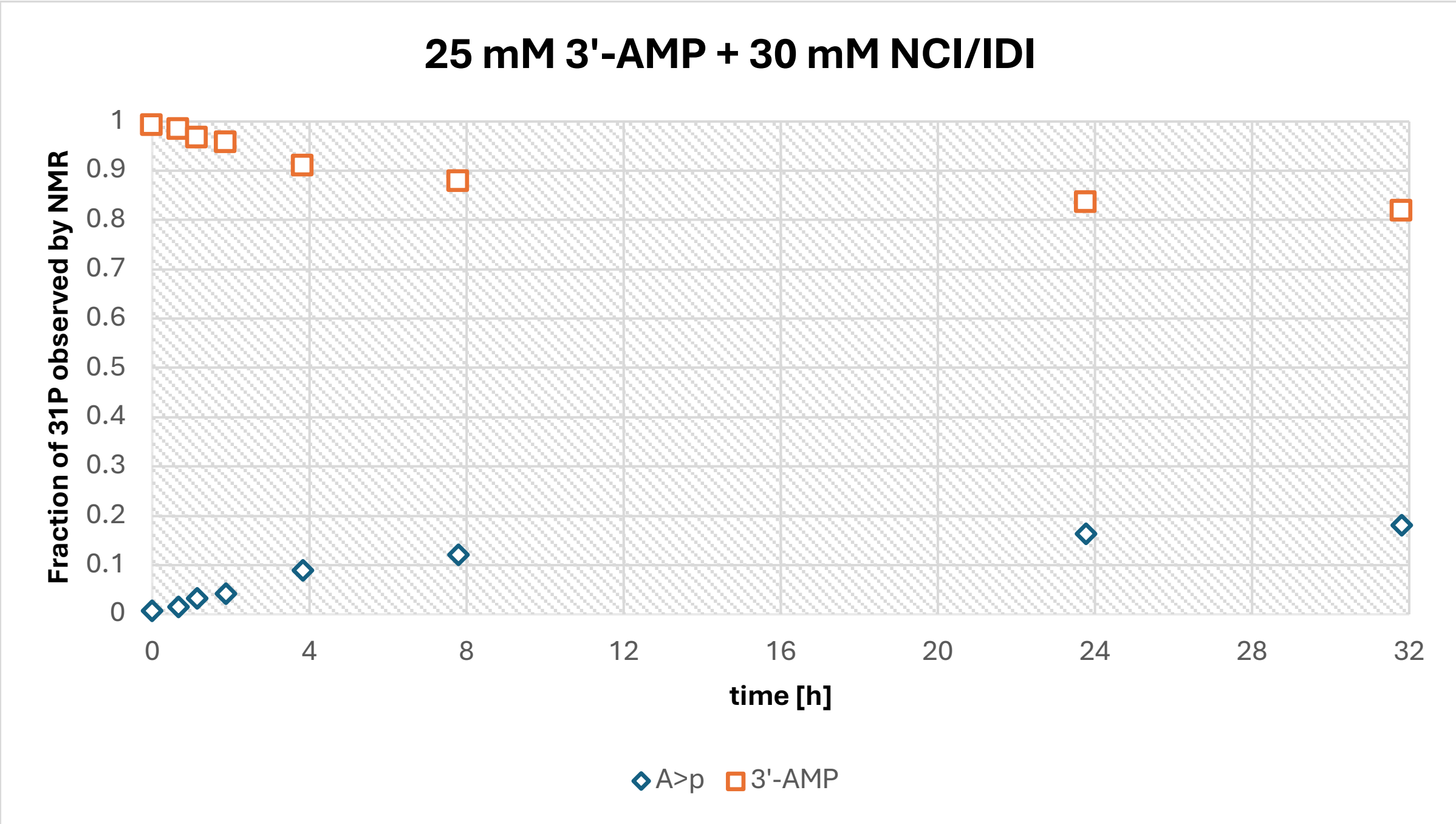

Fig. S19 Fraction of ^{31}P observed by ^{31}P NMR spectroscopy of the reaction mixture of NCI/IDI (30 mM) mixture derived from $(N^{13}CS)_2$ and 3′-AMP (25 mM) at various time points.

Reaction of 3′-AMP with NCI

3′-AMP disodium salt (5.0 mg, 0.0125 mmol) was dissolved in 10% D_2O in H_2O (0.5 mL) and the solution adjusted to pH 6. NCI (1 equiv., 2 equiv., or 4 equiv.) was added and the solution was monitored by ^{31}P NMR spectroscopy.

3′-AMP	NCI	Max yield of A>P	rxn time at max yield
25 mM	25 mM	58%	24 h
25 mM	50 mM	61%	4 h
25 mM	100 mM	77%	4 h

Table S2 Optimisation of phosphate activation with NCI.

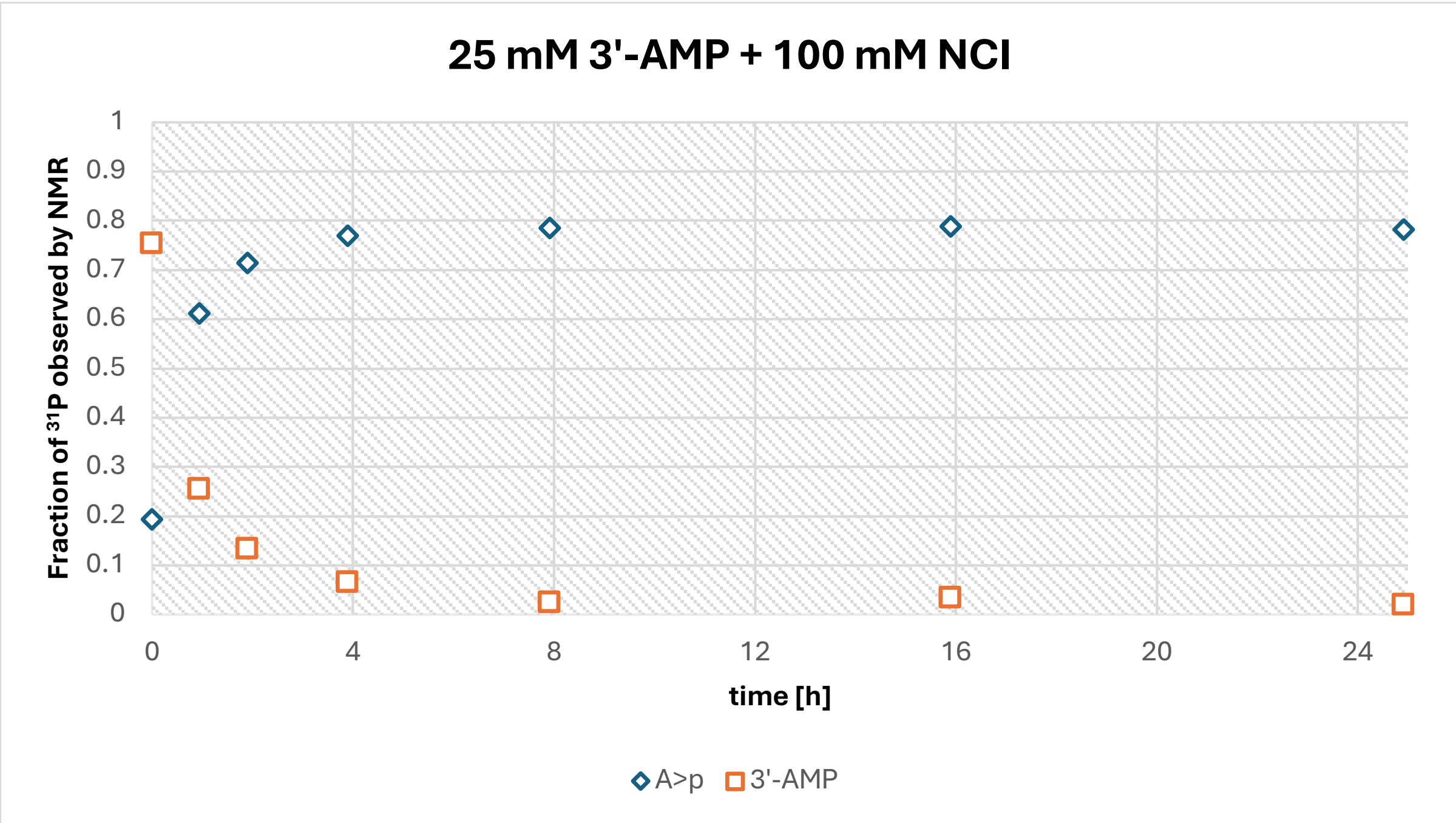

Fig. S20 Fraction of ^{31}P observed by ^{31}P NMR spectroscopy of the reaction mixture of NCI (100 mM) and 3′-AMP (25 mM) at various time points.

KS^{13}CN (20 mg, 0.20 mmol) was dissolved in 10% D_2O in H_2O (0.5 mL), 30% H_2O_2 solution (83 µL, 0.40 mmol) was added. After 3 h imidazole was added (14 mg, 0.2 mmol) and the reaction was examined by ^{1}H and ^{13}C NMR spectroscopy, to determine the approximate concentration of NCI/IDI as 70 mM. Then 3′-AMP disodium salt was added (5.0 mg, 0.025 mmol) and the reaction was monitored by ^{31}P NMR spectroscopy. After 48 h 43% of cAMP formed and all the NCI/IDI was consumed. A small amount (<10%) of 2'-hydorxy modified product was also observed.

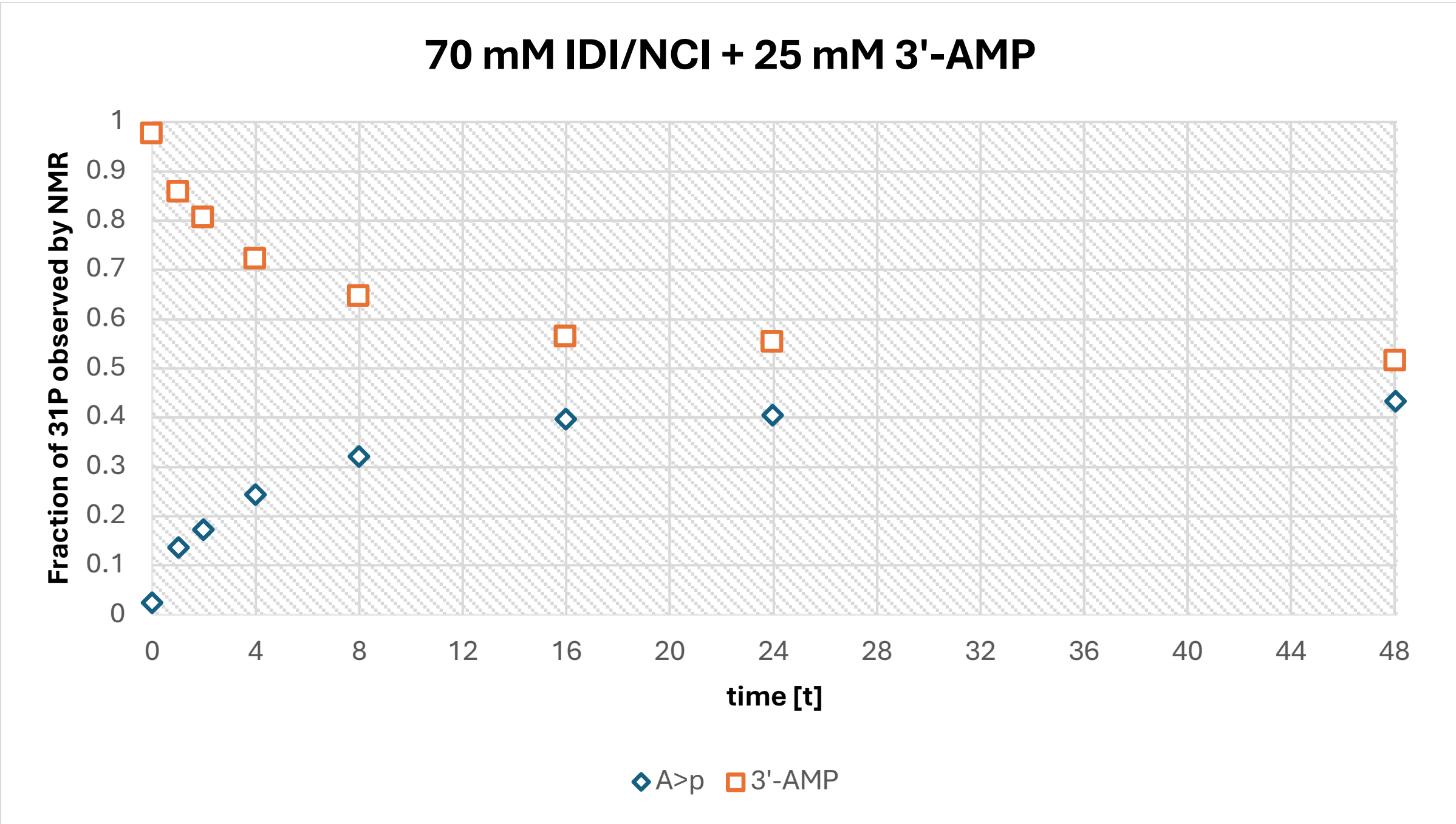

Fig. S21 Fraction of ^{31}P observed by ^{31}P NMR spectroscopy of the reaction mixture of NCI/IDI (70 mM) mixture derived from $(N^{13}CS)_2$ and 3′-AMP (25 mM) at various time points.

Reaction of 5′-AMP with NCI

5′-AMP disodium salt (5.0 mg, 0.0125 mmol) was dissolved in 10% D_2O in H_2O (0.5 mL) and the solution adjusted to pH 6. NCI (1 equiv., 2 equiv., or 4 equiv.) was added and the solution was monitored by ^{31}P NMR spectroscopy.

5′-AMP	NCI	Max yield of ImpA	rxn time at max yield
25 mM	25 mM	0%	/
25 mM	50 mM	5%	4 h
25 mM	100 mM	13%	4 h

Table S3 Optimisation of ImpA formation using NCI.

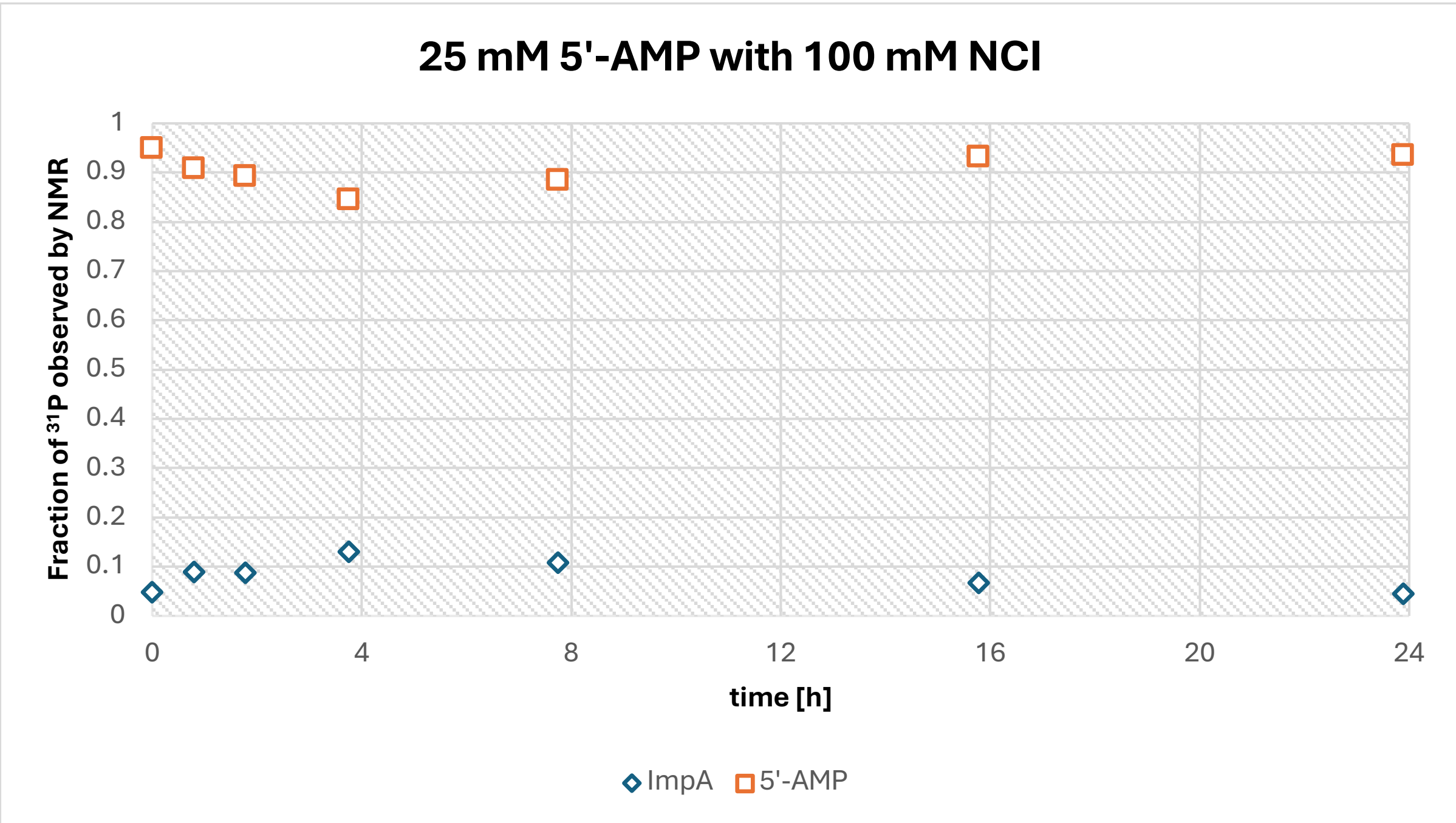

Fig. S22 Fraction of ^{31}P observed by ^{31}P NMR spectroscopy of the reaction mixture of NCI (100 mM) and 5′-AMP (25 mM) at various time points. Only the two major species are included for clarity.

Reaction of 5′-AMP with NCI/IDI derived from $(N^{13}CS)_2$

A sample of 0.5 mL 56 mM NCI/IDI mixture was prepared according to the procedure described above and 5′-AMP disodium salt (5.0 mg, 0.0125 mmol) was added, and the reaction was monitored by ^{31}P NMR spectroscopy. At the 8 h timepoint the maximum yield of 8% ImpA was observed.

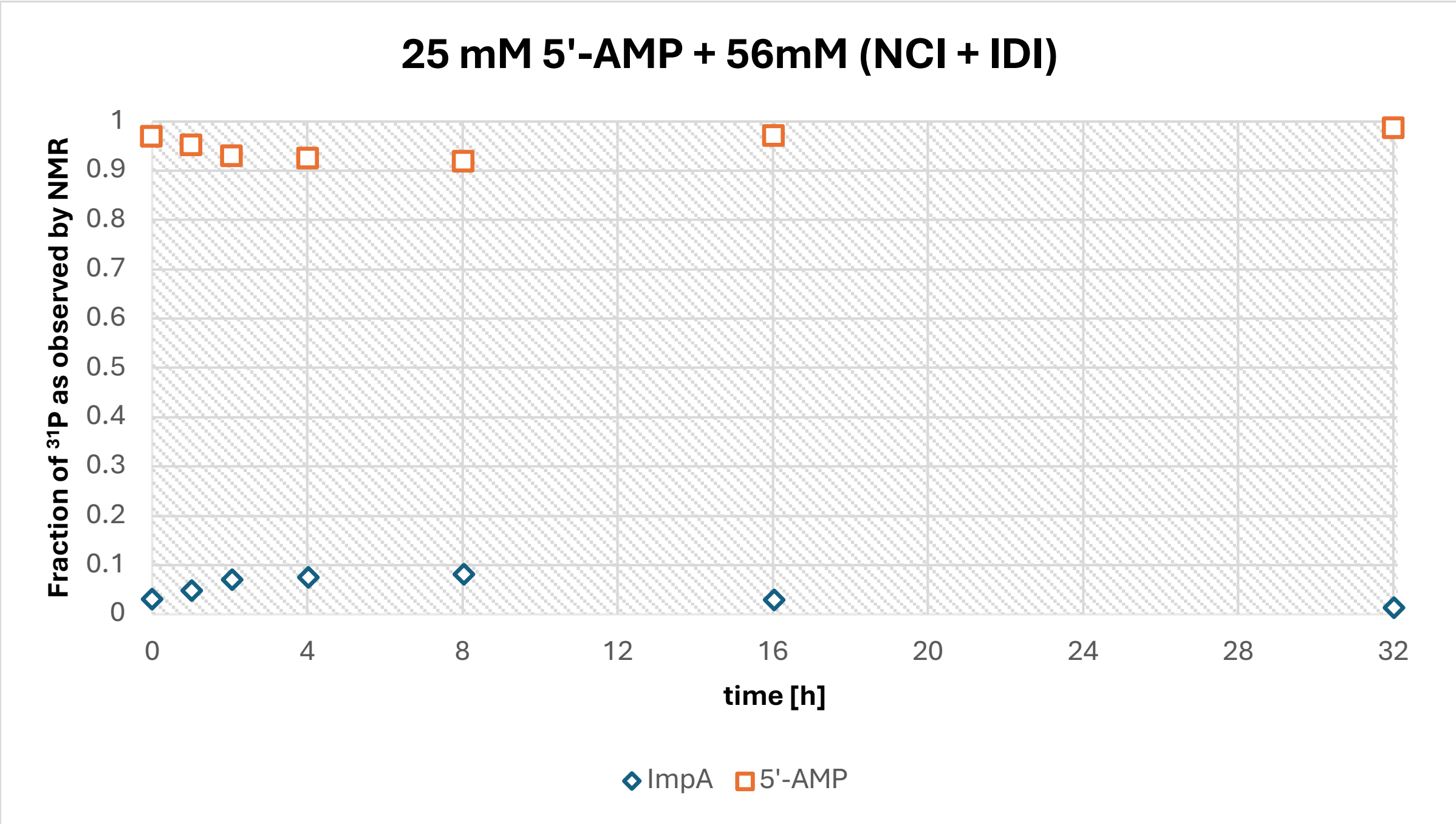

Fig. S23 Fraction of ^{31}P observed by ^{31}P NMR spectroscopy of the reaction mixture of NCI/IDI (56 mM) mixture derived from $(N^{13}CS)_2$ and 5′-AMP (25 mM) at various time points.

Reaction of 5′-AMP with NCI and imidazole

5′-AMP disodium salt (4.0 mg, 0.0125 mmol) and imidazole (3.4 mg, 0.050 mmol) was dissolved in 10% D_2O in H_2O (0.5 mL) and the solution adjusted to pH 7. NCI (5.0 mg, 0.050 mmol) was added and the solution was monitored by ^{31}P NMR spectroscopy. An increased maximum yield of 31% ImpA was observed after 4 h, however the amount and number of new side products increased under these conditions.

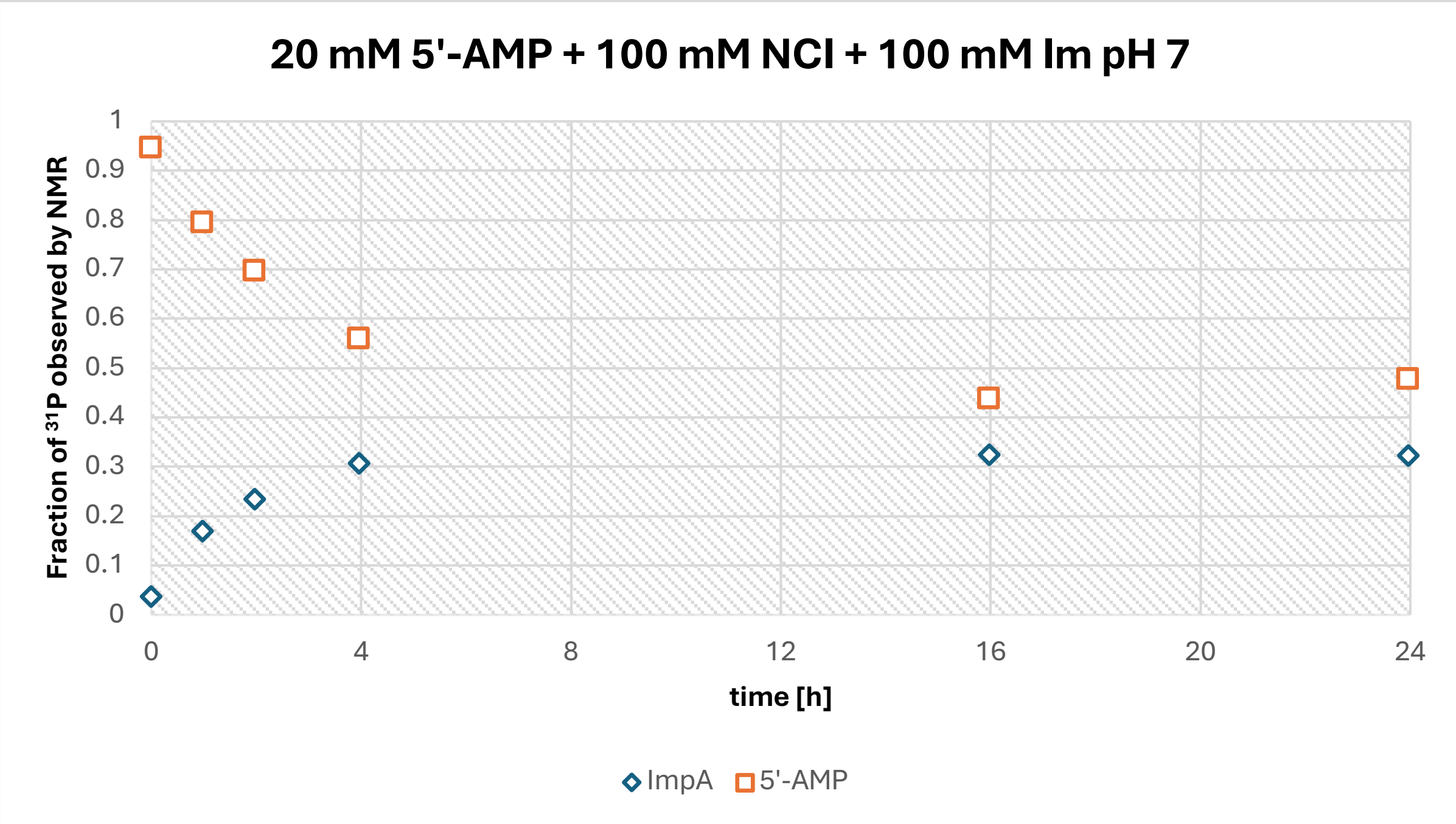

Fig. S24 Fraction of ^{31}P observed by ^{31}P NMR spectroscopy of the reaction mixture of NCI (100 mM) and 5′-AMP (25 mM) at various time points. Only the two major species are included for clarity.

Hydrolysis of NCI in imidazole buffer at pH 6.2 in the absence and presence of pACC

NCI (2 mg, 0.021mmol) and imidazole buffer at pH 6.2 (50 µL, 1 M) was dissolved in 10% D_2O in H_2O (final volume 0.5 mL) and the hydrolysis of NCI was monitored by ^{1}H NMR in the absence of or with pACC (2 mg, 2.089 µmol). No formation of the 5′-phosphorimidazolide was detected by ^{31}P NMR. We can clearly see that the rate of hydrolysis of NCI in the presence of the 5′-phosphorilated trinucleotide is almost twice as fast.

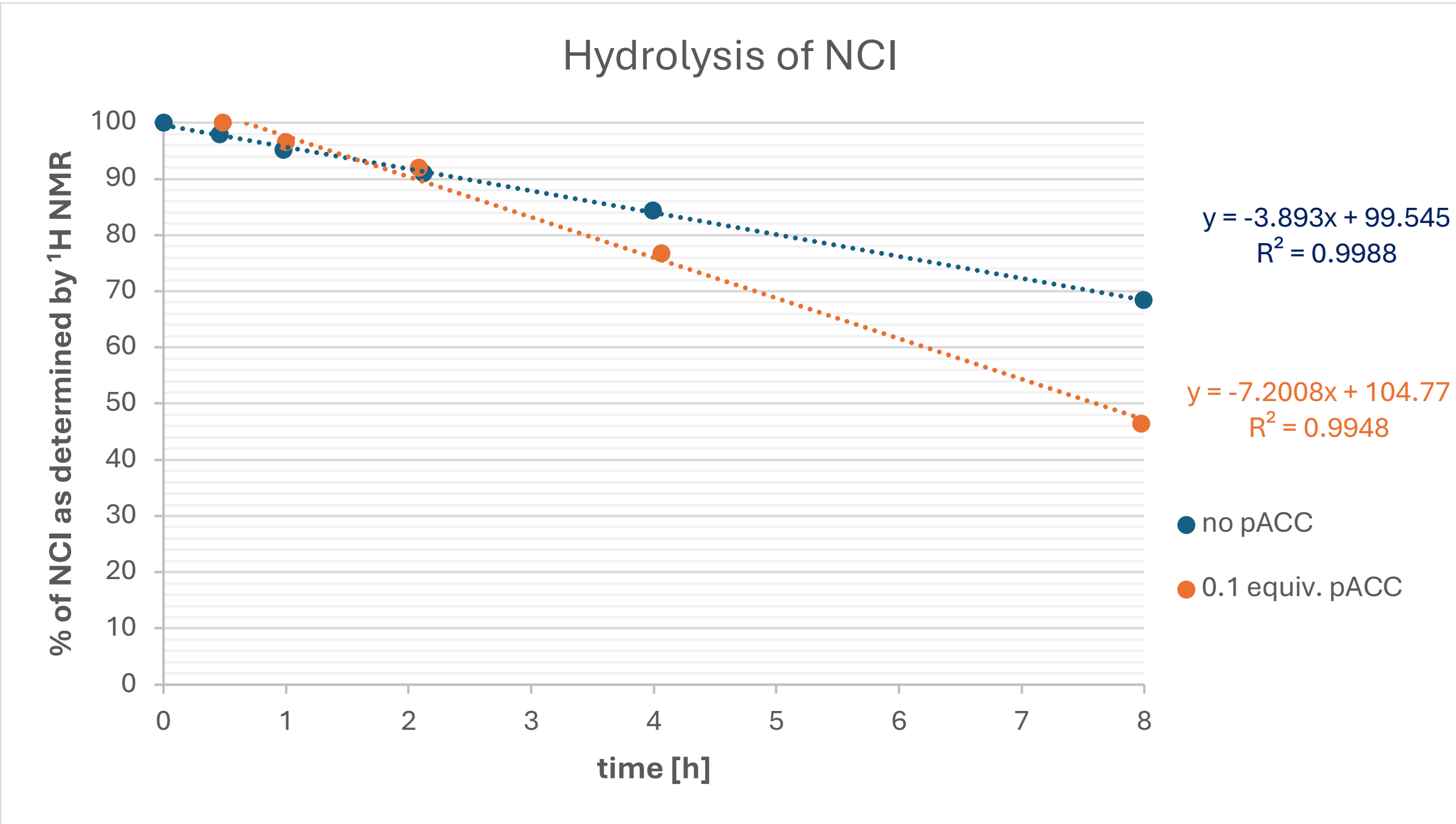

Fig. S25 Percentage of NCI as determined by 1H NMR plotted against time for a reaction monitoring the hydrolysis of NCI at pH 6.2 in the presence and absence of pACC.

Gel results

Detailed discussion of the NCI enabled RNA ligation:

Since the prebiotically plausible synthesis of NCI results in a complex mixture of products, we decided to start the optimisation of the nicked duplex ligation system with commercial NCI, before testing the optimized conditions on our real system (All ligation reactions were performed at room temperature unless otherwise stated). In the absence of divalent metal ions, no ligation was observed. We first tested the effect of Mg^{2+} concentration on the ligation system. Disappointingly, only minimal conversion (2% or lower) was achieved for a range of magnesium concentrations (10 mM – 500 mM) even after 48 h (Fig. S26). From previous work on this nicked RNA duplex we knew that 10 mM Mn^{2+} gave a good ligation yield (100 mM NCI, 24 h, 78%). We propose a favourable arrangement enabled by the coordination of NCI activated 5′-phosphate and the neighbouring nucleotide phosphodiester with Mn^{2+} to hold the nucleotide in a reactive conformation (Fig. 3) based on literature evidence that suggests similar type of metal chelates in the hydrolysis of RNA backbones[18]. In a further metal screen of Ni^{2+}, Cu^{2+}, and Zn^{2+} at 10 mM and 100 mM concentrations (Figs. S26 and S27) all performed worse, confirming that balancing the metals' oxo- and azaphilicity was key to obtain good ligation yields. Mn^{2+} was thus used for the rest of the optimization.

To lower the demands placed upon the prebiotic synthesis of NCI, we investigated using lower concentrations of NCI. Pleasingly, lowering the concentration of NCI from 20 mM to 10 mM still gave a similar ligation yield (85% vs 83%) after 48 h and substantial drops in yield (33% after 48 h) were only observed at 5 mM NCI (Fig. S29). Interestingly, adding 20 or 50 mM imidazole to the ligation reaction using 20 mM NCI led to an increase in the yield to 90%, whereas higher concentrations of imidazole decreased the yield of ligated product (Fig. S30). This is most likely due to imidazole coordinating to Mn^{2+} and preventing it from forming the chelate that enables efficient ligation. This is further supported by the observation that increasing Mn^{2+} concentration in the presence of high imidazole concentration (200 mM) resulted in a steady increase in the yield of ligation from 38% at 10 mM $MnCl_2$ after 19 h of incubation to 91% ligation at 200 mM $MnCl_2$, before a drop in yield was observed at 500 mM $MnCl_2$, presumably due to the increased rate of RNA degradation (Fig. S33). Similarly, a drop in yield could be observed when 100 mM NCS^-, another competing ligand for Mn^{2+}, was added to the ligation system (Fig. S34). Adding an appropriate amount of imidazole might be beneficial as it shifts the NCI/IDI equilibrium towards IDI which is less hydrolytically labile,

thus extending the lifetime of the activating species without sequestering all of the Mn^{2+}. Having observed that a stoichiometric ratio of NCI:imidazole of 1:1 and imidazole:Mn^{2+} of 1:1 gave the best results, we employed NCI:imidazole:Mn^{2+} in a 1:1:1 ratio with each at a concentration of 20 mM. Pleasingly, 42% ligation could be observed after 2 h going up to 89% after 8 h and all the way to 99% after 48 h (Fig. 4A and Fig. S35). Performing the same reaction using a FAM-labelled ligator oligo gave a comparable result, showing that the nature of the fluorescent label does not affect the outcome of this chemical ligation (Fig. S38) We then attempted a ligation reaction under these conditions, but using our prebiotic route to form NCI/IDI, and at a range of Mn^{2+} concentrations (as we assumed the excess imidazole and $(NCS)_2$ present in the NCI/IDI mixture would coordinate and sequester all of the Mn^{2+} at low concentrations). At 50 mM Mn^{2+} we observed 44% ligation product after 43 h and 50% after 65 h. Further increases in manganese concertation did not improve the yield or the rate of the ligation (Fig. S39-S40). Wanting to quantify any off-template reactivity, we performed the same reaction in the absence of RNA template. No products whatsoever were observed within 48 h. Given the dramatic difference that the inclusion of Mn^{2+} made, we went back to evaluate the potential of $(NC)_2$ to act as a phosphate activating agent in the NCI-optimised system, however no ligation product could be observed (Fig. S41). Reaction of adenosine with $(NC)_2$ at pH 6 resulted in rapid attack of $(NC)_2$ by the 2′,3′-diol, leading to the cyclic iminocarbonate, which is likely the reason that the ligation reaction failed with $(NC)_2$ (Fig. S42). Whilst cyanogen does not seem to be compatible with an RNA ligation system that mimics extant biology *i.e.* a 5′-phosphate and a 2′,3′-diol terminus, it does not rule out the possibility that it could potentially be used with an RNA junction comprised of a 5′-OH and a 3′-phosphate terminus. Other potential phosphate activating agents such as cyanamide, cyanate, and diiminosuccinonitrile similarly failed to enable any RNA ligation to take place.

Screening of Mg^{2+} concentrations for RNA ligation in a nicked duplex with NCI

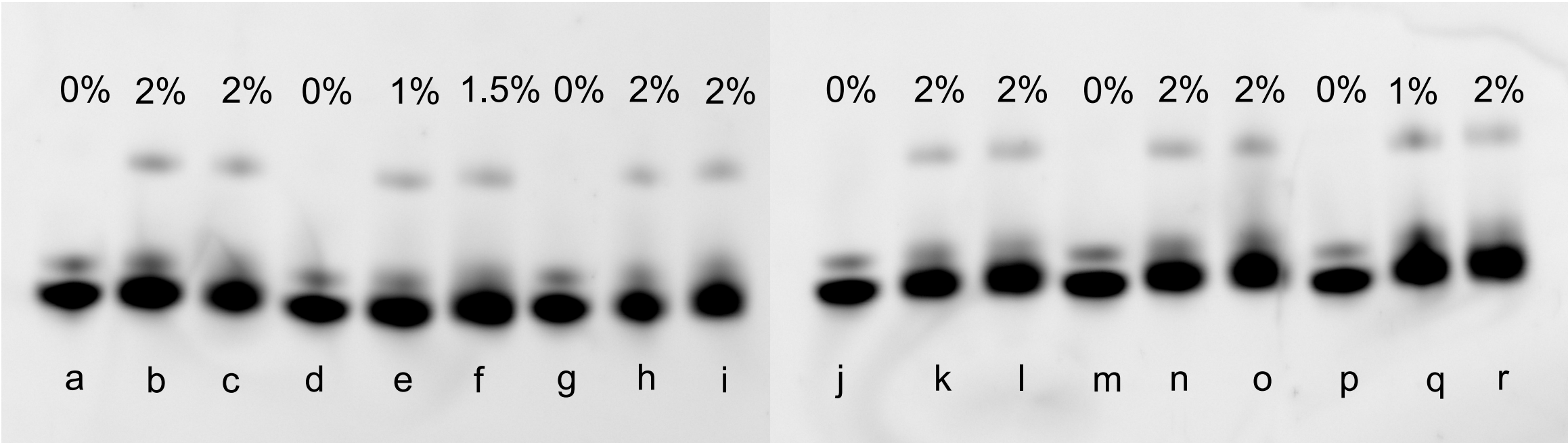

Fig. S26 PAGE analysis of in situ nicked-duplex RNA ligation using 5′-pUACUGGCA-Cy3 (10 µM), 5′-GAGAACC (20 µM), 5′-CCAGUAGGUUCUC (10 µM), NaCl (100 mM), $MgCl_2$ (XX mM), NCI (20 mM).

a) with $MgCl_2$ (10 mM), incubated for 0 h;

b) with $MgCl_2$ (10 mM), incubated for 24 h;

c) with $MgCl_2$ (10 mM), incubated for 48 h;

d) with $MgCl_2$ (20 mM), incubated for 0 h;

e) with $MgCl_2$ (20 mM), incubated for 24 h;

f) with $MgCl_2$ (20 mM), incubated for 48 h;

g) with $MgCl_2$ (50 mM), incubated for 0 h;

h) with $MgCl_2$ (50 mM), incubated for 24 h;

i) with $MgCl_2$ (50 mM), incubated for 48 h;

j) with $MgCl_2$ (100 mM), incubated for 0 h;

k) with $MgCl_2$ (100 mM), incubated for 24 h;

l) with $MgCl_2$ (100 mM), incubated for 48 h;

m) with $MgCl_2$ (200 mM), incubated for 0 h;

n) with $MgCl_2$ (200 mM), incubated for 24 h;

o) with $MgCl_2$ (200 mM), incubated for 48 h;

p) with $MgCl_2$ (500 mM), incubated for 0 h;

q) with $MgCl_2$ (500 mM), incubated for 24 h;

r) with $MgCl_2$ (500 mM), incubated for 48 h.

Metal screen (Ni^{2+}, Cu^{2+}, Zn^{2+}) for RNA ligation in a nicked duplex with NCI

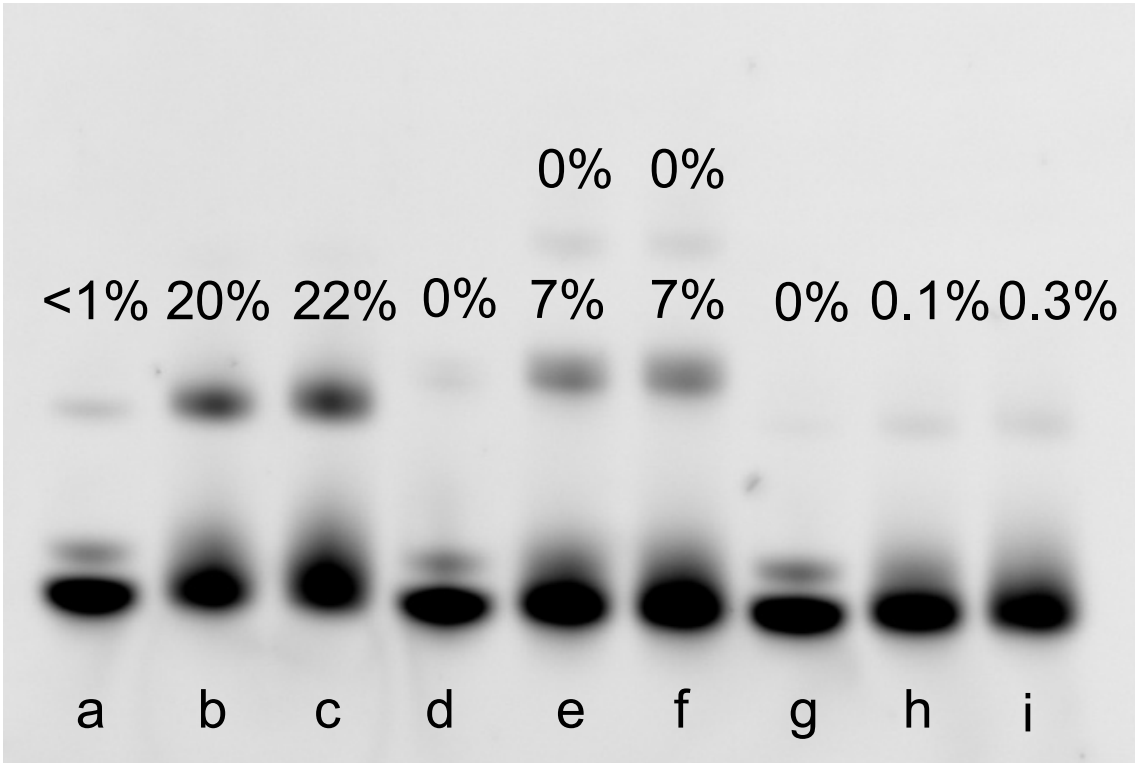

Fig. S27 PAGE analysis of in situ nicked-duplex RNA ligation using 5′-pUACUGGCA-Cy3 (10 μM), 5′-GAGAACC (20 μM), 5′-CCAGUAGGUUCUC (10 μM), NaCl (100 mM), MCl_2 (10 mM), NCI (20 mM).

a) with $NiCl_2$ (10 mM), incubated for 0 h;

b) with $NiCl_2$ (10 mM), incubated for 18 h;

c) with $NiCl_2$ (10 mM), incubated for 42 h;

d) with $CuCl_2$ (10 mM), incubated for 0 h;

e) with $CuCl_2$ (10 mM), incubated for 18 h;

f) with $CuCl_2$ (10 mM), incubated for 42 h;

g) with $ZnCl_2$ (10 mM), incubated for 0 h;

h) with $ZnCl_2$ (10 mM), incubated for 18 h;

i) with $ZnCl_2$ (10 mM), incubated for 42 h.

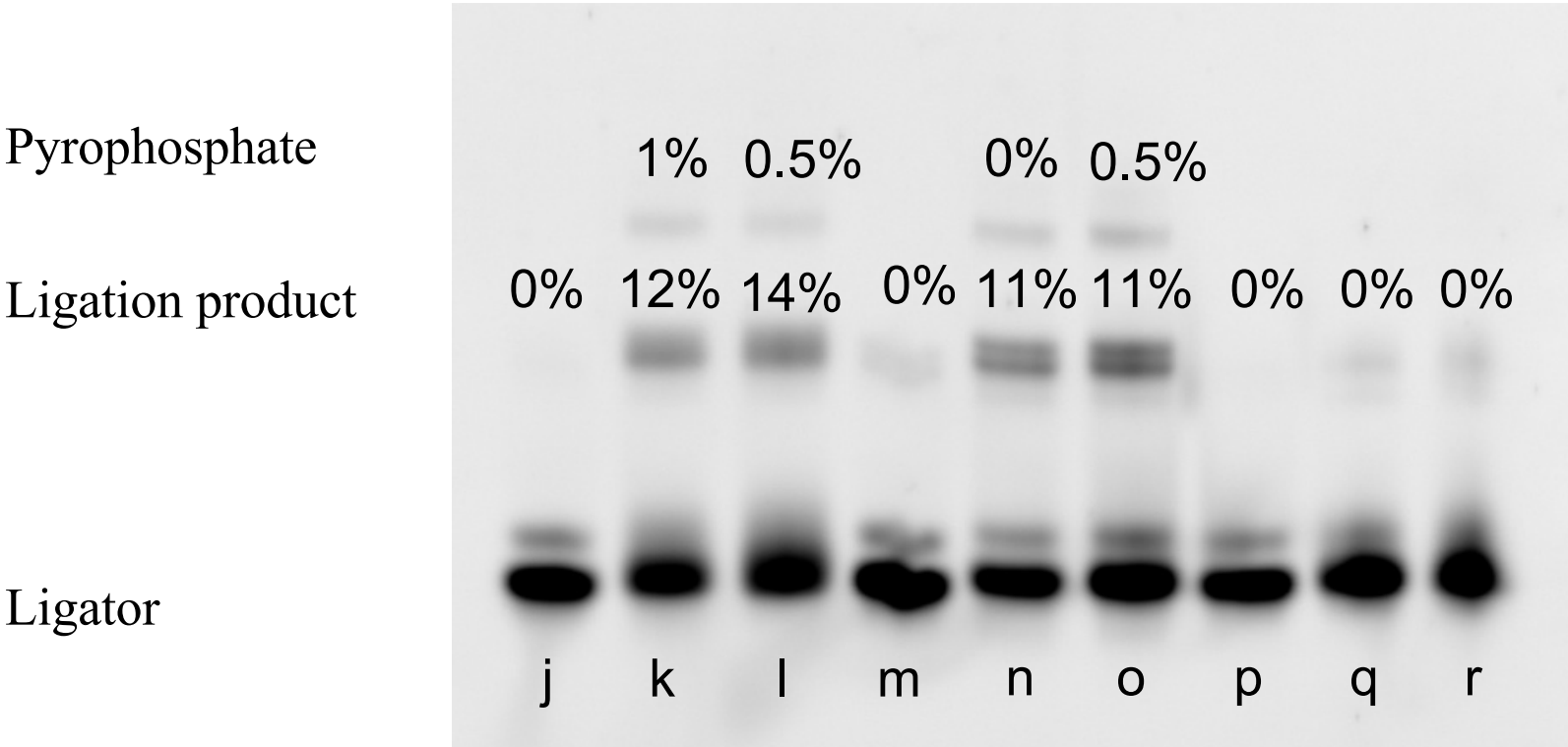

Fig. S28 PAGE analysis of in situ nicked-duplex RNA ligation using 5′-pUACUGGCA-Cy3 (10 µM), 5′-GAGAACC (20 µM), 5′-CCAGUAGGUUCUC (10 µM), NaCl (100 mM), MCl_2 (100 mM), NCI (20 mM).

j) with $NiCl_2$ (100 mM), incubated for 0 h;

k) with $NiCl_2$ (100 mM), incubated for 18 h;

l) with $NiCl_2$ (100 mM), incubated for 42 h;

m) with $CuCl_2$ (100 mM), incubated for 0 h;

n) with $CuCl_2$ (100 mM), incubated for 18 h;

o) with $CuCl_2$ (100 mM), incubated for 42 h;

p) with $ZnCl_2$ (100 mM), incubated for 0 h;

q) with $ZnCl_2$ (100 mM), incubated for 18 h;

r) with $ZnCl_2$ (100 mM), incubated for 42 h.

Screening NCI concentrations and their effect on the yield of RNA ligation in a nicked duplex

Ligation product

Ligator

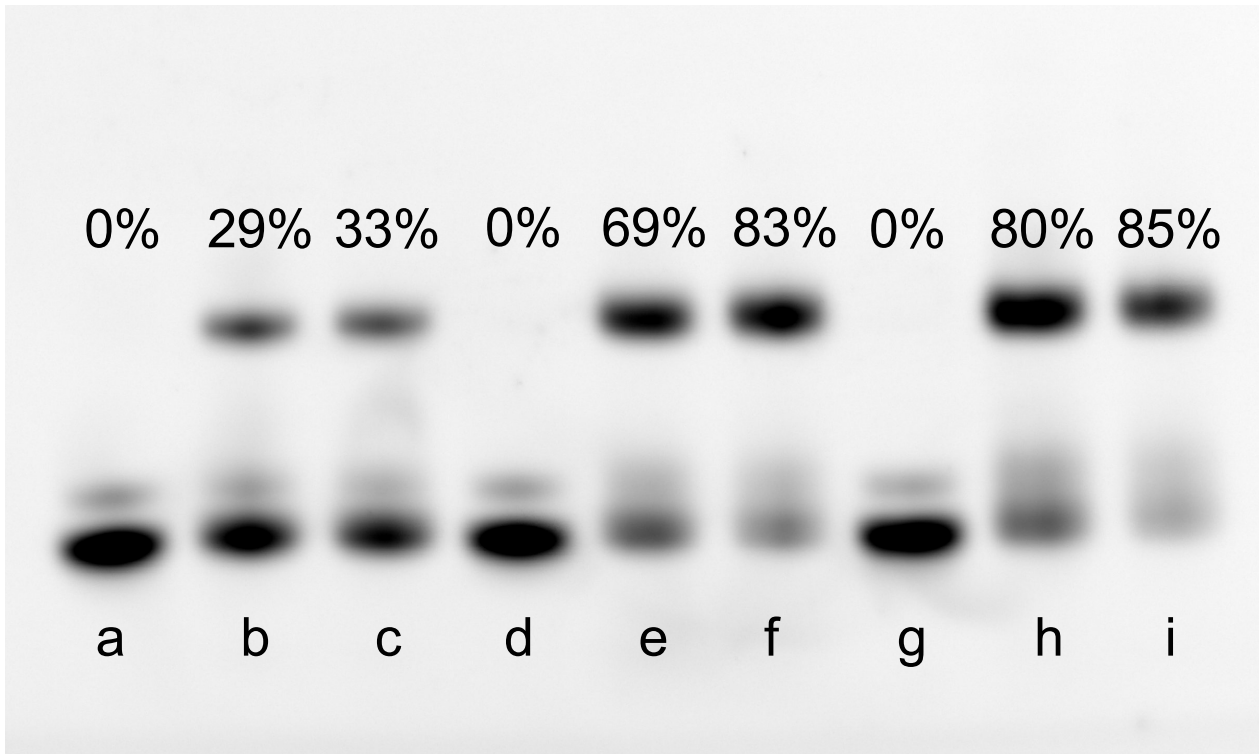

Fig. S29 PAGE analysis of in situ nicked-duplex RNA ligation using 5′-pUACUGGCA-Cy3 (10 µM), 5′-GAGAACC (20 µM), 5′-CCAGUAGGUUCUC (10 µM), NaCl (100 mM), $MnCl_2$ (10 mM), NCI (XX mM).

a) with NCI (5 mM), incubated for 0 h;

b) with NCI (5 mM), incubated for 24 h;

c) with NCI (5 mM), incubated for 48 h;

d) with NCI (10 mM), incubated for 0 h;

e) with NCI (10 mM), incubated for 24 h;

f) with NCI (10 mM), incubated for 48 h;

g) with NCI (20 mM), incubated for 0 h;

h) with NCI (20 mM), incubated for 24 h;

i) with NCI (20 mM), incubated for 48 h;

Optimisation of imidazole concentration for RNA ligation in a nicked duplex with NCI

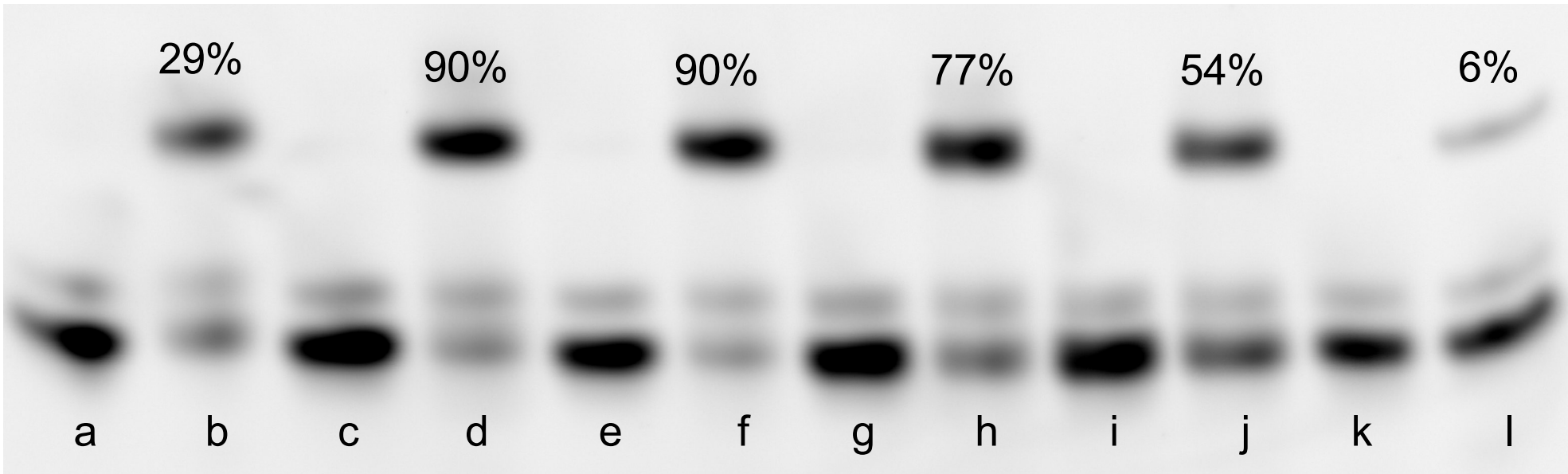

Fig. S30 PAGE analysis of in situ nicked-duplex RNA ligation using 5′-pUACUGGCA-Cy3 (10 µM), 5′-GAGAACC (20 µM), 5′-CCAGUAGGUUCUC (10 µM), NaCl (100 mM), $MnCl_2$ (10 mM), NCI (20 mM), Imidazole pH 6.2 (XX mM).

a) without imidazole, incubated for 0 h;

b) without imidazole, incubated for 18 h;

c) with imidazole (20 mM), incubated for 0 h;

d) with imidazole (20 mM), incubated for 18 h;

e) with imidazole (50 mM), incubated for 0 h;

f) with imidazole (50 mM), incubated for 18 h;

g) with imidazole (100 mM), incubated for 0 h;

h) with imidazole (100 mM), incubated for 18 h;

i) with imidazole (200 mM), incubated for 0 h;

j) with imidazole (200 mM), incubated for 18 h;

k) with imidazole (500 mM), incubated for 0 h;

l) with imidazole (500 mM), incubated for 18 h.

Effect of 0 mM vs 500 mM imidazole on the rate of ligation in a nicked RNA duplex with NCI

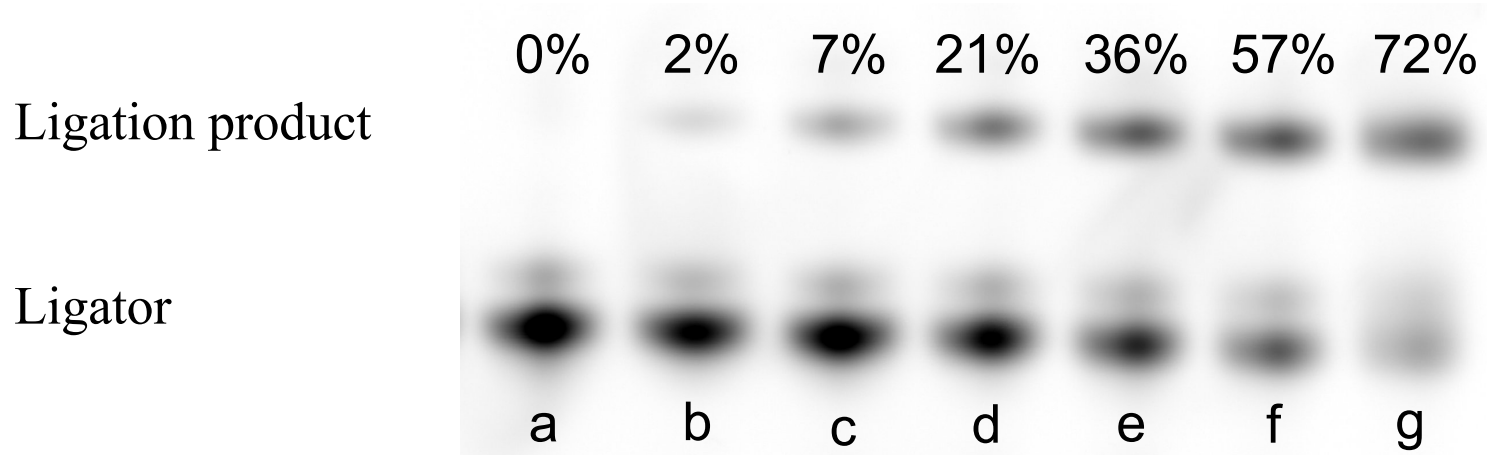

Fig. S31 PAGE analysis of in situ nicked-duplex RNA ligation using 5′-pUACUGGCA-Cy3 (10 µM), 5′-GAGAACC (20 µM), 5′-CCAGUAGGUUCUC (10 µM), NaCl (100 mM), $MnCl_2$ (10 mM), NCI (20 mM).

a) incubated for 0 h;

b) incubated for 0.5 h;

c) incubated for 1 h;

d) incubated for 2 h;

e) incubated for 4 h;

f) incubated for 6 h;

g) incubated for 16 h.

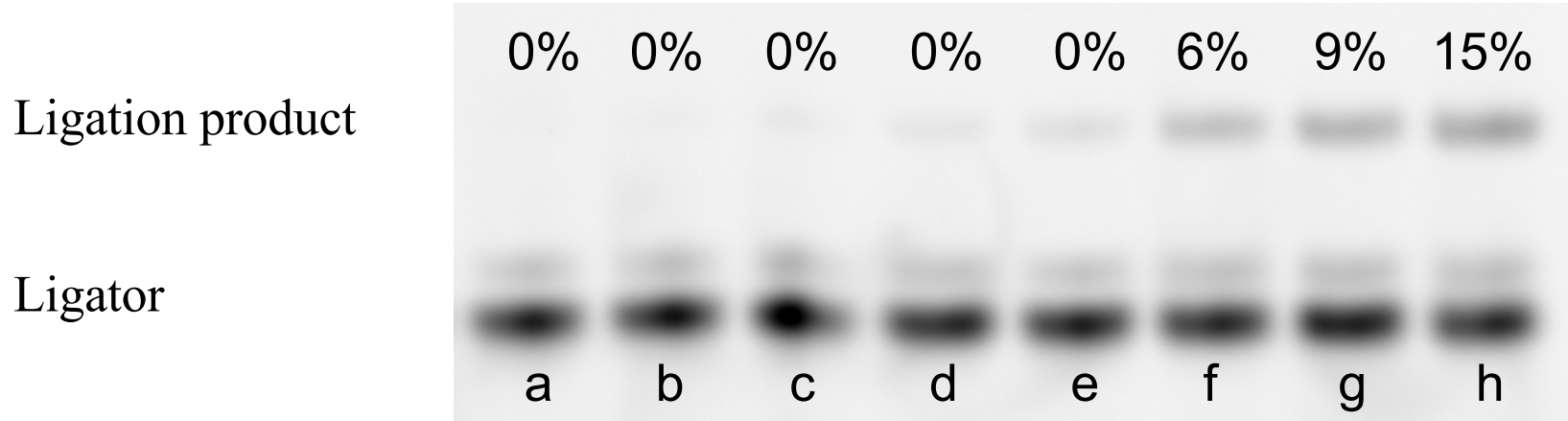

Fig. S32 PAGE analysis of in situ nicked-duplex RNA ligation using 5′-pUACUGGCA-Cy3 (10 µM), 5′-GAGAACC (20 µM), 5′-CCAGUAGGUUCUC (10 µM), NaCl (100 mM), $MnCl_2$ (10 mM), NCI (20 mM), imidazole pH 6.2 (500 mM).

a) incubated for 0 h;

b) incubated for 1 h;

c) incubated for 2 h;

d) incubated for 4 h;

e) incubated for 6 h;

f) incubated for 16 h;

g) incubated for 24 h;

h) incubated for 48 h.

Optimisation of Mn^{2+} concentration for RNA ligation in a nicked duplex with NCI

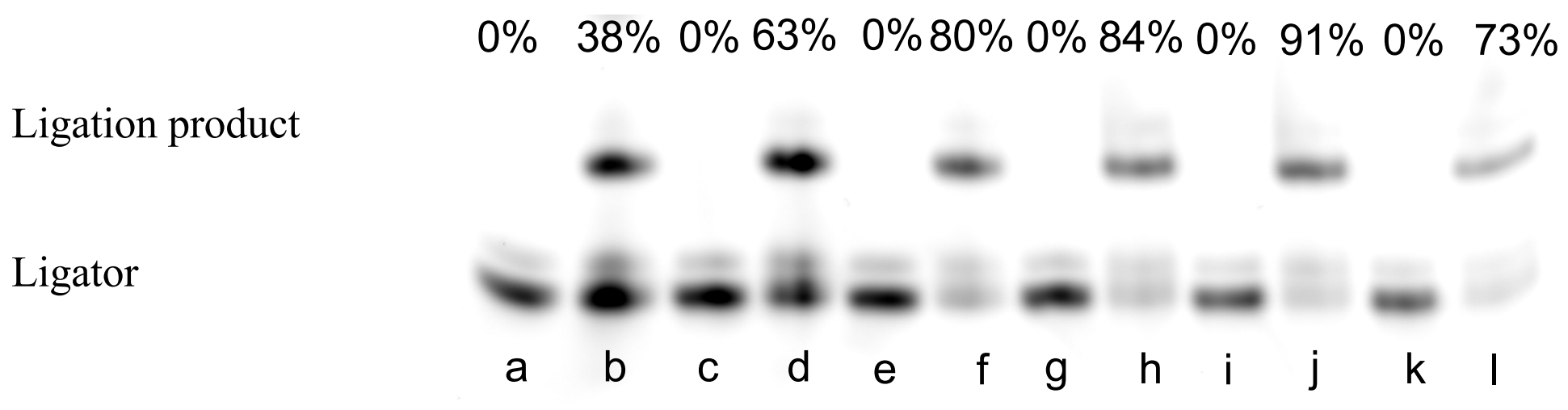

Fig. S33 PAGE analysis of in situ nicked-duplex RNA ligation using 5′-pUACUGGCA-Cy3 (10 µM), 5′-GAGAACC (20 µM), 5′-CCAGUAGGUUCUC (10 µM), NaCl (100 mM), $MnCl_2$ (XX mM), NCI (20 mM), and imidazole (200 mM).

a) with $MnCl_2$ (10 mM), incubated for 0 h;

b) with 10 mM $MnCl_2$ (10 mM), incubated for 19 h;

c) with $MnCl_2$ (20 mM), incubated for 0 h;

d) with $MnCl_2$ (20 mM), incubated for 19 h;

e) with $MnCl_2$ (50 mM), incubated for 0 h;

f) with $MnCl_2$ (50 mM), incubated for 19 h;

g) with $MnCl_2$ (100 mM), incubated for 0 h;

h) with $MnCl_2$ (100 mM), incubated for 19 h;

i) with $MnCl_2$ (200 mM), incubated for 0 h;

j) with $MnCl_2$ (200 mM), incubated for 19 h;

k) with $MnCl_2$ (500 mM), incubated for 0 h;

l) with $MnCl_2$ (500 mM), incubated for 19 h;

Effect of additional NCS⁻ on RNA ligation in a nicked duplex with NCI

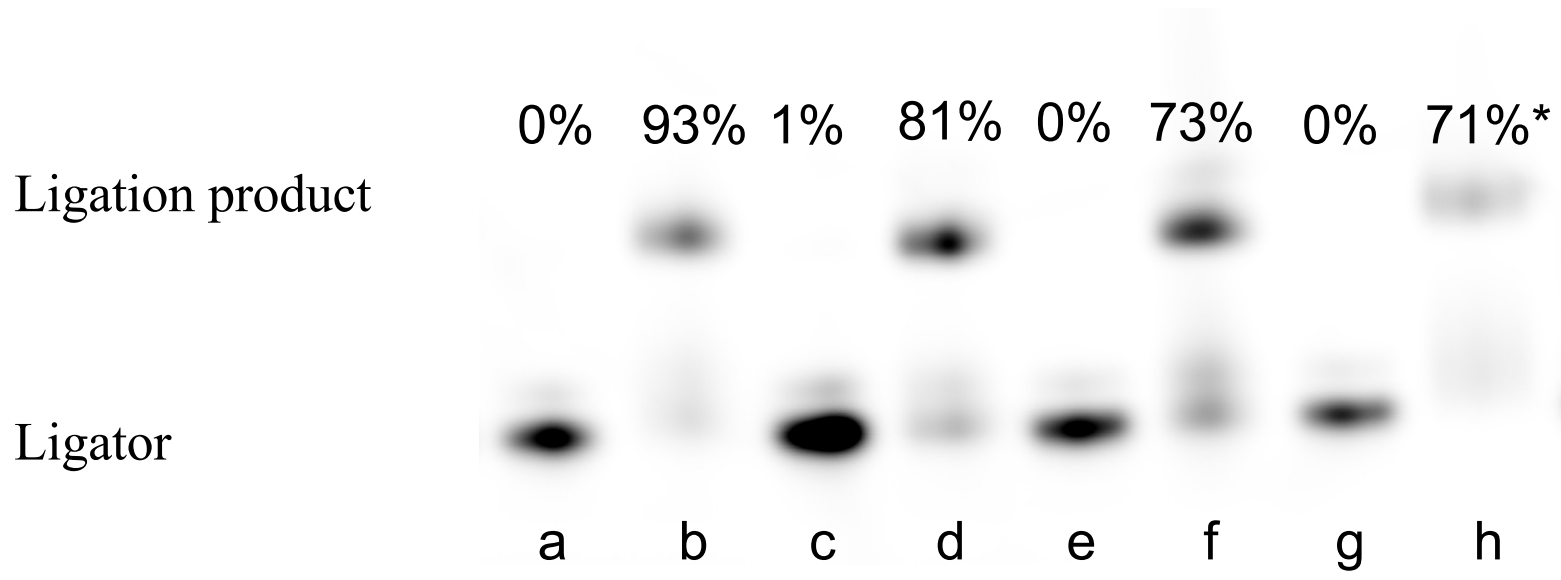

Fig. S34 PAGE analysis of in situ nicked-duplex RNA ligation using 5′-pUACUGGCA-Cy3 (10 µM), 5′-GAGAACC (20 µM), 5′-CCAGUAGGUUCUC (10 µM), NaCl (100 mM), $MnCl_2$ (10 mM), NCI (100 mM), imidazole pH 6.2 (XX mM), KSCN (XX mM).

a) without imidazole and without KSCN, incubated for 0 h;

b) without imidazole and without KSCN, incubated for 18 h;

c) with imidazole (100 mM) and without KSCN, incubated for 0 h;

d) with imidazole (100 mM) and without KSCN, incubated for 18 h;

e) with imidazole (100 mM) and KSCN (100 mM), incubated for 0 h;

f) with imidazole (100 mM) and KSCN (100 mM), incubated for 18 h;

g) without imidazole and with (100 mM), incubated for 0 h;

h) without imidazole and with (100 mM), incubated for 18 h.

* seems to have undergone substantial degradation, yield might be inaccurate.

Optimised RNA duplex ligation with commercial NCI

Ligation product

Ligator

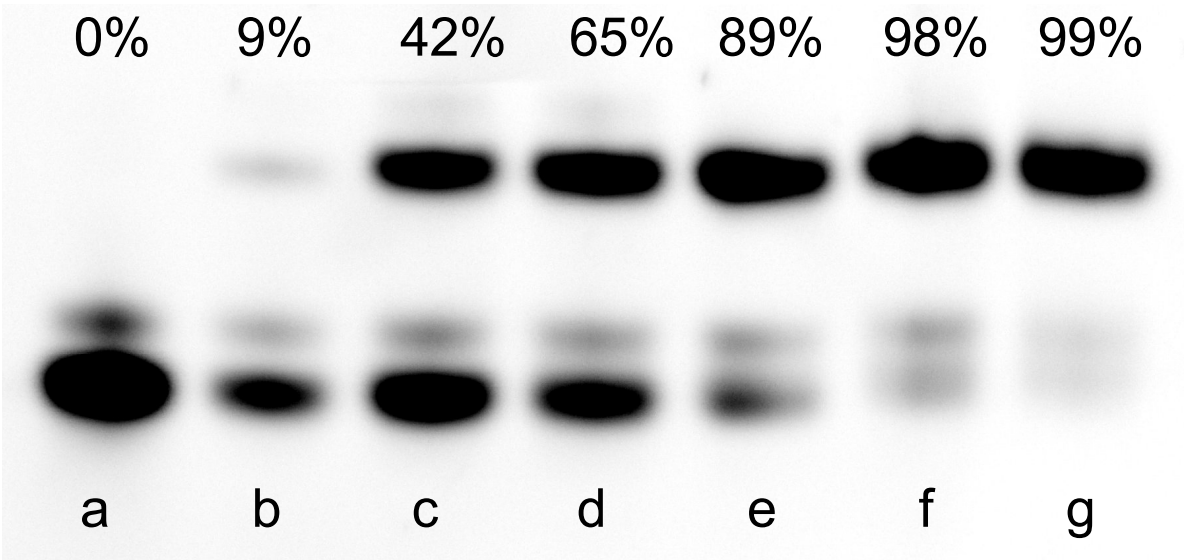

Fig. S35 PAGE analysis of in situ nicked-duplex RNA ligation using 5′-pUACUGGCA-Cy3 (10 µM), 5′-GAGAACC (20 µM), 5′-CCAGUAGGUUCUC (10 µM), NaCl (100 mM), $MnCl_2$ (20 mM), NCI (20 mM) and imidazole pH 6.2 (20 mM).

a) incubated for 0 h;

b) incubated for 1 h;

c) incubated for 2 h;

d) incubated for 4 h;

e) incubated for 8 h;

f) incubated for 24 h;

g) incubated for 48 h.

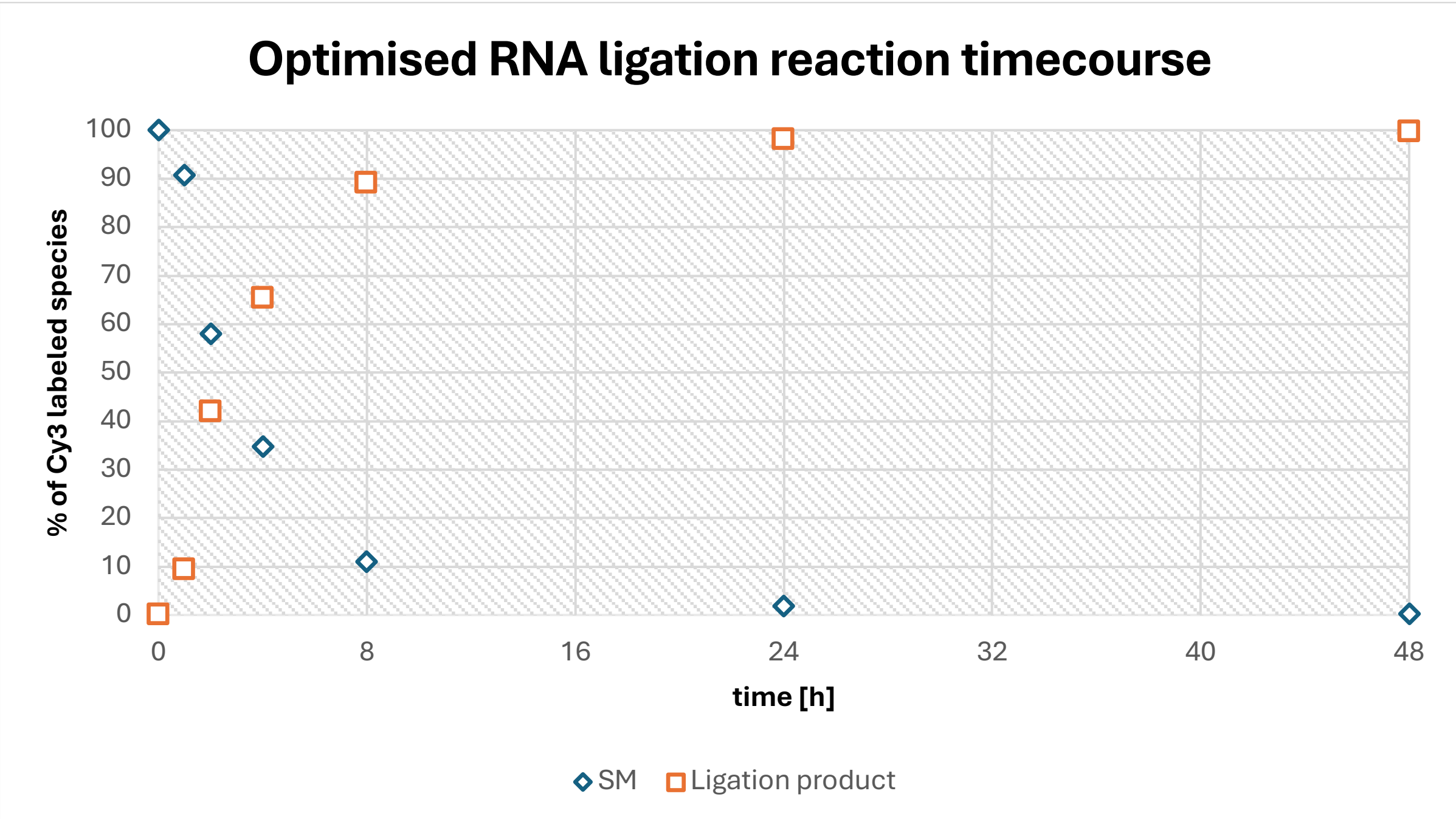

Fig. S36 Percentage of Cy 3 labeled species as determined by PAGE analysis of in situ nicked-duplex RNA ligation using 5′ - pUACUGGCA-Cy3 (10 µM), 5′ -GAGAACC (20 µM), 5′ -CCAGUAGGUUCUC (10 µM), NaCl (100 mM), $MnCl_2$ (20 mM), NCI (20 mM) and imidazole pH 6.2 (20 mM).

Plotting the natural logarithm of the starting material percentage against time for the first 8 hours of the reaction, during which 89% of the starting material is consumed, gives a straight line with an R^2 value of 0.9936, indicating a constant rate of reaction which matches first order kinetics. The half-life extracted from the data is 2.44 h.

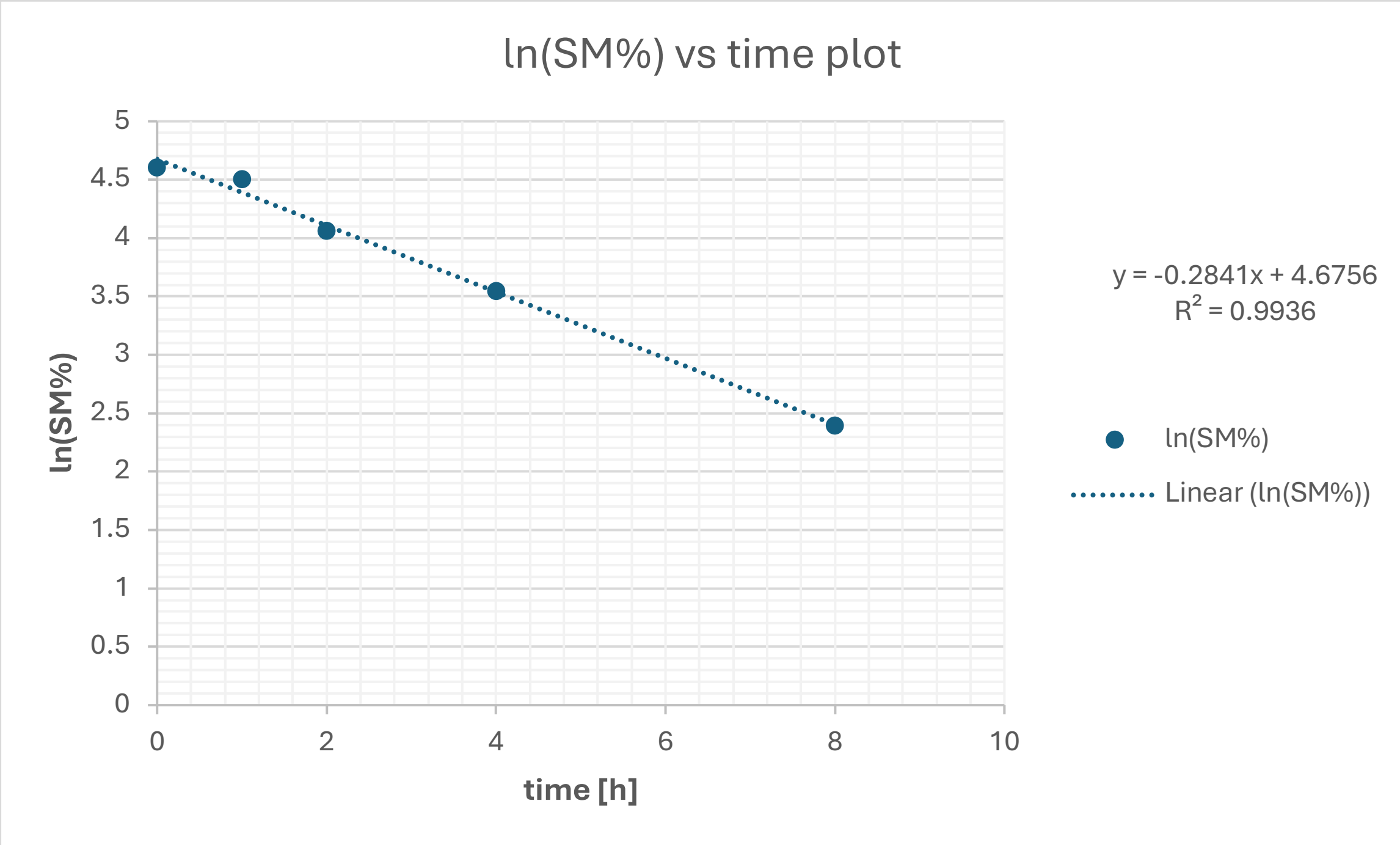

Fig. S37 Natural logarithm of starting material percentage plotted against time. Percentge of Cy 3 labeled starting material was determined by PAGE analysis of in situ nicked-duplex RNA ligation using 5′ - pUACUGGCA-Cy3 (10 µM), 5′ -GAGAACC (20 µM), 5′ -CCAGUAGGUUCUC (10 µM), NaCl (100 mM), $MnCl_2$ (20 mM), NCI (20 mM) and imidazole pH 6.2 (20 mM).

Optimised RNA duplex ligation with commercial NCI using a FAM-labelled ligator

Ligation product

Ligator

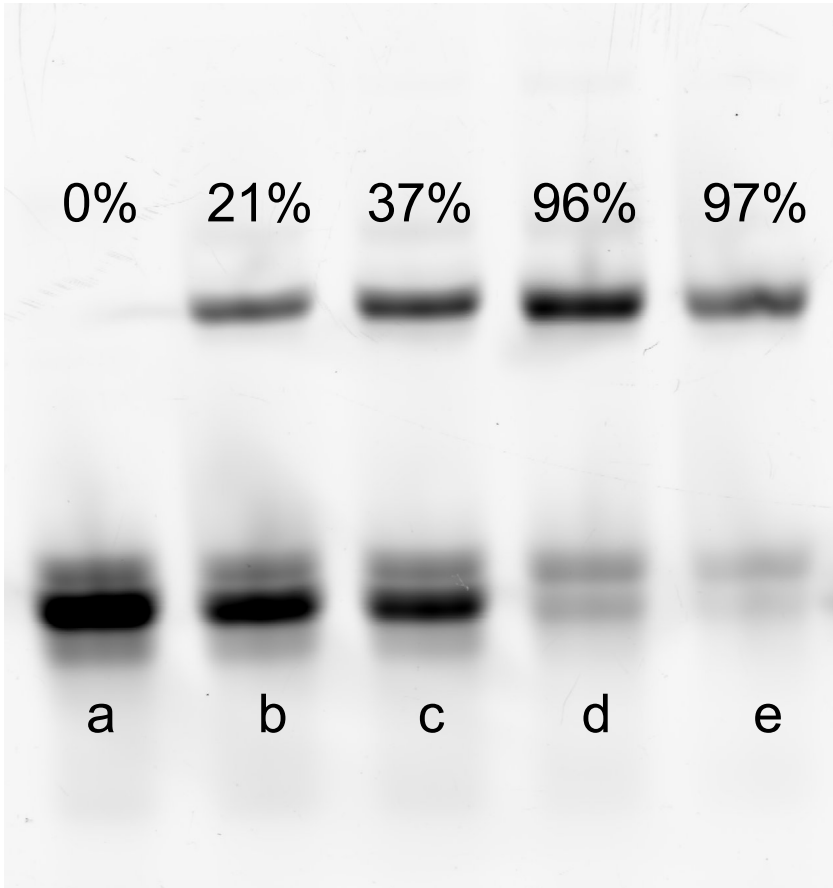

Fig. S38 PAGE analysis of in situ nicked-duplex RNA ligation using 5′-pUACUGGCA-FAM (10 µM), 5′-GAGAACC (20 µM), 5′-CCAGUAGGUUCUC (10 µM), NaCl (100 mM), $MnCl_2$ (20 mM), NCI (20 mM) and imidazole pH 6.2 (20 mM).

a) incubated for 0 h;

b) incubated for 1 h;

c) incubated for 2 h;

d) incubated for 19 h;

e) incubated for 24 h.

Optimised RNA duplex ligation with NCI prepared from $(NCS)_2$

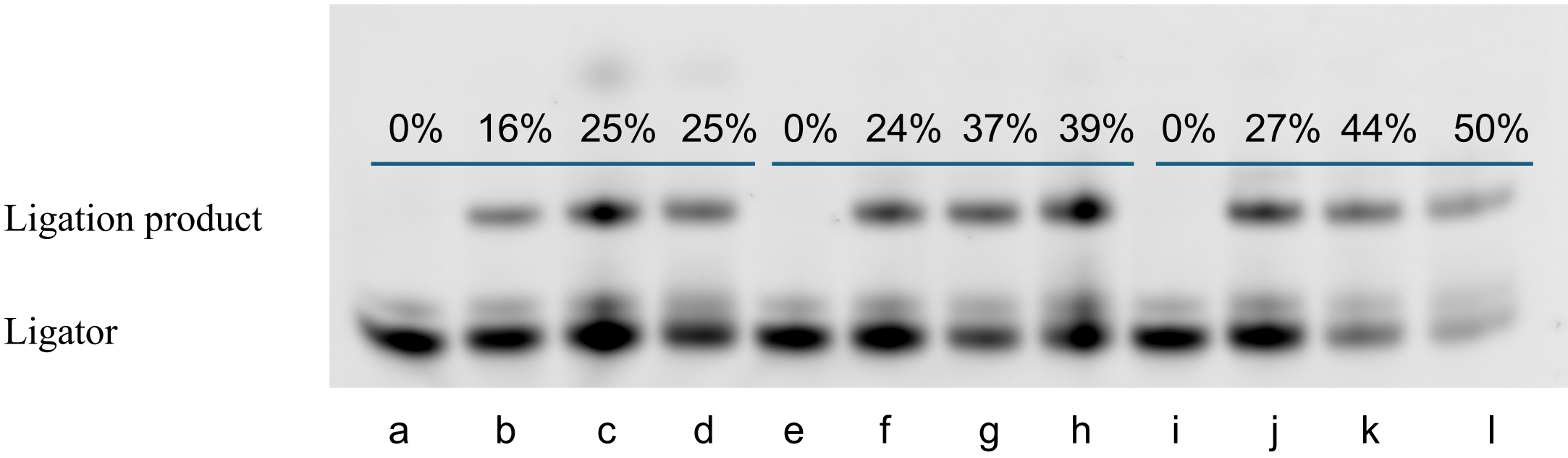

Fig. S39 PAGE analysis of in situ nicked-duplex RNA ligation using 5′-pUACUGGCA-Cy3 (10 µM), 5′-GAGAACC (20 µM), 5′-CCAGUAGGUUCUC (10 µM), NaCl (100 mM), $MnCl_2$ (X mM), NCI/IDI mixture prepared from $(NCS)_2$ (20 mM).

a) with $MnCl_2$ (10 mM), incubated for 0 h;

b) with $MnCl_2$ (10 mM), incubated for 17 h;

c) with $MnCl_2$ (10 mM), incubated for 43 h;

d) with $MnCl_2$ (10 mM), incubated for 65 h;

e) with $MnCl_2$ (20 mM), incubated for 0 h;

f) with $MnCl_2$ (20 mM), incubated for 17 h;

g) with $MnCl_2$ (20 mM), incubated for 43 h;

h) with $MnCl_2$ (20 mM), incubated for 65 h;

i) with $MnCl_2$ (50 mM), incubated for 0 h;

j) with $MnCl_2$ (50 mM), incubated for 17 h;

k) with $MnCl_2$ (50 mM), incubated for 43 h;

l) with $MnCl_2$ (50 mM), incubated for 65 h.

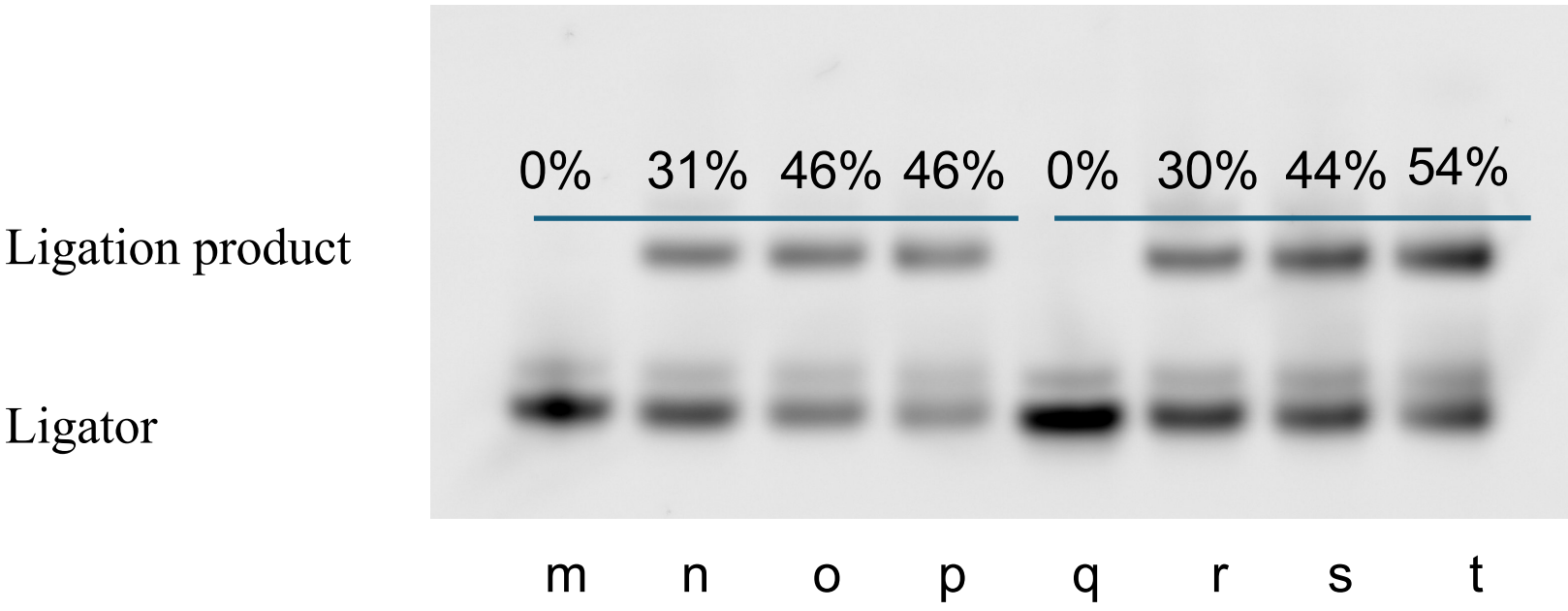

Fig. S40 PAGE analysis of in situ nicked-duplex RNA ligation using 5′-pUACUGGCA-Cy3 (10 µM), 5′-GAGAACC (20 µM), 5′-CCAGUAGGUUCUC (10 µM), NaCl (100 mM), $MnCl_2$ (X mM), NCI/IDI mixture prepared from $(NCS)_2$ (36 mM).

m) with $MnCl_2$ (100 mM), incubated for 0 h;

n) with $MnCl_2$ (100 mM), incubated for 17 h;

o) with $MnCl_2$ (100 mM), incubated for 43 h;

p) with $MnCl_2$ (100 mM), incubated for 65 h;

q) with $MnCl_2$ (200 mM), incubated for 0 h;

r) with $MnCl_2$ (200 mM), incubated for 17 h;

s) with $MnCl_2$ (200 mM), incubated for 43 h;

t) with $MnCl_2$, (200 mM) incubated for 65 h.

Attempt at using $(NC)_2$ as the phosphate activating agent in the RNA duplex ligation

Ligator

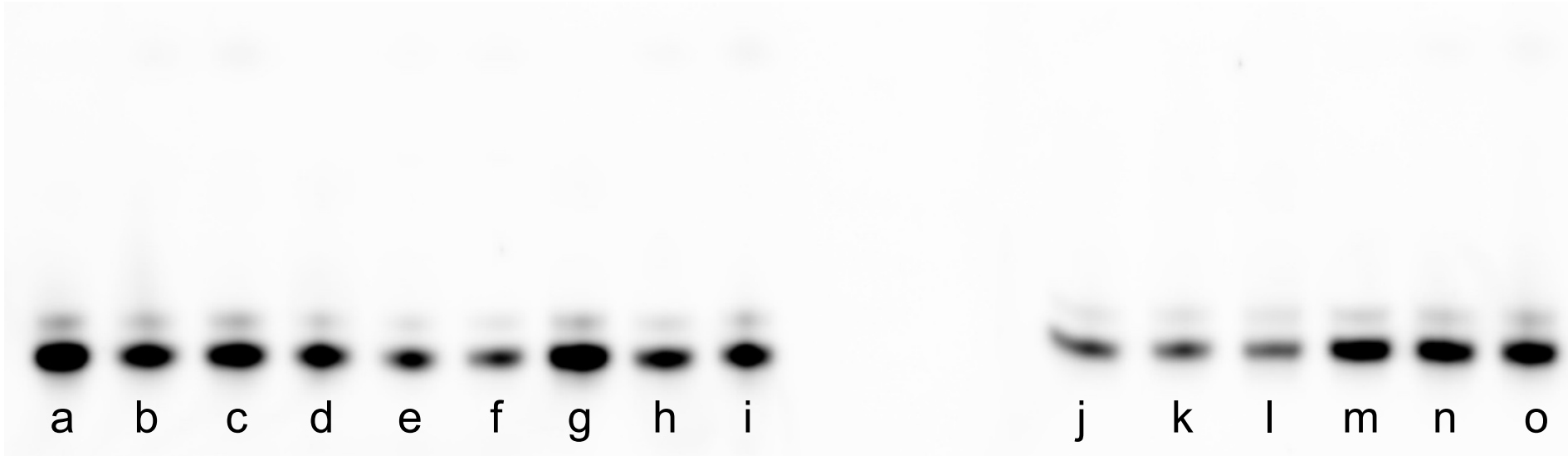

Fig. S41 PAGE analysis of in situ nicked-duplex RNA ligation using 5′-pUACUGGCA-Cy3 (10 µM), 5′-GAGAACC (20 µM), 5′-CCAGUAGGUUCUC (10 µM), NaCl (100 mM), and various combinations of $MnCl_2$ (XX mM), $(NC)_2$ (XX mM), imidazole pH 6.2 (XX mM) and 2AI (XX mM).

a) with $(NC)_2$ (100 mM), incubated for 0 h;

b) with $(NC)_2$ (100 mM), incubated for 17 h;

c) with $(NC)_2$ (100 mM), incubated for 24 h;

d) with $(NC)_2$ (100 mM) and $MnCl_2$ (100 mM), incubated for 0 h;

e) with $(NC)_2$ (100 mM) and $MnCl_2$ (100 mM), incubated for 17 h;

f) with $(NC)_2$ (100 mM) and $MnCl_2$ (100 mM), incubated for 24 h;

g) with $(NC)_2$ (100 mM), $MnCl_2$ (100 mM), and imidazole pH 6.2 (100 mM), incubated for 0 h;

h) with $(NC)_2$ (100 mM), $MnCl_2$ (100 mM), and imidazole pH 6.2 (100 mM), incubated for 17 h;

i) with $(NC)_2$ (100 mM), $MnCl_2$ (100 mM), and imidazole pH 6.2 (100 mM), incubated for 24 h;

j) with $(NC)_2$ (100 mM), $MnCl_2$ (100 mM), and 2AI (100 mM), incubated for 0 h;

k) with $(NC)_2$ (100 mM), $MnCl_2$ (100 mM), and 2AI (100 mM), incubated for 17 h;

l) with $(NC)_2$ (100 mM), $MnCl_2$ (100 mM), and 2AI (100 mM), incubated for 24 h;

m) with $(NC)_2$ (100 mM) and imidazole pH 6.2 (100 mM), incubated for 0 h;

n) with $(NC)_2$ (100 mM) and imidazole pH 6.2 (100 mM), incubated for 17 h;

o) with $(NC)_2$ (100 mM) and imidazole pH 6.2 (100 mM), incubated for 24 h.

Reaction of $(NC)_2$ with Adenosine

Adenosine (3 mg, 0.125 mmol) was dissolved in D_2O/H_2O (50 μL/425 μL) and the pH of the solution was adjusted to pH 6 with NaOH. A solution of (NC)2 (1M, 25 μL, 0.250 mmol) was then added to the reaction mixture and monitored by ^1H NMR spectroscopy.

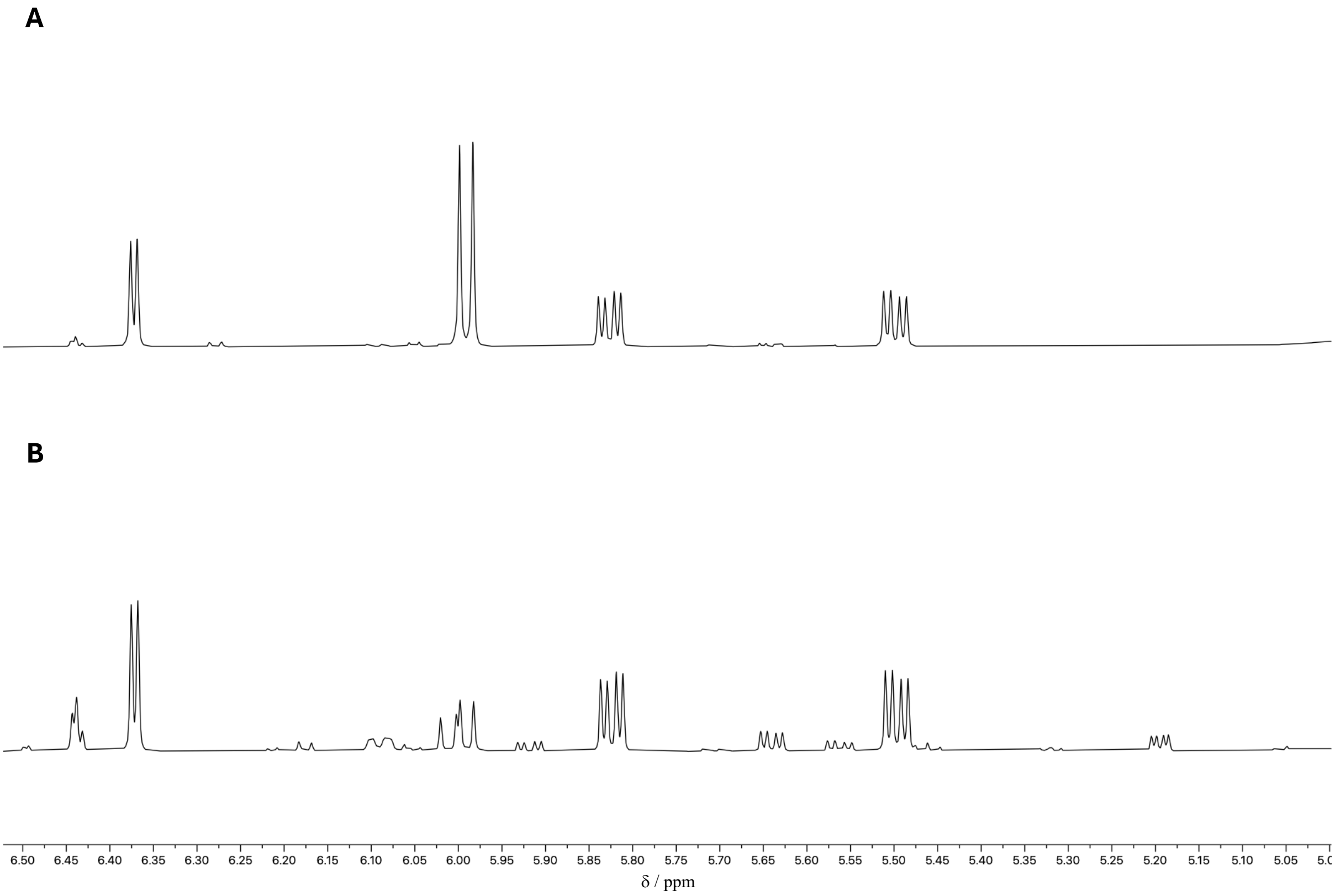

Fig. S42 Spectrum A – ^1H NMR spectrum of the reaction between Adenosine (25 mM) and $(NC)_2$ (50 mM) in 10% D_2O in H_2O recorded initially. Spectrum B – ^1H NMR spectrum of the reaction between Adenosine (25 mM) and $(NC)_2$ (50 mM) in 10% D_2O in H_2O recorded after 1 h.

Determining the regioselectivity of the ligation reaction.

A 1 mL RNA ligation using 5′-pUACUGGCA (10 µM), 5′-GAGAACC (20 µM), 5′-CCAGUAGGUUCUC (10 µM), NaCl (100 mM), $MnCl_2$ (20 mM), NCI (20 mM) and imidazole pH 6.3 (20 mM) was left for 48 h. The ligation product was extracted from an excised gel band as follows: The gel band was finely crushed and stirred with NaOAc (500 mM) overnight before the gel was removed by filtration. The filtrate was desalted using a Waters Sep-Pak® C18 Plus Short Cartridge (cartridge pre-washed with MeCN (10 mL) then water (10 mL) before sample loading, then the sample was eluted with 10 % aqueous MeCN. The eluent was then lyophilised and redissolved in water and further purified by HPLC.

To determine the ratio of 2′-5′ to 3′-5′ links in the phosphodiester bond formed in our ligation we adopted the method reported by Ferris *et al*.[19] To a solution of the ligation product (50 µM) in water was added 1.5 U of RNAse T2 (6 U/µL solution in 9 mM NH_4OAC buffer pH 4.5) and incubated at 35 °C for 16 h. The pH of the solution was then adjusted to pH 8 using dephosphorylation buffer and 4 U alkaline phosphatase (20 U/µL solution) and incubated for 1 h at 35 °C, at which point the temperature was raised to 65 °C and heated for 15 min. The sample was then analysed by HPLC chromatography, which indicated complete regioselectivity of the ligation reaction to produce exclusively 3′-5′ linked material.

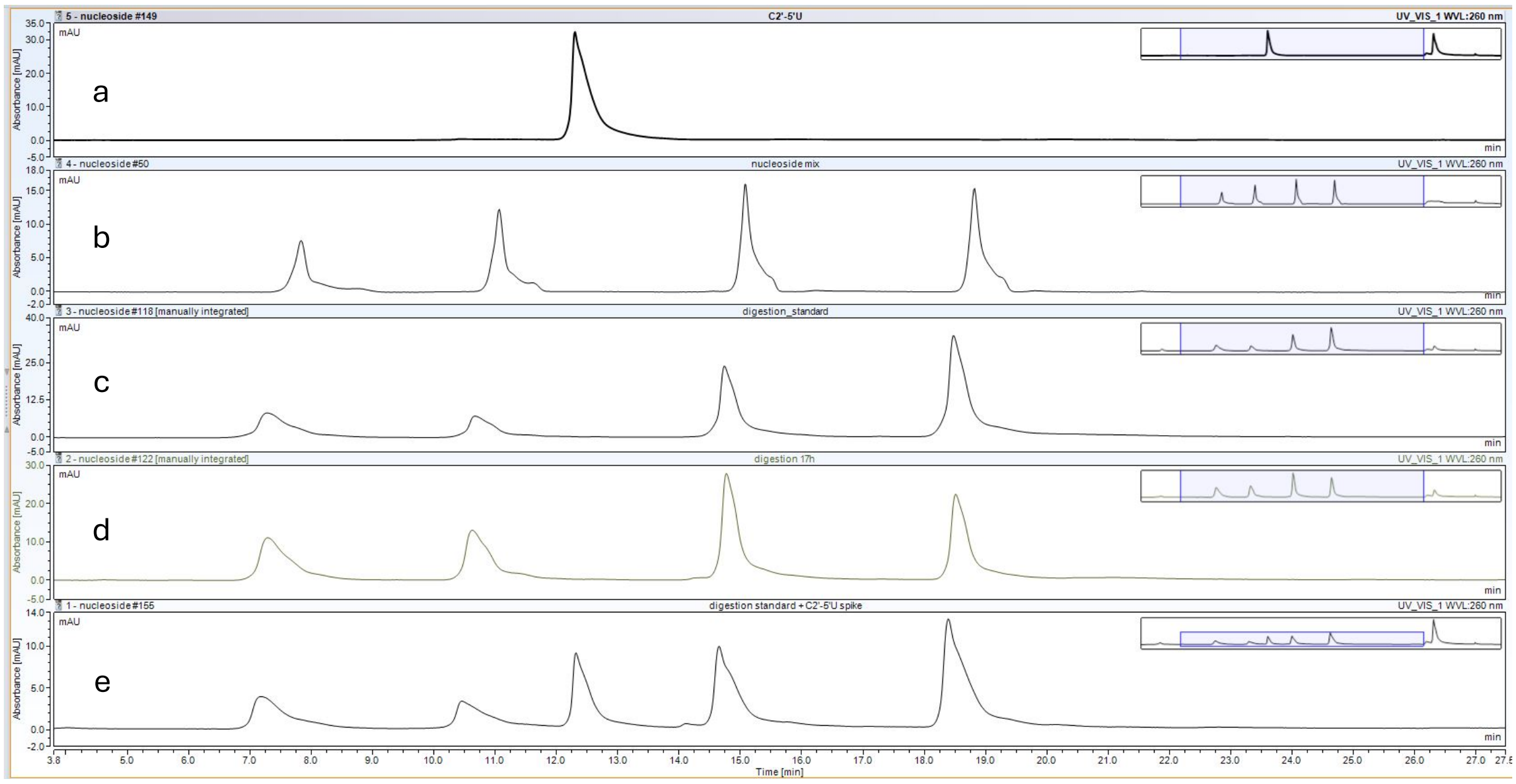

Fig. S43: HPLC traces:

a) synthetic standard of the $C^{2'-5'}U$ dinucleotide

b) mixture of nucleosides (in order of peaks: cytidine, uridine, guanosine, and adenosine)

c) Digestion products of the synthetic fully 3′-5′ linked 15mer 5′-GAGAACCUACUGGCA

d) Digestion products of the 15mer 5′-GAGAACCUACUGGCA isolated from the ligation reaction

e) Digestion products spiked with the synthetic standard of the $C^{2'-5'}U$ dinucleotide

Mechanism of chemical RNA ligation in a nicked duplex using NCI/Mn^{2+}/imidazole

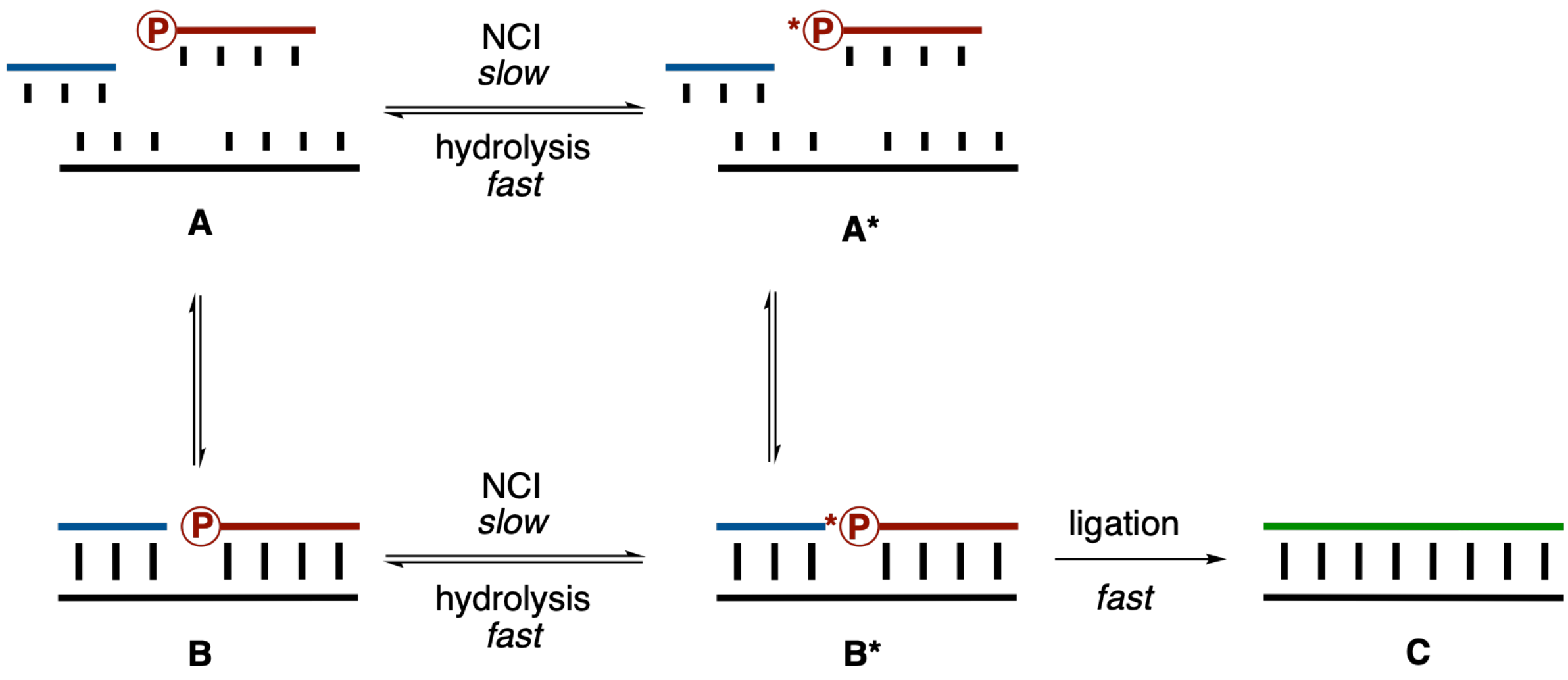

Fig. S44 Reaction scheme for the chemical RNA ligation in a nicked duplex. *Indicates a species in which a phosphate group has been activated by addition to NCI.

In solution there is an equilibrium between the free oligos in solution **A** and the annealed nicked duplex **B**. Once NCI is added to the reaction mixture the phosphate activation can take place. We recognise the activation of the phosphate can take place when the oligo is in solution to give **A***, bound in the nicked duplex to give **B*** or, in one case of sequential binding of the primer and ligator to the template, when the phosphate is bound as a part of a recessed duplex (omitted from reaction scheme in the interest of clarity).

Ferris demonstrated a small effect of M^{2+} ions on activation of the nucleoside-3′-phosphates followed by cyclisation to the 2′,3′-cyclic phosphate, but the difference from the reaction under metal-free conditions resulted in a only modest increase in yield (e.g.: metal free 52%, Mg^{2+} 50%, Mn^{2+} 72%). We have also shown that in the absence of Mn^{2+} activation still occurs as evidenced by the catalysis of NCI hydrolysis by 5′-phosphorylated (oligo)nucleotides (ESI Fig.S25).

Murayama *et al* have previously proposed the role of the metal ions in bringing the phosphate and NCI into proximity to enable the activation to take place.[20] We built a model of this putative complex, here represented by a carefully drawn ChemDraw diagram **M-1**. This clearly illustrates that the distance between the phosphate oxygen and the nitrile of NCI when bound to a metal ion is too great to allow for reaction to occur and the angle between the two is unfavourable. Furthermore, the pK_{aH} of NCI is estimated to be around 1, hinting at the fact it

would probably make a rather poor ligand for any metal ion. We instead propose that it is the hydrated metal-phosphate complex **M-2** which enables cooperative catalysis in the reaction of phosphate and NCI by decreasing the pK_a of bound water allowing for the protonation of the developing negative charge on the nitrile N as the phosphate attacks.

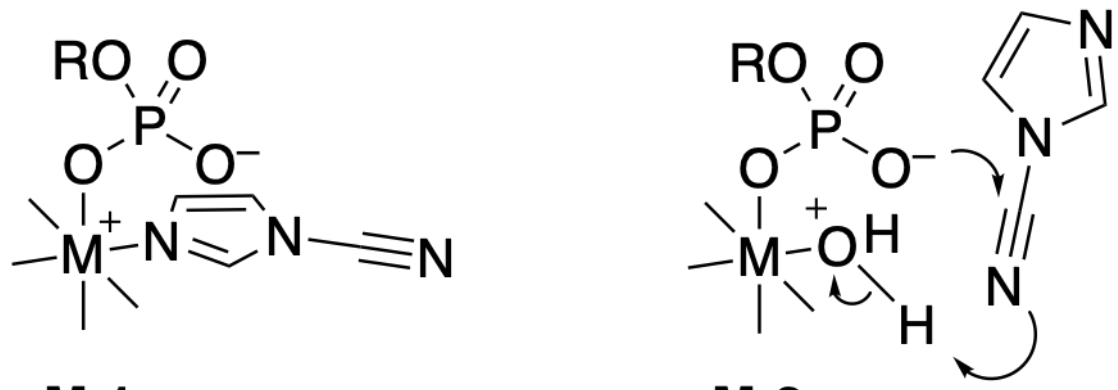

M-1 **M-2**

Fig. S45 Previously proposed M^{2+}/NCI/phosphate metal complex **M-1** and the M^{2+}/phosphate complex **M-2** proposed herein.

Once the phosphate is activated it can undergo rapid hydrolysis. We postulated that the rate of off-template hydrolysis of **A*** is higher than the rate of hydrolysis of template bound activated phosphate **B***. We propose that the template bound activated phosphate B* forms a complex with Mn^{2+} (Fig. 3) that allows for an in-line displacement by the 3′-hydroxyl group of the bound primer to give the ligation product **C**.

We recognise the possibility for both **A*** and **B*** to undergo reaction with imidazole to give the respective unbound or bound 5′-phosphorimidazolides. However, we believe our experimental evidence in closely related systems suggests this is slow in comparison to competing hydrolysis and/or ligation.

Ligation with longer template:

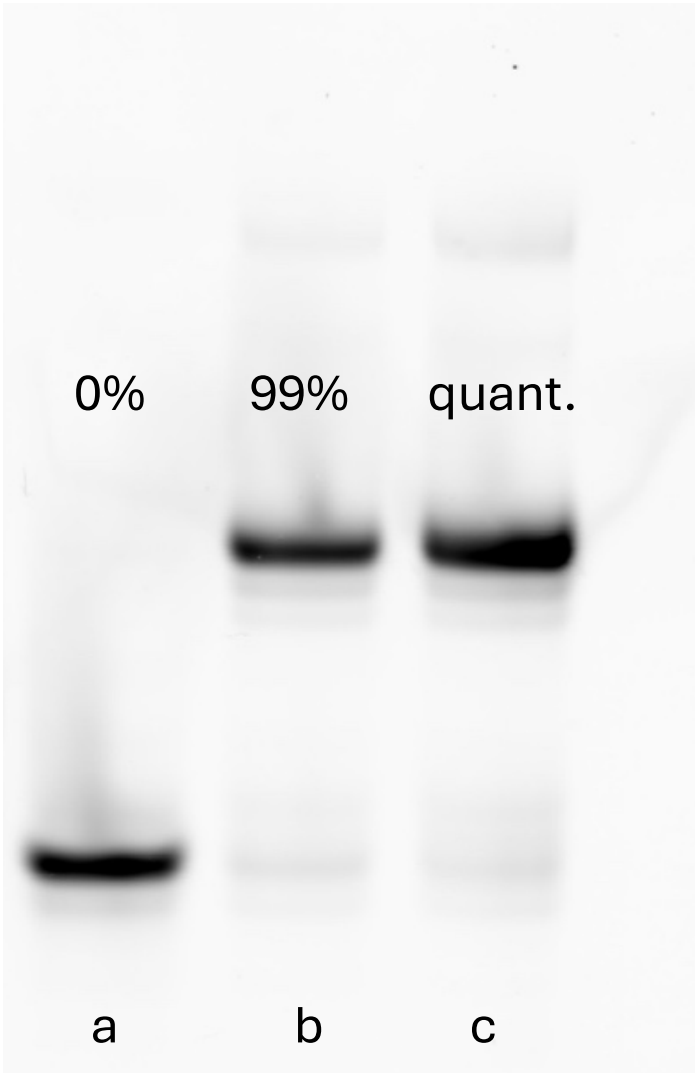

Ligation product

Ligator

Fig. S46 PAGE analysis of in situ nicked-duplex RNA ligation using 5′-pUACUGGCA-Cy3 (10 µM), 5′-GAGAACC (20 µM), 5′-GGUUGCCAGUAGGUUCU (10 µM), NaCl (100 mM), $MnCl_2$ (20 mM), NCI (20 mM) , and imidazole pH 6.2 (20 mM).

a) incubated for 0 h

b) incubated for 24 h

c) incubated for 46 h

Control reaction in the absence of template:

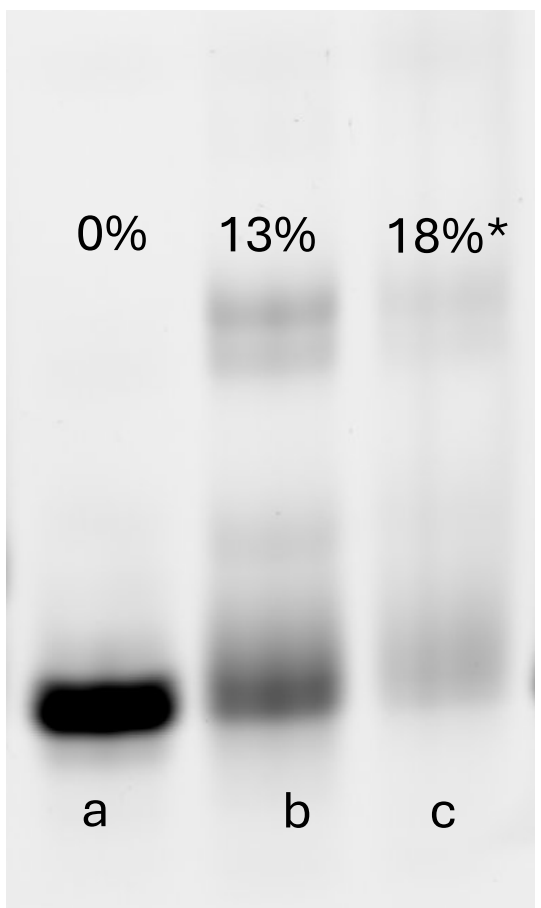

Ligator

Fig. S47 PAGE analysis of in situ nicked-duplex RNA ligation using 5′-pACC (100 µM), 5′-pUACUGGCA-Cy3 (10 µM), NaCl (100 mM), $MnCl_2$ (20 mM), NCI (20 mM), and imidazole pH 6.2 (20 mM).

a) incubated for 0 h

b) incubated for 24 h

c) incubated for 46 h. *substantial decrease in overall fluorescence observed.

Ligation of shorter oligos: 5mer

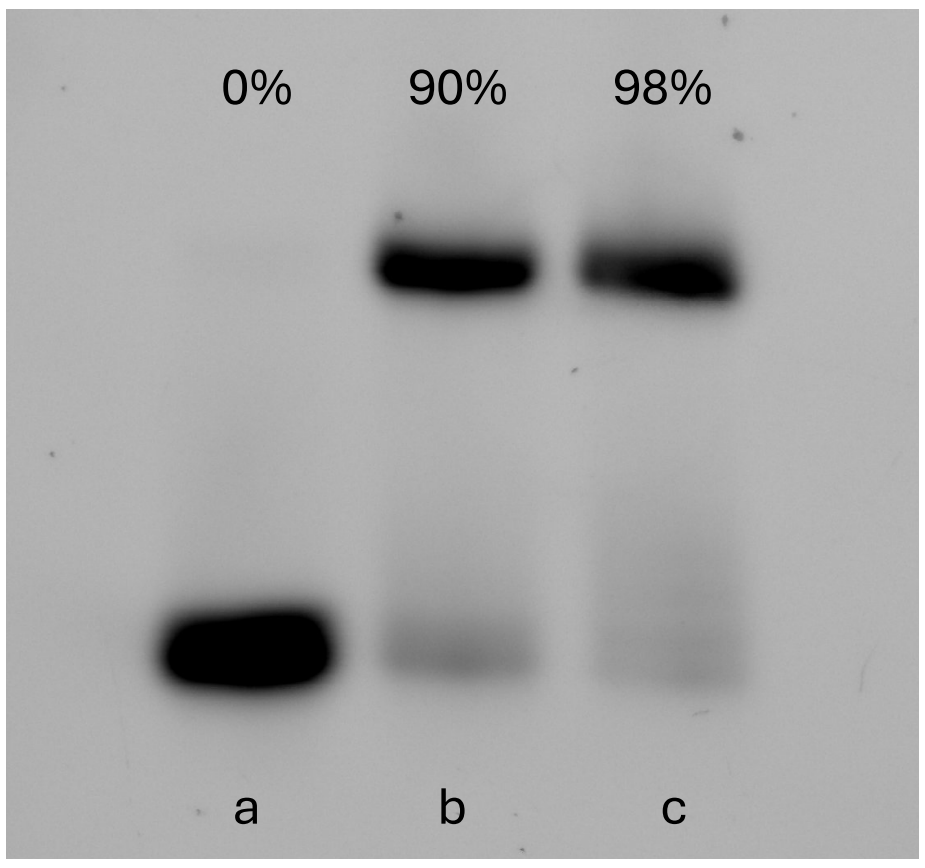

Fig. S48 PAGE analysis of in situ nicked-duplex RNA ligation using 5′-pUACUG-Cy3 (10 µM), 5′-GAGAACC (20 µM), 5′ -CCAGUAGGUUCUC (10 µM), NaCl (100 mM), $MnCl_2$ (20 mM), NCI (20 mM), and imidazole pH 6.2 (20 mM).

a) incubated for 0 h

b) incubated for 24 h

c) incubated for 48 h

Ligation of shorter oligos: 3mer

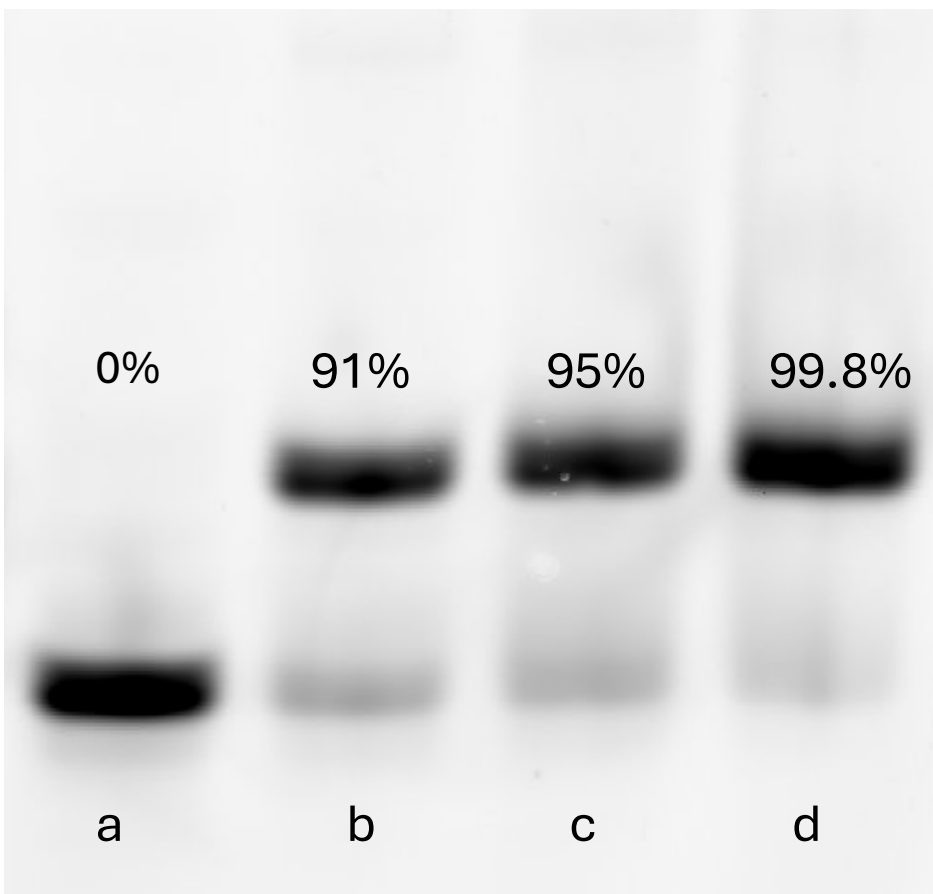

Fig. S49 PAGE analysis of in situ nicked-duplex RNA ligation using 5′-ACC (100 µM), 5′-pUACUGGCA-Cy3 (10 µM), 5′-GGUUGCCAGUAGGUGGU (20 µM), NaCl (100 mM), $MnCl_2$ (20 mM), NCI (20 mM), and imidazole pH 6.2 (20 mM).

a) incubated for 0 h

b) incubated for 19 h

c) incubated for 45 h

d) incubated for 66 h

Ligation of shorter oligos: 3mer with 5′-phosphate, 3′ to 5′ direction

Ligation product

Ligator

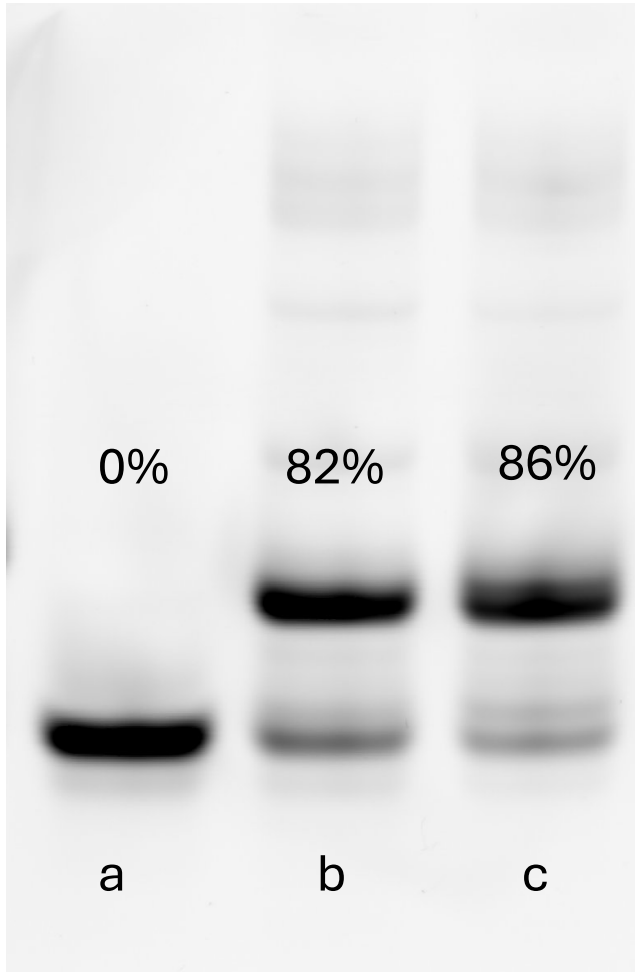

Fig. S50 PAGE analysis of in situ nicked-duplex RNA ligation using 5′-pACC (100 µM), 5′-pUACUGGCA-Cy3 (10 µM), 5′-GGUUGCCAGUAGGUUCU (20 µM), NaCl (100 mM), $MnCl_2$ (20 mM), NCI (20 mM), and imidazole pH 6.2 (20 mM).

a) incubated for 0 h

b) incubated for 24 h

c) incubated for 46 h

Ligation of shorter oligos: 3mer with 5′-phosphate, 5′ to 3′ direction

Ligation product

Primer

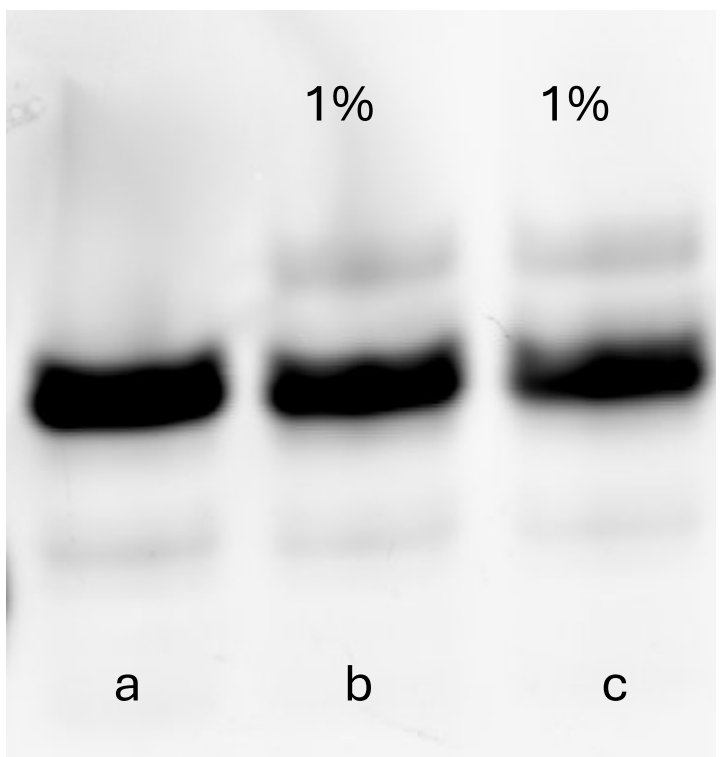

Fig. S451 PAGE analysis of in situ nicked-duplex RNA ligation using 5′-pACC (100 µM), 5′-Cy3-ACCUACUGGCA (10 µM), 5′-GGUUGCCAGUAGGUUCU (20 µM), NaCl (100 mM), $MnCl_2$ (20 mM), NCI (20 mM), and imidazole pH 6.2 (20 mM).

a) incubated for 0 h

b) incubated for 20 h

c) incubated for 46 h

Ligation of 5mer ligator with 3mer primer

Ligation product

Ligator

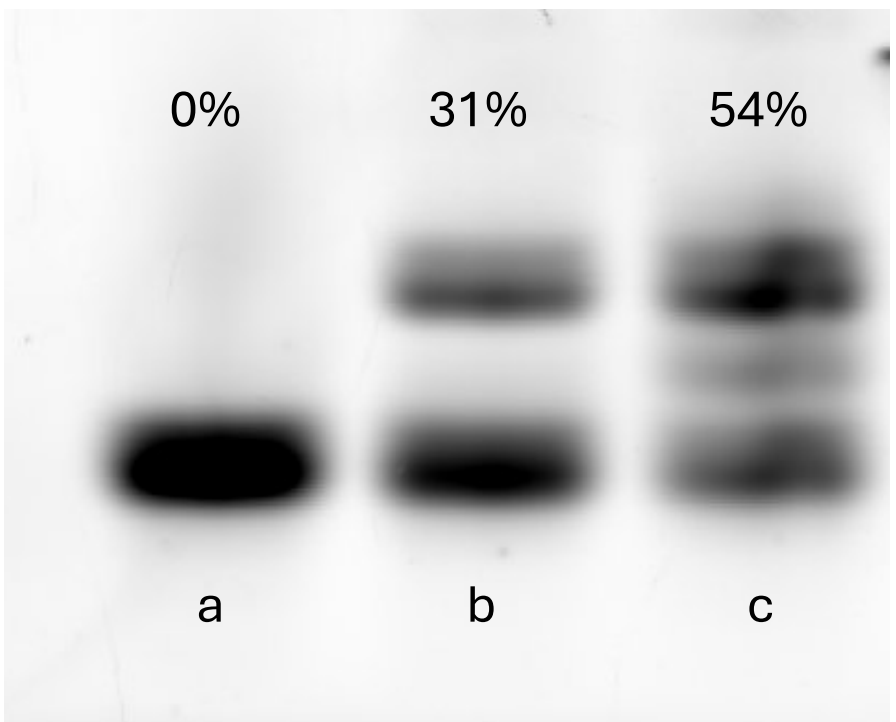

Fig. S52 PAGE analysis of in situ nicked-duplex RNA ligation using 5′-pACC (100 µM), 5′-pUACUG-Cy3 (10 µM), 5′-GGUUGCCAGUAGGUUCU (20 µM), NaCl (100 mM), $MnCl_2$ (20 mM), NCI (20 mM), and imidazole pH 6.2 (20 mM), performed at 4 °C.

a) incubated for 0 h

b) incubated for 24 h

c) incubated for 48 h

Ligation of two consecutive 3mers

Ligation of two 3mers

Ligation of one 3mer

Ligator

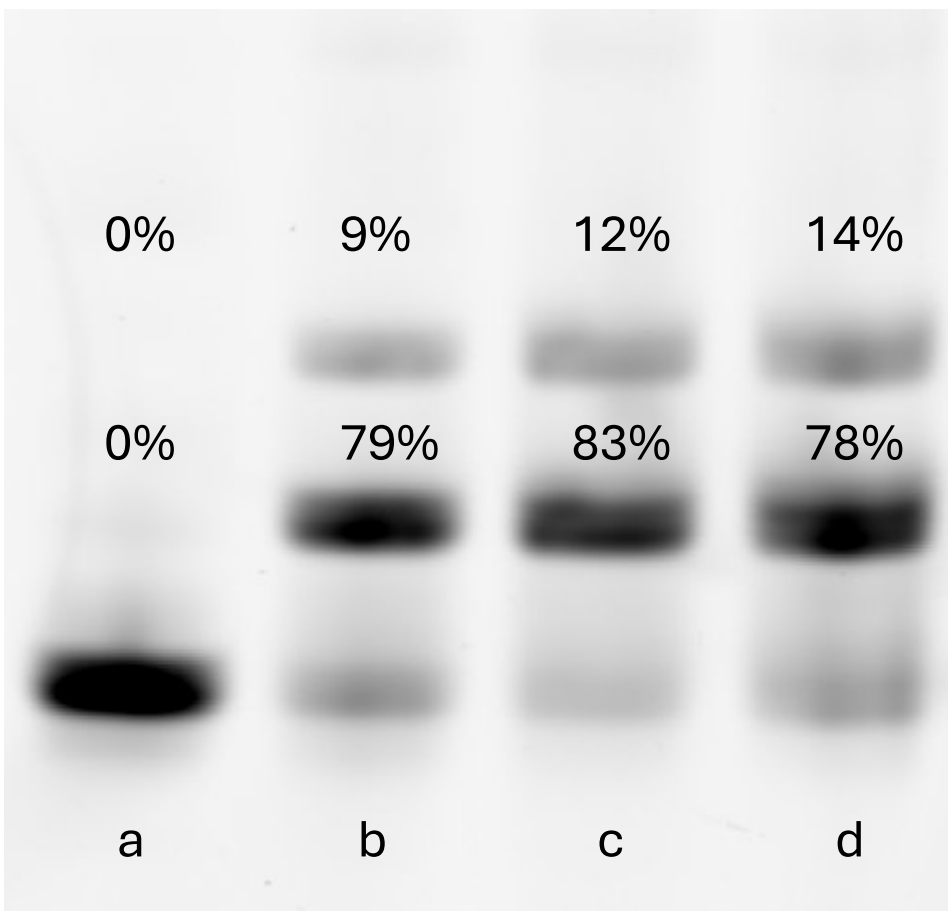

Fig. S53 PAGE analysis of in situ nicked-duplex RNA ligation using 5′-pACC (100 µM), 5′-pUACUGGCA-Cy3 (10 µM), 5′-GGUUGCCAGUAGGUGGU (20 µM), NaCl (100 mM), $MnCl_2$ (20 mM), NCI (20 mM), and imidazole pH 6.2 (20 mM).

a) incubated for 0 h

b) incubated for 19 h

c) incubated for 45 h

d) incubated for 66 h

Ligation of shorter oligos: dimer

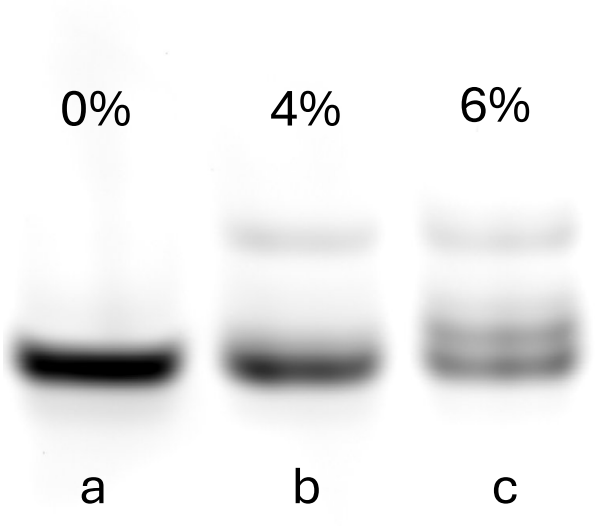

Fig. S54 PAGE analysis of in situ nicked-duplex RNA ligation using 5′-CC (100 µM), 5′-pUACUGGCA-Cy3 (10 µM), 5′-GGUUGCCAGUAGGUUCU (20 µM), NaCl (100 mM), $MnCl_2$ (20 mM), NCI (20 mM), and imidazole pH 6.2 (20 mM).

a) incubated for 0 h

b) incubated for 24 h

c) incubated for 46 h

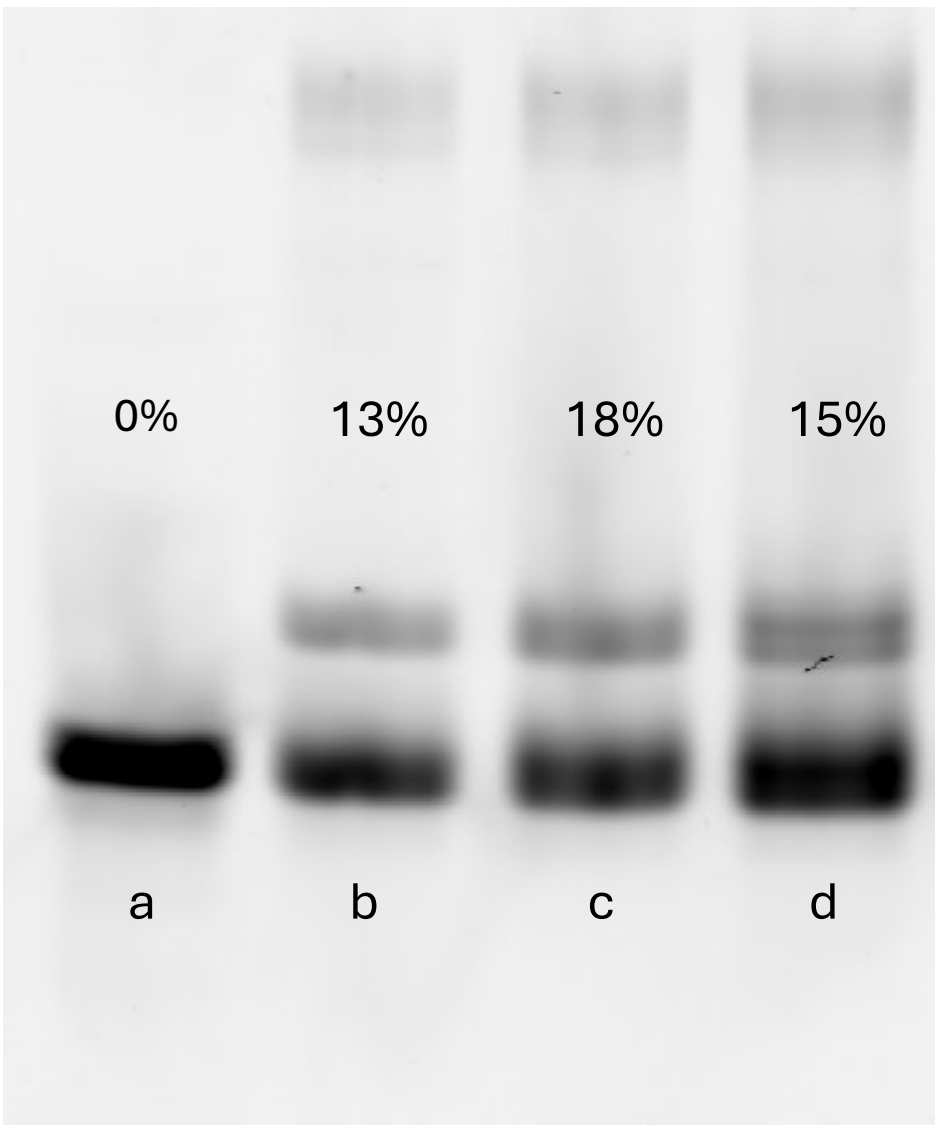

Fig. S55 PAGE analysis of in situ nicked-duplex RNA ligation using 5′-CC (1 mM), 5′-pUACUGGCA-Cy3 (10 µM), 5′-GGUUGCCAGUAGGUUCU (10 µM), NaCl (100 mM), $MnCl_2$ (20 mM), NCI (20 mM), and imidazole pH 6.2 (20 mM).

a) incubated for 0 h

b) incubated for 24 h

c) incubated for 48 h

d) incubated for 68 h

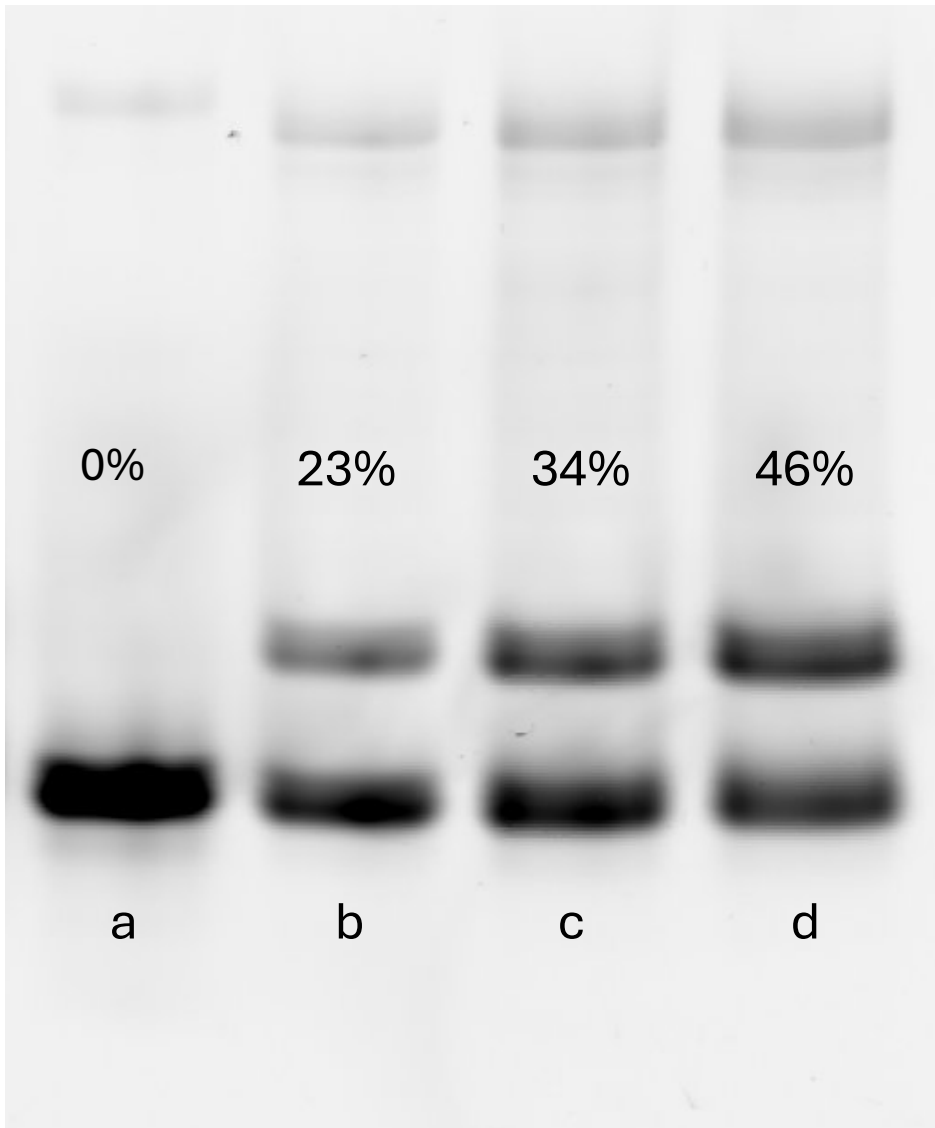

Fig. S56 PAGE analysis of in situ nicked-duplex RNA ligation using 5′-CC (1 mM), 5′-pUACUGGCA-Cy3 (10 μM), 5′-GGUUGCCAGUAGGUUCU (10 μM), NaCl (100 mM), $MnCl_2$ (20 mM), NCI (20 mM), and imidazole pH 6.2 (20 mM), at 4 °C.

a) incubated for 0 h

b) incubated for 24 h

c) incubated for 48 h

d) incubated for 68 h

Referencesh

(1) Beck, M. T.; Gáspár, V.; Llng, J. Formation of Glycine in the Hydrolysis of Coordinated Cyanogen. *Inorganica Chimica Acta* **1979**, 33, L177-L178.

(2) Tobia, S. K.; El-Shahat, M. F. A New Class of Metal Cyano-Complexes. Part 1. Preparation and Nature of the Copper Complex. *J. Chem. Soc. A* **1968**, 2444-2447.

(3) Ritson, D.; Sutherland, J. D. Prebiotic Synthesis of Simple Sugars by Photoredox Systems Chemistry. *Nat. Chem.* **2012**, 4, 895–899.

(4) Liu, Z.; Mariani, A.; Wu, L.; Ritson, D.; Folli, A.; Murphy, D.; Sutherland, J. Tuning the Reactivity of Nitriles Using Cu(II) Catalysis – Potentially Prebiotic Activation of Nucleotides. *Chem. Sci.* **2018**, 9, 7053–7057.

(5) Yi, R.; Hongo, Y.; Fahrenbach, A. C.; Li, R. Synthesis of Imidazole-Activated Ribonucleotides Using Cyanogen Chloride. *Chem. Commun.* **2018**, 54, 511–514.

(6) Ferris, J. P.; Huang, C. H.; Hagan, W. J. *N*-Cyanoimidazole and Diimidazole Imine: Water-Soluble Condensing Agents for the Formation of the Phosphodiester Bond. *Nucleosides and Nucleotides* **1989**, 8, 407–414.

(7) Kanaya, E.; Yanagawa, H. Template-Directed Polymerization of Oligoadenylates Using Cyanogen Bromide. *Biochemistry* **1986**, 25, 7423–7430.

(8) Keefe, A. D.; Miller, S. L. Potentially Prebiotic Syntheses of Condensed Phosphates. Origins Life Evol. Biosphere **1996**, 26, 15–25.

(9) Liu, Z.; Wu, L. F.; Bond, A. D.; Sutherland, J. D. Photoredox Chemistry in the Synthesis of 2-Aminoazoles Implicated in Prebiotic Nucleic Acid Synthesis. *Chem. Commun.* **2020**, 56, 13563–13566.

(10) Liang, M. C.; Hartman, H.; Kopp, R. E.; Kirschvink, J. L.; Yung, Y. L. Production of Hydrogen Peroxide in the Atmosphere of a Snowball Earth and the Origin of Oxygenic Photosynthesis. *Proc. Natl. Acad. Sci. U. S. A.* **2006**, 103, 18896–18899.

(11) Koeppe, R. E.; Wood, J. L. Syntheses of Some 2-ThiocyanoimidazoIes. *J. Am. Chem. Soc.* **1953**, 75, 4655–4657.

(12) Cataldo, F. 13C NMR and FT-IR Spectra of Thiocyanogen, S2(CN)2, Selenocyanogen, Se2(CN)2, and Related Compounds. *Polyhedron* **2000**, 19, 681–688.

(13) Nagy, P.; Alguindigue, S. S.; Ashby, M. T. Lactoperoxidase-Catalyzed Oxidation of Thiocyanate by Hydrogen Peroxide: A Reinvestigation of Hypothiocyanite by Nuclear Magnetic Resonance and Optical Spectroscopy. *Biochemistry* **2006**, 45, 12610-12616.

(14) Oulego, P.; Collado, S.; Laca, A.;Díaz, M. Iron (II) as catalyst for thiocyanate wet oxidation: Mechanism and modelization. *Chem. Eng. J.* **2017**, 316, 812-822.

(15) Inoue, T.; Orgel, L. E. Substituent Control of the Poly(C)-Directed Oligomerization of Guanosine 5'-Phosphoroimidazolide. *J. Am. Chem. Soc.* **1981**, 103, 7666–7667.

(16) Liu, Z.; Jiang, C. Z.; Bond, A. D.; Tosca, N. J.; Sutherland, J. D. Manganese(II) Promotes Prebiotically Plausible Non-Enzymatic RNA Ligation Reactions. *Chem. Commun.* **2024**, 60, 6528–6531.

(17) Butzow, J. J.; Eichhorn, G. L. Interaction of Metal Ions with Nucleic Acids and Related Compounds. XVII. On the Mechanism of Degradation of Polyribonucleotides and Oligoribonucleotides by Zinc (II) Ions. *Biochemistry* **1971**, 10, 2019–2027.
